# Assessment of arsenic, cadmium, lead, mercury, and per- and polyfluoroalkyl substances concentrations in human milk and infant formula in the United States: a systematic review

**DOI:** 10.1016/j.ajcnut.2025.07.039

**Published:** 2025-09-23

**Authors:** Rachel C Thoerig, Lauren E O’Connor, Maureen K Spill, Arin A Balalian, Rupal Trivedi, Shailesh M Advani, Cassi N Uffelman, Trish Bosse, Margaret J Foster, Kyle M Holland, Kathryn G Dewey, Mandy M Fisher, Aubrey L Galusha, Carin A Huset, Amanda J MacFarlane

**Affiliations:** 1 Agriculture, Food, and Nutrition Evidence Center, Fort Worth, TX, United States; 2 Center for Systematic Reviews and Research Syntheses, Medical Sciences Library, Texas A&M University, College Station, TX, United States; 3 Department of Nutrition, University of California-Davis, Davis, CA, United States; 4 Environmental Health Science and Research Bureau, Health Canada, Ottawa, ON, Canada; 5 Wadsworth Center, New York State Department of Health, Albany, NY, United States; 6 Department of Environmental Health Sciences, College of Integrated Sciences, University at Albany, Rensselaer, NY, United States; 7 Public Health Laboratory, Minnesota Department of Health, Saint Paul, MN, United States; 8 Department of Nutrition, Texas A&M University, College Station, TX, United States; 9 Nutrition Research Division, Health Canada, Ottawa, ON, Canada

**Keywords:** human milk, infant formula, infant feeding, arsenic, cadmium, lead, mercury, PFAS, heavy metal, breast milk

## Abstract

**Background:**

Foods, including human milk (HM) and infant formula (IF), can be sources of environmental contaminant exposure for infants, which can impact health and development.

**Objectives:**

This systematic review describes arsenic, cadmium, lead, mercury, and per- and polyfluoroalkyl substances (PFAS) concentrations in HM and IF in the United States (PROSPERO #CRD42024528756).

**Methods:**

We searched CAB Abstracts, CENTRAL, CINAHL, Embase, and MEDLINE for peer-reviewed articles published in English through 2 April, 2025 (no date restrictions). Studies that assessed contaminant concentrations in HM or IF from countries rated as “high” or “very high” on the Human Development Index were eligible. Screening, data extraction, and risk of bias assessments were performed by 2 independent reviewers. We narratively synthesized United States studies and assessed the certainty of evidence with Grading of Recommendations Assessment, Development, and Evaluation (GRADE). We developed heat maps for studies from all countries that may help inform evidence gaps in future systematic reviews.

**Results:**

From the United States, 14 HM and 16 IF studies were included. For HM, perfluorooctanoic acid (PFOA) concentrations ranged from undetected to 36.1 pg/mL, and perfluorooctane sulfonic acid (PFOS) ranged from undetected to 106 pg/mL (GRADE: moderate); evidence was lacking for perfluorononanoic acid and perfluorohexanesulfonic acid. For IF, all PFAS were largely undetected (GRADE: moderate). For HM and IF, studies for arsenic, cadmium, lead, and mercury had small and unrepresentative samples, and most were published before 2000. We identified 317 and 108 articles for HM and IF, respectively, from other countries.

**Conclusions:**

In published, peer-reviewed United States studies, PFOA and PFOS were detected in HM; PFAS were largely undetected in IF. There was a paucity of contemporary evidence for arsenic, cadmium, lead, and mercury in HM or IF in the United States, but we identified evidence from other countries that could help inform these knowledge gaps. Public health agencies recommend feeding infants HM given the benefits outweigh potential risks of contaminant exposure.

This trial was registered at PROSPERO as CRD42024528756 (https://www.crd.york.ac.uk/PROSPERO/view/CRD42024528756).

## Introduction

Environmental contaminants can be introduced into the food supply through air, water, and soil or during manufacturing or packaging [1]. Infant exposure to environmental contaminants, such as arsenic, lead, cadmium, mercury, and per- and polyfluoroalkyl substances (PFAS; category of over 12,000 “forever chemicals” [2]) can interfere with healthy development [1]. Food is a known source of exposure to these contaminants [1]. Human milk (HM) and infant formula (IF) are the main foods consumed by infants in the United States [3]. Thus, it is critical to understand potential contaminants in HM and IF and how these foods potentially contribute to overall contaminant exposure from all sources (e.g., air, dust, household products) for infants in the United States.

HM is recommended as the main food source for infants from birth to ∼6 mo of age [4,5]. More than 80% of United States infants initially consume HM and ∼25% exclusively consume HM until 6 mo [6]. HM consumption is associated with myriad benefits, including immune system development [7], respiratory health [8], and lower risk of infections, gastrointestinal illness, sudden infant death syndrome, obesity [9], type 1 diabetes, and childhood leukemia [10]. There is guidance for prescription medications, caffeine, and alcohol use during lactation [11] to reduce infant exposure to harmful compounds that may transfer into HM. However, no public health program or policy currently exists in the United States for monitoring environmental contaminants in HM [[12], [13], [14]]. Unlike medications, caffeine, and alcohol, environmental contaminants are largely unavoidable. Therefore, research is needed to understand the prevalence of these contaminants in HM to guide future screening and monitoring during pregnancy and lactation.

IFs provide an alternative food source to HM (15) and are consumed by 67% of United States infants by 3 mo of age [11]. The United States Food and Drug Administration (FDA) has regulated IF in the United States since 1941 to maintain a safe and nutritious supply [16]. The FDA works with manufacturers to monitor and respond to a variety of different environmental contaminants [17,18] that can be introduced via ingredient sourcing, machinery, packaging, or water sources [[19], [20], [21]]. Contamination of IF can lead to recalls and shortages which may require increasing imports from other countries [22]. Evidence from other countries about environmental contaminants in IF can contribute to the knowledge base to ensure a safe and reliable IF supply in the United States. Federal initiatives, such as Operation Stork Speed announced in March 2025, call for more contaminant testing in IF and greater transparency from the IF industry [23].

Current initiatives for monitoring or reducing contaminant exposure from foods for infants are largely focused on complimentary foods (i.e., infant foods, snacks, cereals) other than HM or IF [24,25]. There are no current mandatory federal limits or proposed action levels for arsenic, cadmium, lead, mercury, or PFAS in IF, and monitoring of these contaminants in HM is lacking [1]. Therefore, our objectives were to conduct a systematic review to *1*) describe concentrations of arsenic, cadmium, lead, mercury, and select PFAS in HM and IF in the United States and *2*) document available literature on these contaminants in HM and IF from other countries to identify opportunities for future systematic reviews that could help inform United States policies.

## Methods

Our systematic review protocol was registered a priori on PROSPERO (CRD42024528756) and was part of 6 systematic reviews commissioned by the FDA’s Human Foods Program within its mission area of food chemical safety [26] (Supplemental Table 1). This review pertains to the question*: What is the composition/variability of contaminants in HM and IF?* Evidence related to the transfer of maternal exposure into HM, infant overall exposure, and bioavailability of contaminants in HM and IF are not described in this manuscript. The review questions, search strategy, inclusion/exclusion criteria, risk of bias (ROB) assessment, plans for synthesis, and causes of heterogeneity were informed by a Technical Expert Panel (TEP) with expertise in human biomonitoring, infant nutrition, and HM (KGD, MMF). The protocol adhered to the PRISMA guidelines [27] (Appendix A) and addressed the A MeaSurement Tool to Assess systematic Reviews 2 (AMSTAR 2) criteria for a high-quality review (Appendix B) [28].

### Search strategy

The search strategy for this systematic review was used to inform multiple research questions addressed in the broader project (Supplemental Table 1). The search strategy was developed by 2 evidence synthesis information specialists (MJF, KMH) and was peer reviewed by a third (Supplemental Table 2). Five databases were searched for peer-reviewed articles published in English: Medline (Ovid), Embase (Ovid), CAB Abstracts (Ovid), CINAHL (EBSCO), and CENTRAL (Cochrane Library). Searches were completed on 2 April, 2025, with no date restrictions. Backward citation searches were conducted independently by 2 reviewers for relevant titles referenced in the bibliographies of included articles. Additionally, articles in the *Journal of Environmental Exposure Assessment* were searched through the journal’s search engine based on pertinent terms because it was not indexed in the searched databases and was identified by a TEP member as a relevant journal (Supplemental Table 2).

Eligible articles reported measured concentrations of the following contaminants: arsenic (total and inorganic), cadmium, lead, mercury (total and methylmercury), select PFAS [perfluorooctanoic acid (PFOA), perfluorooctane sulfonic acid (PFOS), perfluorononanoic acid (PFNA), and perfluorohexanesulfonic acid (PFHxS)] in HM or IF from countries rated as “high” or “very high” on the Human Development Index (HDI) [29]. PFOA, PFOS, PFNA, and PFHxS were prioritized because they *1*) have toxicological reference values used to assess concentrations found in food due to environmental contaminants, *2*) are well studied, and *3*) have accetpable analytic methods [[30], [31], [32], [33]]. Contaminant concentration data from randomized and nonrandomized designs were deemed appropriate by the TEP to address the research question, which included randomized controlled trials, nonrandomized controlled trials, quasi-experimental studies, prospective or retrospective cohort studies, cross-sectional studies, and descriptive studies or other designs without a comparison group.

### Screening and data extraction

Screening forms were developed based on the inclusion and exclusion criteria (Supplemental Table 3) and were piloted on 25 articles by reviewers to ensure consistency. Two reviewers independently screened records identified in the search using DistillerSR (Ottawa, ON: Evidence Partners; 2020) at the title/abstract and full-text levels. All records were dually, independently reviewed, and disagreements were resolved by a third reviewer or through discussion.

This search was used to inform 5 of the 6 systematic review questions, so records were included at title/abstract screening if arsenic, cadmium, lead, mercury, or PFAS were measured in HM and/or IF. During the initial full-text screening, high-level data were independently extracted by 2 reviewers, which included the contaminant measured, if it was measured in HM and/or IF, and the country of data collection. Data extraction conflicts were resolved by a third reviewer. On the basis of these data, Stata (Stata Corp) was used to separate articles into datasets specific to each systematic review question for a second round of full-text screening to confirm that criteria were met for each research question. Eligible studies that assessed contaminant concentrations in HM or IF are described in this manuscript.

For the United States studies, more detailed data were extracted for synthesis, as outlined in the analytic framework (Supplemental Figure 1). The following qualitative data were extracted by 1 reviewer and quality checked by a second: study design, study or cohort name, date and location of data collection, funding source, characteristics of participants who provided HM samples (age, prior parity, prior lactation, body composition, demographics, and nutrition or health status) and exposure source, HM sample collection procedures and analytic techniques, IF product characteristics, preparation methods, and water source, and IF sampling strategies and analytic techniques. The following quantitative data were extracted independently by 2 reviewers and cross-checked by a third: contaminant concentrations of HM or IF, sample size, and results of statistical analyses.

For concentration data, medians were prioritized because biological data are often skewed. However, arsenic, cadmium, lead, and mercury were most often reported as means. To ease comparison across studies within each contaminant, we extracted means for arsenic, cadmium, lead, and mercury and converted the median to mean in 1 study using the Meta-Analysis Accelerator conversion calculator [34]. Study authors were not contacted about missing data because our objective was to summarize studies published data in the peer-reviewed literature and thus retrieving unpublished data was out of scope.

### Risk of bias

For HM, we assessed ROB using Risk of Bias 2 for randomized controlled trials (RoB 2) [35], Risk of Bias In Non-randomized Studies of Interventions (ROBINS-I) [36,37], or the Risk Of Bias In Non-randomized Studies of Exposure (ROBINS-E) tool [38], depending on the study design. Operationalization is described in Supplemental Table 4. Individual domains were assessed to determine an overall ROB rating for the article, including selection bias (i.e., whether those that provided a HM sample represent the source population and whether authors described differences between those that supplied a HM sample compared with those that did not), missing data, measurement of outcome (i.e., contaminants in HM), and omission of reported results. Domains related to confounding, exposure assessment, and postintervention exposure were considered at low risk across studies because they were not considered to be sources of bias due to the descriptive nature of the analytic framework, as determined by the TEP (Supplemental Figure 1). The reviewers piloted the assessments on 2–3 articles to ensure a consistent approach.

For IF, we developed a ROB assessment by considering sampling strategy (i.e., were the samples selected in a way that was representative of IF in the United States market including main ingredients, brands, manufacturers as well as if purchasing location and time of year were described), missing data, outcome assessment (i.e., contaminants in IF), and reporting bias (Supplemental Table 5). The assessment was piloted on 3 articles per tool relevant to the study design of included articles across all ROB reviewers to ensure consistency. Domains were rated as high, some concerns, or low ROB.

For both HM and IF, ROB assessments were performed by 2 reviewers independently. Domain-level ratings were compared, and discrepancies were discussed until consensus, or in consultation with a third reviewer, as needed. The highest domain-level ROB rating was used as the overall rating.

### Critical appraisal

Another TEP (ALG, CAH, JLR) with expertise in human biomonitoring and surveillance of environmental toxicants, specifically for heavy metals and PFAS, critically appraised the sample collection and analytic methods used for measuring contaminants in HM and IF. Two experts assessed each article, and articles were categorized into those that were “acceptable” or those that had “some concerns” due to insufficient reporting of methods or quality controls. A third expert resolved any conflicts.

### Data synthesis

For United States studies, data were stratified by HM or IF, and then by contaminant and synthesized narratively. Concentration data were converted, if possible, to the same units to facilitate comparisons. For HM, sources of heterogeneity were described and considered in conclusions, including population characteristics, geographic location, and TEP critical appraisal. For IF, sources of heterogeneity included date and location of data collection, IF type, and TEP critical appraisal. We had concerns about the relevancy of older IF data due to changes in ingredient sourcing, manufacturing processes, and regulations over time. Therefore, all articles were described in the results but conclusions were based on the more contemporary studies published after the year 2000. Details of the studies published before 2000 are shown in supplemental materials. For each conclusion for HM or IF, sensitivity analyses were conducted by excluding articles at high ROB.

### Certainty of evidence

Grading of Recommendations Assessment, Development, and Evaluation (GRADE) [39] was used to assess the certainty of evidence for each contaminant if the evidence supported a conclusion. GRADE considers ROB, inconsistency, imprecision, indirectness, and publication bias. Additional considerations include dose–response, magnitude of effect, and residual confounding, which are used for nonrandomized study designs. However, these latter domains were not considered for this manuscript because the data were descriptive.

### Protocol deviations

As stated in our PROSPERO registration, we included studies from countries that were “high” or “very high” on the HDI. We decided post hoc to synthesize and draw conclusions about contaminant concentrations in HM and IF from United States studies only to ensure that conclusions were relevant to the United States. Articles for all other countries that were “high” or “very high” on the HDI [29], including the United States, were instead presented in heat maps to document data that may help inform evidence gaps for the United States in future systematic reviews.

We stated in our protocol that we would use RoB 2, ROBINS-I, or ROBINS-E to assess the ROB for all studies. However, these tools were developed for human studies, thus, some domains and the signaling questions were not relevant to the IF studies. Therefore, we created an ROB assessment to address sources of bias particular to the IF studies, as described above. We also included CAB Abstracts as an additional database, which helped decrease publication bias by expanding capture of the database search, and an additional extractor for quantitative data for added precision.

We planned a priori to conduct random-effects meta-analyses. However, there were a small number of contemporary studies identified in the search, most of which lacked critical details on the population or samples assessed. Therefore, we determined that meta-analyses were not appropriate. Instead, we conducted a narrative synthesis to best capture the reported results and limitations across all studies. We also decided post hoc to make conclusions about contaminant concentrations in IF only for studies published after 2000, because the IF products assessed in older articles would likely not represent IF available in the present United States market.

## Results

### Contaminants assessed in HM

#### Search results

**FIGURE 1:**
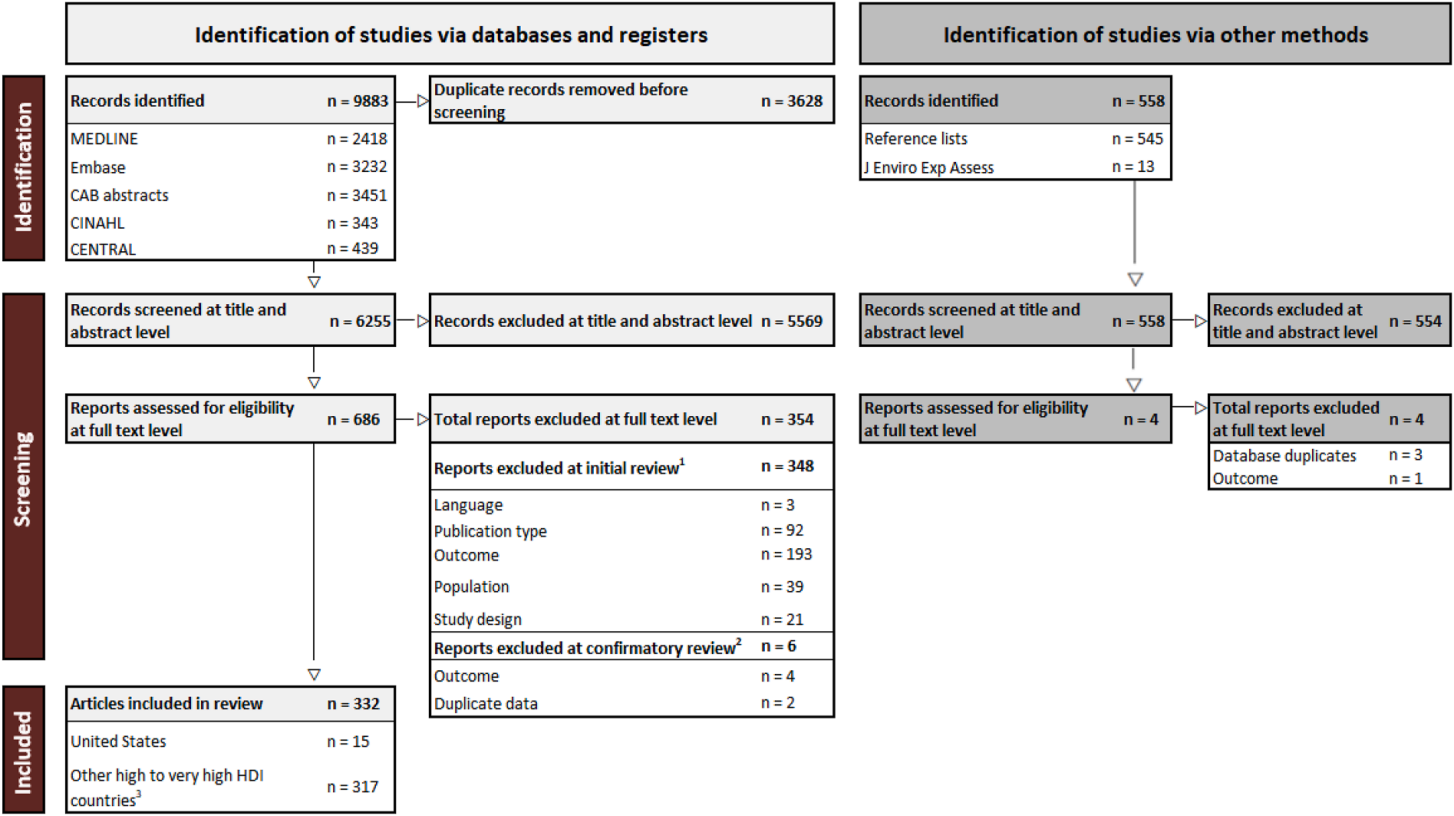
PRISMA diagram for studies that assessed arsenic, cadmium, lead, mercury, and per- and polyfluoroalkyl substances (PFAS) concentrations in human milk samples. ^1^During initial review, data related to the contaminant assessed, whether the contaminant was assessed in human milk, infant formula, and/or infant biospecimens, and the country of data collection were extracted. ^2^The extracted data helped identify articles for inclusion in the 5 systematic review questions. Using Stata (Stata Corp), the articles were separated into datasets specific to each question and were further screened to confirm that all inclusion and exclusion criteria were met. ^3^Non-United States countries rated “high” and “very high” in the Human Development Index (HDI) are only included in the heat maps. The HDI is a measurement of development including: quality of life, education, and quality of living [29]. Database details: CAB Abstracts (Ovid), CENTRAL (Cochrane Library), CINAHL (EBSCO), Embase (Ovid), and Medline (Ovid). J Enviro Exp Assess, Journal of Environmental Exposure Assessment; PRISMA, Preferred Reporting Items for Systematic Reviews and Meta-Analyses.

The database search identified 6255 unique records that were screened at the title/abstract level (Figure 1). During initial full-text screening, 348 of 686 full-text articles were excluded because they were not relevant to HM. The remaining 338 articles underwent a second full-text screening to confirm eligibility. Six articles were excluded at this confirmatory level due to no outcome or duplicative data (Supplemental Table 6). No additional articles were identified from other sources, including backward citation searching or handsearching of the *Journal of Environmental Exposure Assessment*.

#### Contaminants assessed in HM in the United States

**FIGURE 2:**
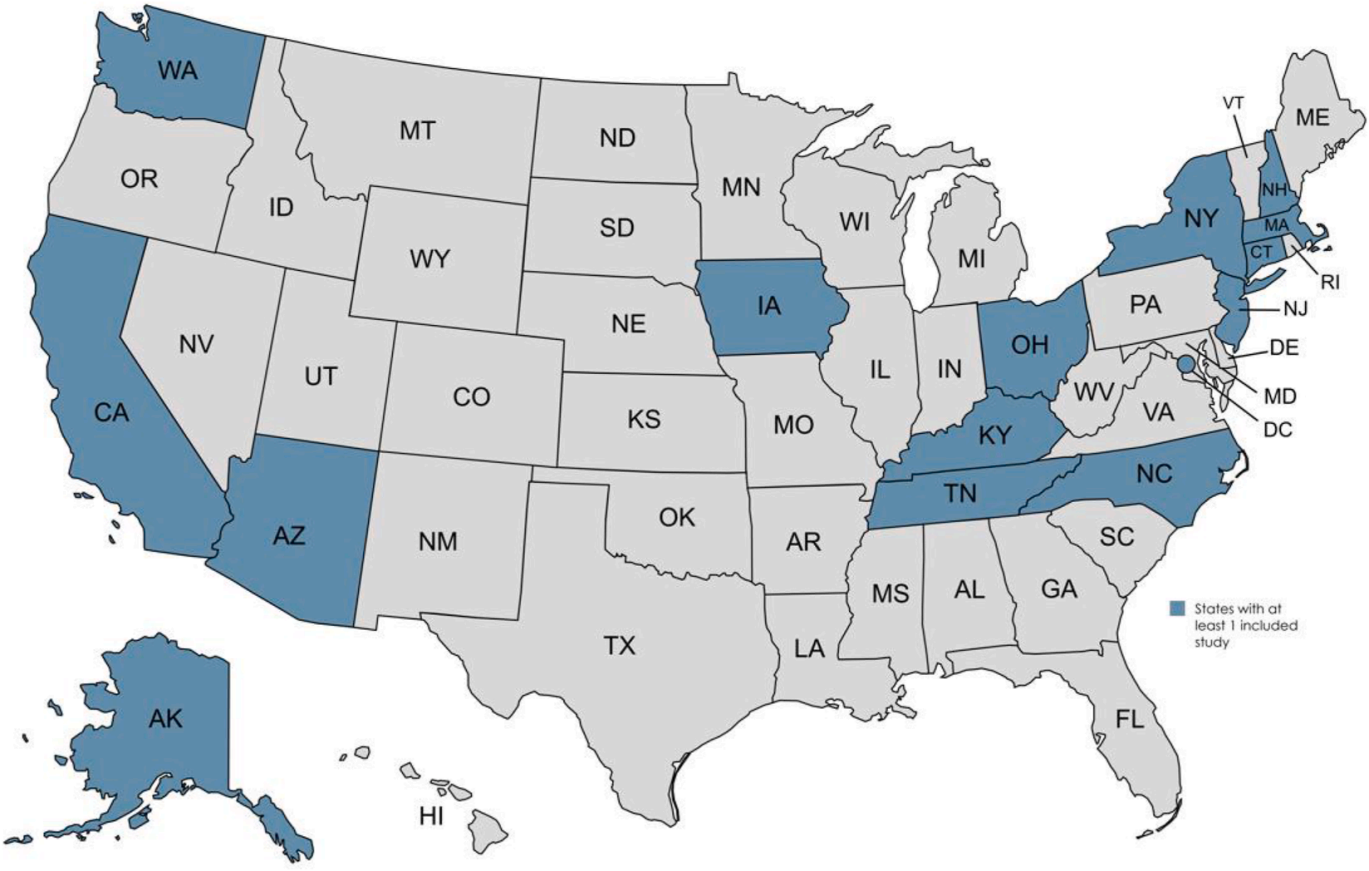
Location of data collection for studies that assessed arsenic, cadmium, lead, mercury, and PFAS concentrations in human milk samples in the United States. Image produced with mapchart.net. PFAS, per- and polyfluoroalkyl substances.

We identified 15 articles from 14 United States studies (*n* = 2 for arsenic [40,41], *n* = 2 for cadmium [41,42], *n* = 8 for lead [18,[41], [42], [43], [44], [45], [46], [47]], *n* = 2 for mercury [48,49], and *n* = 4 for PFAS [[50], [51], [52], [53]]) (Table 1). Eleven studies were cross-sectional, and 3 were prospective cohort studies with repeated measures. HM samples were collected from individuals in 14 different states plus Washington DC, mostly on the East and West Coasts (Figure 2) with data collected between 1968 and 2019. Few details were reported on demographic characteristics or nutritional/health status of the individuals who supplied the HM. Some studies described age, BMI, education, income, race/ethnicity, urbanicity, or prior parity or lactation, as shown in Table 1.

**TABLE 1:** Study characteristics (*n* = 15 articles from 14 studies) for studies that assessed arsenic, cadmium, lead, mercury, and per- and polyfluoroalkyl substances (PFAS) concentrations in human milk samples in the United States.

Author, year	Contaminant (s)	Data collection	Sample size and demographics^1^^,^^2^	Background exposure	Milk collection	Milk analytics (critical appraisal)^2^	Funding source
Criswell [50], 2023	PFAS	NH, 2014–2019 (New Hampshire Birth Cohort)	*n* = 392; 32 ± 4.5 y; 54%≥1 child; 42.7% no previous lactation; prepregnancy BMI 55.9% normal, 22.5% overweight, 17.1% obese; 94.4% non-Hispanic White; 71.1% college degree or above	No apparent PFAS pollution in drinking water of rural area	Expressed milk from both breasts, using a standardized protocol with no use of soaps, lotions, or other substances	Liquid chromatography/mass spectrometry (LC−MS/MS); values <limit of quantification (LOQ) were imputed to LOQ/√2.32 (acceptable)	Minnesota CHEAR/HHEAR, National Institute of Environmental Health Sciences, National Institute of General Medical Sciences
Zheng [51], 2021	PFAS	Seattle, WA area, 2019	*N* = 50; 30% <33 y, 68% >33 y; 100% primiparous; 58% normal BMI, 22% overweight, 14% obese; 34% college degree, 62% advanced degree; 16% $45,000–70,000 income, 46% $70,000–100,000, and 36% >$100,000	Not reported (NR)	Manually expressed into polypropylene jars	Ultraperformance liquid chromatograph coupled with a triple-quadrupole mass spectrometer (Agilent 1290 Infinity II ultra performance liquid chromatography (UPLC), 6470 Triple-quadrupole mass spectrometry (QQQ-MS) (acceptable)	National Institute of Environmental Health Science
Klein [41], 2017	Arsenic, cadmium, lead	Boston, MA area, 2013	*n* = 20	NR	Samples were collected between 08:00 and 11:30, and it was requested not within 2 h of the last feed	Perkin Elmer ELAN DRC II inductively coupled plasma—mass spectrometry (ICP-MS) (some concerns)	Harvard University and the National Science Foundation
Carignan [40], 2015	Arsenic	NH, 2012–2013 (New Hampshire Birth Cohort)	*N* = 9; 32 (range = 22–43) y; 1% <11th grade education, 7% high school or General Educational Development (GED), 19% junior college, some college or technical school, 36% college graduate, 37% postgraduate schooling	Home water source as a private, unregulated well	Samples were collected at home into a human milk storage bag or bottle; consumption of expressed milk was mentioned but unknown if this type of milk was tested	ICP-MS (Agilent 7700x; Agilent Technologies) (acceptable)	National Institute of Environmental Health Sciences, NIH, U.S. Environmental Protection Agency, and the University of Arizona
von Ehrenstein [52], 2009	PFAS	Chapel Hill, NC, 2004–2005 (US Environmental Protection Agency Methods Advancement for Milk Analysis Study)	*n* = 20; 31.3 (27.1–34.2) y (median and IQR); all lactation feeding first, second, or third child	NR	Fasted 1.5 h before collection which occurred between 09:00 and 14:00; used a commercially available electric pump (Medela, McHenry, IL)	Reversed-phase HPLC–tandem mass spectrometry (MS/MS). For samples <limit of detection (LOD), values set to LOD/√2. (acceptable)	Eunice Kennedy Shriver National Institute of Child Health and Human Development, National Children’s Study Intra-Agency Coordinating Committee
Tao [53], 2008	PFAS	MA, 2004	*n* = 45; samples from 42 participants; 33 (22–43) y (mean, range); 81% no prior lactation	NR	Expressed all the milk from 1 breast	Agilent 1100 series HPLC coupled with an Applied Biosystems API 2000 electrospray triple-quadrupole mass spectrometer (ESI-MS/MS) (acceptable)	Centers for Disease Control
Sowers [43], 2002	Lead	Camden, NJ, 1997–2000 (Camden Study of Calcium Metabolism in Pregnancy and Lactation)	*n* = 15; 23.7 ± 1.4 y (mean ± SE); 27% nulliparous; 35.5 ± 3.9 mo lactating; prepregnancy body weight 68.4 ± 3.5 kg; 27% Hispanic, 40% African American, 33% White	NR	Breast cleaned with premoistened deionized water wipes. Individual collection kits used for electrically expressed milk whereas using powder-free polyvinyl chloride gloves in a clean environment.	VG PQ II Plus (Thermo Elemental, Franklin, Mass) inductively coupled mass spectrometer with thallium internal standard and 2-point bracketed concentration calibration curve (1–10 parts per billion lead) (acceptable)	NIH
Rabinowitz [44], 1985	Lead	Boston, MA, 1979–1981	*n* = 100 samples collected; 30 y (mean); 87% White; 14.5 y schooling (mean)	NR	Manually expressed into vial without pump or other cup while at home	Anodic stripping voltammetry (acceptable)	National Institute of Child Health and Human Development
Rockway [45], 1984	Lead	Tucson, AZ, NR	*n* = 62 samples; 22–47 y (range)	NR	At least one 8 oz milk sample pooled over multiple days/times	Graphite furnace model 180–70 Hitachi Polarized Zeeman Atomic Absorption Spectrophotometer (acceptable)	NR, acknowledged Leche League for support
Walker Jr. [46], 1980	Lead	Washington, DC, 1978–1979	*n* = 40; NR	NR	Collected in lead-free container	Perkin-Elmer Model 603 atomic absorption spectrophotometer and with the Delves cup technique (some concerns)	NR
Pitkin [48], 1976	Mercury	IA, NR	*n* = 32; NR	Elevated exposure to mercury	Manually expressed from people with lactation of ≥56 d	Atomic absorption spectrophotometry (some concerns)	National Institute of Environmental Health Sciences, National Institute of Arthritis, Metabolism, and Digestive Diseases
Galster [49], 1976	Mercury	Anchorage Bethel, AK, NR	*n* = 21; NR	Lived in Anchorage and Yukon-Kuskokwim Delta which is known to have higher levels of mercury exposure	NR	Flameless atomic absorption after digestion in nitric and sulfuric acid (some concerns)	University of Alaska Sea Grant Program, National Oceanic and Atmospheric Administration, Alaska Public Health and Social Services
Dillon [47], 1974	Lead	NY, KY, CA, TN, 1970–1973	*n* = 29 samples; 23–28 y (range); 100% White, urban, and middle-class	NR	NR	Atomic absorption spectrophotometer (acceptable)	Public Health Service
Lamm [18], 1973	Lead	CT, 1971–1972	*n* = 14 samples; NR	NR	Samples collected in lead-free bottles	Perkin-Elmer Model 305 atomic absorption spectrophotometer with a boat attachment (acceptable)	Connecticut State Department of Consumer Affairs and Connecticut State Department of Agriculture
Murthy [42], 1971	Cadmium, lead	Cincinnati, OH, 1968	*n* = 22 samples, 13 mothers; NR	NR	Collected in glass-stoppered bottle	Atomic absorption spectrophotometer used in these studies was a Jarrel Ash 2 Model 82-360 with HECTO burner (acceptable)	NR

Values reported as means ± SD unless noted otherwise.

Studies are ordered by descending year of publication date.

Abbreviations: CHEAR, Children's Health Exposure Analysis Resource; ESI-MS/MS, electrospray triple-quadrupole mass spectrometer; GED, General Educational Development; HHEAR, Human Health Exposure Analysis Resource; ICP-MS, inductively coupled plasma–mass spectrometry; LC−MS/MS, liquid chromatography/mass spectrometry; LOD, limit of detection; LOQ, limit of quantification; MS/MS, tandem mass spectrometry; NR, not reported; PFAS, per- and polyfluoroalkyl substances; PG II, PlasmaQuad II; UPLC, ultraperformance liquid chromatography.

1 Demographic data that were extracted, if reported, included: age, prior parity, prior lactation, body composition (including bone mineral density and other body composition indicators of nutrient status), socioeconomic variables (race, ethnicity, income, and education), and nutrition or health status (such as anemia/iron status; iron, calcium, vitamin D, and folate intake or supplement use and timing).

2 A technical expert panel with expertise in human biomonitoring and surveillance of environmental toxicants, specifically for heavy metals and PFAS, critically appraised the sample collection and analytic methods used for measuring contaminants as “acceptable” or “some concerns” due to insufficient reporting of methods or quality control.

The type (e.g., colostrum, mature milk) and timing of HM samples collected varied (Table 2), but details were often lacking. Collection of HM across all studies occurred days after delivery [49] to 2 y postpartum [41], with timing not described in 5 studies [18,42,46,47,51]. No information was provided on whether fore- or hindmilk was collected, except 1 study that reported collecting milk in the middle of a feeding [41]. One study reported that milk was collected from both breasts [50], whereas another reported that all milk was expressed from 1 breast [53]. One study reported pooling multiple days of milk into 1 sample [45]. Four studies mentioned HM expression with lactation pumps [43,50,52,53]. The sample collection and analytic methods of 11 studies [18,40,[43], [44], [45],^47^,[50], [51], [52], [53], [54]] were critically appraised by the TEP as “acceptable” and 4 studies [41,46,48,49] had “some concerns” due to insufficient reporting of methods or quality controls (Table 1).

**TABLE 2:** Arsenic, cadmium, lead, and mercury concentrations in human milk samples in the United States (*n* = 11 studies).

Author, year	Collection timing	Arsenic (μg/L, no. samples detected/total samples)^1^	Cadmium (μg/L, no. samples detected/total samples)	Lead (μg/L, no. samples detected/total samples)	Mercury (μg/L, no. samples detected/total samples)^2^
Klein [41], 2017	2 wk to 2 y postpartum	3.47 ± 0.84, NR/20	Not detected, 0/20	0.77 ± 0.45, NR/20	—
Carignan [40], 2015	2–7 wk postpartum	0.33 ± 0.17,^3^ 6/9^4^	—	—	—
Sowers [43], 2002	1.5 mo postpartum	—	—	6.1 ± 1 (SE), NR/15	—
	3 mo postpartum			5.6 ± 1.1 (SE), NR/15	
	6 mo postpartum			5.9 ± 1 (SE), NR/15	
	12 mo postpartum			4.3 ± 1.6 (SE), NR/15	
Rabinowitz [44], 1985	1 mo and 6 mo combined	—	—	17 ± 2 (SE), NR/100	—
Rockway [45], 1984	Composite of 1–22 mo postpartum	—	—	2.8 ± 1.6, 62/62	—
	1–3 mo			2.8, 13/13	
	4–6 mo			2.3, 18/18	
	7–9 mo			3.4, 9/9	
	10–12 mo			3.6, 10/10	
	13–15 mo			3.0, 7/7	
	≥ 16 mo			2.0, 5/5	
Walker Jr. [46], 1980	NR	—	—	20 (range: 0–50), NR/40	—
Pitkin [48], 1976	56+ d postpartum	—	—	—	0.93 ± 0.23 (SE), 14/32
Galster [49], 1976	A few days postpartum Interior area	—	—	—	3.2 ± 0.8 (SE), NR/5
	Urban area				3.3 ± 0.5 (SE), NR/5
	Coastal area				7.6 ± 2.7 (SE), NR/11
Dillon [47], 1974	NR	—	—	26 ± 11, 29/29 (range: 6–58)	—
Lamm [18], 1973	NR	—	—	20, NR/14 (range: 0–70)	—
Murthy [42], 1971	NR	—	19 ± 27, NR/22	12 ± 4, NR/22	—

Values reported as mean ± SD unless noted otherwise.

Studies are ordered by descending year of publication date.

Concentration data were converted, as needed, so that results were reported in the same unit within each contaminant for comparison.

A technical expert panel with expertise in human biomonitoring and surveillance of environmental toxicants, specifically for heavy metals and per- and polyfluoroalkyl substances (PFAS), critically appraised the sample collection and analytic methods used for measuring contaminants as “acceptable” or “some concerns” (Klein [41], Walker Jr. [46], Pitkin [48], and Galster [49] were flagged with “some concerns” due to insufficient reporting of methods or quality control).

Due to inconsistent reporting on the limit of detection, the number of samples detected was reported if available.

Abbreviation: NR, not reported.

1 No studies reported speciation of arsenic.

2 No studies reported speciation of mercury.

3 Mean and SD were calculated from median, range, and sample size using Meta-Analysis Accelerator online conversion calculator [34]. Median and Q1–Q3 reported in Carignan [40] 2015 was 0.31 (0.25–0.44).

4 A table in Carignan [40] reported 6 of 9 samples detected whereas the text reported 5 of 9 samples detected.

#### Arsenic

Arsenic was assessed and detected in HM in 2 cross-sectional studies with samples collected in 2012–2013 [40,41] (Table 1). In 1 study, the mean total arsenic concentration in milk collected mid-feed at 1 time point (anywhere from 2 wk to 2 y postpartum) was 3.47 ± 0.84 μg/L from 20 participants in Boston, Massachusetts [41]. Arsenic speciation was not reported. The TEP critically appraised this study with “some concerns” due to lack of details on method performance (Table 1). In the other study, the mean arsenic concentration of HM collected at 2–7 wk postpartum was 0.33 ± 0.17 μg/L from 9 participants in New Hampshire (critically appraised as “acceptable”) [40]. Arsenic speciation was not investigated due to low concentrations. Both articles were at high ROB due to missing data [41] or selection bias [40] (Table 3).

**TABLE 3:** Risk of bias for studies that assessed arsenic, cadmium, lead, mercury, and per- and polyfluoroalkyl substances (PFAS) concentrations in human milk samples in the United States.

Author, year	Confounding	Exposure measurement	Selection bias	Postexposure intervention	Missing data	Outcome measurement	Reporting bias	Overall
**Arsenic**								
Klein [41], 2017	Low	Low	Low	Low	High	Low	Low	High
Carignan [40], 2015	Low	Low	High	Low	Low	Low	Low	High
Cadmium								
Klein [41], 2017	Low	Low	Low	Low	High	Low	Low	High
Murthy [42], 1971	Low	Low	Some concerns	Low	High	Low	Low	High
**Lead**								
Klein [41], 2017	Low	Low	Low	Low	High	Low	Low	High
Sowers [43], 2002	Low	Low	Some concerns	Low	High	Low	Low	High
Rabinowitz [44], 1985	Low	Low	Some concerns	Low	High	Low	Low	High
Rockway [45], 1984	Low	Low	Some concerns	Low	High	Low	Low	High
Walker Jr. [46], 1980	Low	Low	Some concerns	Low	High	Low	Low	High
Dillon [47], 1974	Low	Low	Some concerns	Low	Some concerns	Low	Low	Some concerns
Lamm [18], 1973	Low	Low	Some concerns	Low	High	Low	Low	High
Murthy [42], 1971	Low	Low	Some concerns	Low	High	Low	Low	High
**Mercury**								
Pitkin [48], 1976	Low	Low	Some concerns	Low	Some concerns	Low	Low	Some concerns
Galster [49], 1976	Low	Low	Some concerns	Low	High	Low	Low	High
**PFAS**								
Criswell [50], 2023	Low	Low	Some concerns	Low	Some concerns	Low	Low	Some concerns
Zheng [51], 2021	Low	Low	Some concerns	Low	Some concerns	Low	Low	Some concerns
Tao [53], 2008	Low	Low	Some concerns	Low	Some concerns	Low	Low	Some concerns
von Ehrenstein [52], 2009	Low	Low	Some concerns	Low	Some concerns	Low	Low	Some concerns

See Supplemental Table 4 for operationalization of ROBINS-E for this research question.

Abbreviation: ROBINS-E, Risk Of Bias In Non-randomized Studies of Exposures.

The evidence did not support a conclusion about the composition and variability of total or inorganic arsenic in HM in the United States. This was due to small sample sizes from 1 geographical region and little detail on demographic characteristics to determine generalizability to the United States.

#### Cadmium

Cadmium was assessed in HM in 2 cross-sectional studies with samples collected in 1968 [42] and 2013 [41] (Table 1). The 1968 study detected a mean cadmium concentration of 19 ± 27 μg/L in 22 samples from 13 participants from Cincinnati (critically appraised as “acceptable”) [42]. The 2017 study did not detect cadmium in HM from 20 participants in Boston [41]. The analytic methods of this study had “some concerns” due to insufficient reporting about procedures [41]. Both articles were at high ROB due to concerns of missing data (Table 3).

The evidence did not support a conclusion about the composition and variability of cadmium in HM in the United States. This was due to small sample sizes from 1 geographical region with little detail on demographic characteristics to determine generalizability to the United States. Additionally, 1 of the 2 articles was published > 50 y ago [42].

#### Lead

Lead was assessed and detected in HM in 2 prospective cohorts [43,45] (both critically appraised as “acceptable”) and 6 cross-sectional studies [18,41,42,44,46,47] (4 “acceptable” [18,42,44,47] and 2 with “some concerns” [41,46]) with samples collected between 1968 and 2013 in 9 different states of United States, plus Washington, DC (Table 1). Across all studies, mean lead concentrations ranged from 0.77 ± 0.45 [41] μg/L collected in 2013 to 26 ± 11 [47] μg/L collected in 1970–1973 (Table 2). The former study was the only one that collected data within the last 25 y and had the lowest mean lead concentration among all studies [41]. However, it was critically appraised with “some concerns” due to insufficient reporting of method performance. In the 2 prospective cohort studies, repeated measures were taken over time to assess changes in lead concentrations in HM. There were no statistically significant changes in lead from 1.5, to 3, to 6, to 12 mo in 1 study [43] or from 1–3, to 4–6, to 7–9, to 10–12, to 13–15 to ≥16 mo in the other [45]. Overall, 1 study had some concerns for bias due to missing data and selection bias [47], whereas the remaining were at high ROB due to missing data (Table 3).

The evidence did not support a conclusion about the composition and variability of lead in HM in the United States as only 2 of 8 studies collected data within the past 25 y [41,43]. Therefore, the evidence is likely not reflective of current exposure during pregnancy and lactation in the United States.

#### Mercury

Mercury was assessed and detected in HM in 2 cross-sectional studies with samples collected before 1976 (exact dates not reported; Table 1) [48,49]. The mean total mercury concentration of HM was 0.93 ± 0.23 μg/L from 32 participants in Iowa at 56+ days postpartum [48]. In 11 participants from Alaska, mean total mercury concentrations in HM collected a few days postpartum ranged from 3.2 ± 0.8 to 7.6 ± 2.7 μg/L; participants from coastal areas had a 2-fold higher mercury concentration in their HM samples compared with those living in a more interior region (Table 2) [49]. Both studies were critically appraised with “some concerns” about the analytic methods due to insufficient reporting. One study was at high ROB [49], whereas 1 had some concerns [48], largely due to selection bias and missing data (Table 3). No studies assessed methylmercury.

The evidence did not support a conclusion about the composition and variability of total mercury or methylmercury in HM in the United States. There were concerns about the sample collection and analytic methods, and the data were collected almost 50 y ago.

#### Per- and polyfluoroalkyl substances

PFAS were assessed in HM in 3 cross-sectional studies [50,51,53] and 1 prospective cohort [52] with samples collected from 2004 to 2019 (Table 1). Samples of HM (*n* = 559) were collected from 5.5 wk to 17 mo postpartum from ∼540 participants in different parts of the United States (Table 4). All studies were critically appraised by the TEP as “acceptable.”

**TABLE 4:** Per- and polyfluoroalkyl substances (PFAS) concentrations in human milk samples in the United States (*n* = 4 studies).

Author, year	Milk type or timing	Perfluorooctanoic acid (PFOA) (pg/mL, no. samples detected/total samples)	Perfluorooctane sulfonate (PFOS) (pg/mL, no. samples detected/total samples)	Perfluorononanoic acid (PFNA) (pg/mL, no. samples detected/total samples)	Perfluorohexane sulfonate (PFHxS) (pg/mL, no. samples detected/total samples)
Criswell [50], 2023^1^	Mature milk 6 wk postpartum	17 (12–27), 356/426	24 (16–36), 392/426	Not detected, 29/426	Not detected, 32/426
Zheng [51], 2021^2^	NR	13.9 (10.6–25.3), 43/50^3^	30.4 (16.8–63.0), 50/50	5.98 (4.13–8.40), 50/50	6.55 (5.25–7.45), 45/50^3^
von Ehrenstein [52], 2009	5.5 wk postpartum	Not detected, 0/18	Not detected, 0/18	Not detected, 0/18	Not detected, 0/18
	13 wk postpartum	Not detected, 0/20	Not detected, 0/20	Not detected, 0/20	Not detected, 0/20
Tao [53], 2008	1–17 mo postpartum	36.1 (<30.1–161), 40/45	106 (<32–617), 43/45	6.97 (<5.2–18.4), 29/45	12.1 (<12–63.8), 23/45

Values reported as median (Q1–Q3) except Tao [53], which reported median (range).

Studies are ordered by descending year of publication date.

All but 1 study reported results in pg/mL, so data were converted [55] for comparison.

A technical expert panel with expertise in human biomonitoring and surveillance of environmental toxicants, specifically for heavy metals and PFAS, critically appraised the sample collection and analytic methods used for measuring contaminants as “acceptable.”

1 Values converted from ng/mL; ng/mL∗1000 = pg/mL.

2 Zheng, 2021 [51] also reported a median and Q1–Q3 of 121 (103–190) pg/mL for total PFAS.

3 Detection values were calculated.

PFOA and PFOS were detected in 439 of 559 and 485 of 559 HM samples, respectively, in 3 [50,51,53] of 4 [52] studies. Median PFOA concentrations ranged from 13.9 [51] to 36.1 [53] pg/mL and median PFOS concentrations ranged from 24 [50] to 106 [53] pg/mL. PFOA and PFOS were not detected in the prospective study that collected milk samples at both 5.5 and 13 wk postpartum from ∼20 individuals in the southeast [52]. PFNA and PFHxS were detected in only 2 [51,53] of the 4 [50,52] studies in 79 of 559 and 68 of 559 HM samples. All studies had some concerns of bias mainly due to selection bias and missing data (Table 3) [[50], [51], [52], [53]].

This evidence suggested that median PFOA concentrations in HM in the United States ranged from undetected to 36.1 pg/mL and that median PFOS concentrations in HM ranged from undetected to 106 pg/mL. The certainty of both conclusions was moderate, downgraded due to concerns about ROB (Table 5). Sensitivity analyses were not conducted because there were no articles at high ROB. Results for PFNA and PFHxS were inconsistent in the detection frequency, and thus, the evidence did not support a conclusion.

**TABLE 5:** Certainty of evidence^1^ for conclusions about perfluorooctanoic acid (PFOA) and perfluorooctane sulfonate (PFOS) concentrations in human milk samples in the United States.

No. of articles	Risk of bias^2^	Inconsistency^3^	Indirectness^3^	Imprecision^3^	Publication bias^4^
The available evidence suggested that median PFOA concentration in human milk ranged from undetected to 36.1 pg/mL (certainty: moderate).					

4 Criswell [50], 2023 Zheng [51], 2021 von Ehrenstein [52], 2009 Tao [53], 2008	Serious	Not serious	Not serious	Not serious	Undetected

The available evidence suggested that median PFOS concentration in human milk ranged from undetected to 106 pg/mL (certainty: moderate).					
4 Criswell [50], 2023 Zheng [51], 2021 von Ehrenstein [52], 2009 Tao [53], 2008	Serious	Not serious	Not serious	Not serious	Undetected

Abbreviation: GRADE, Grading of Recommendations, Assessment, Development, and Evaluation.

All data were descriptive (from nonrandomized studies of exposures [39], i.e., 3 cross-sectional studies and 1 prospective cohort). Thus, all study designs were assessed together and upgrading for residual confounding, magnitude of effect, and dose–response were not considered.

1 GRADE rating: very low, low, moderate, or high certainty.

2 Domain only downgrades. Rating choices: extremely serious, very serious, serious, or not serious. Both conclusions were downgraded once because all articles had at least “some concerns” due to selection bias and missing data.

3 Domain only downgrades. Rating choices: very serious, serious, or not serious. Neither conclusion was downgraded in these domains.

4 Domain only downgrades. Rating choices: strongly detected or undetected. Neither conclusion was downgraded in this domain.

#### Heat maps for countries that are “high” or “very high” HDI

There were 332 articles that assessed contaminants in HM from 55 countries that were “high” or “very high” on the HDI, including the 15 articles from the United States. Lead concentrations in HM were the most frequently assessed (*n* = 171 articles), followed by cadmium (*n* = 139), mercury (*n* = 124), arsenic (*n* = 87), and PFAS (*n* = 85) (Supplemental Figure 2 and Supplemental Table 7). Iran, Spain, and China had the most published evidence on concentrations of these contaminants in HM.

### Contaminants assessed in IF

#### Search results

**FIGURE 3:**
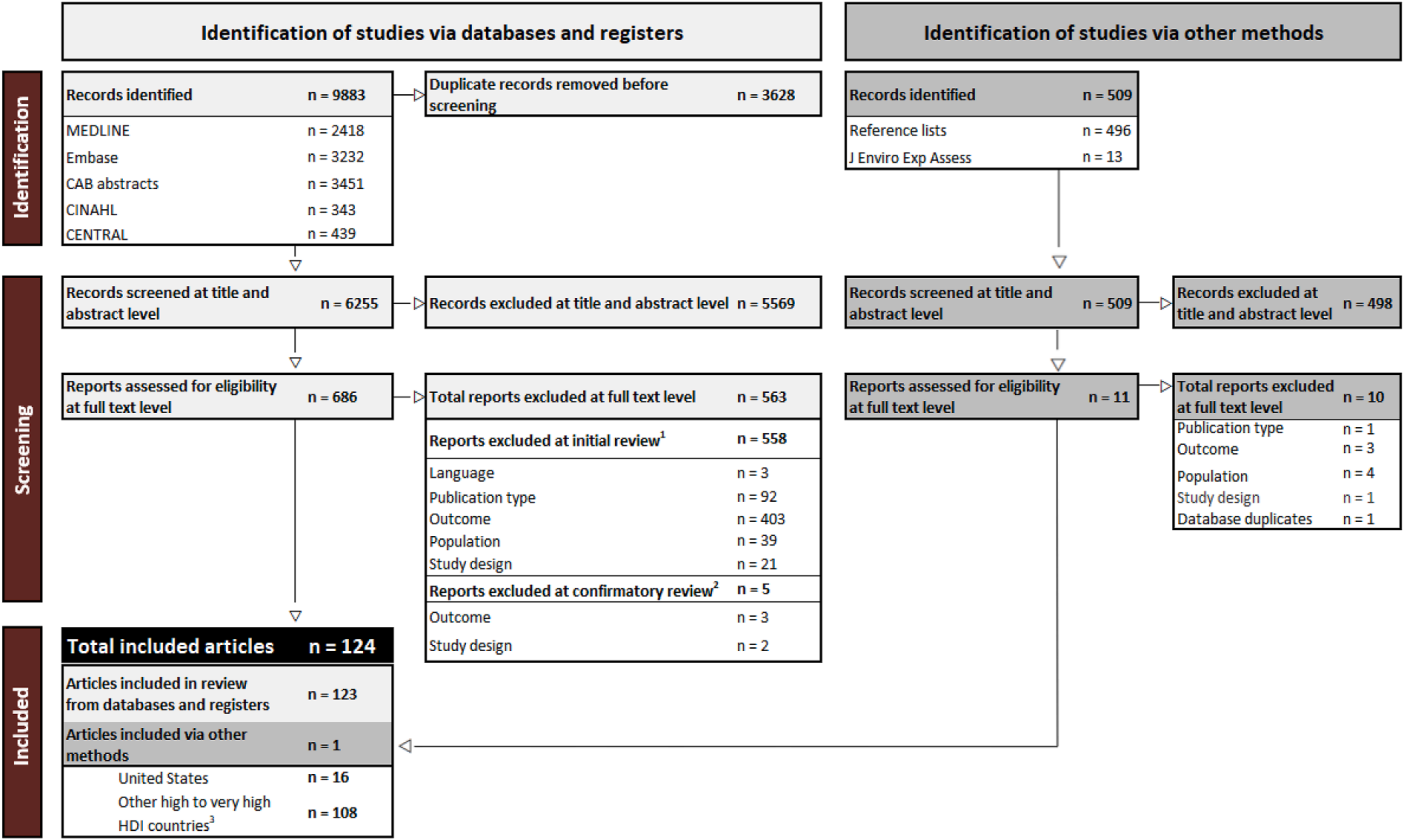
PRISMA diagram for studies that assessed arsenic, cadmium, lead, mercury, and per- and polyfluoroalkyl substances (PFAS) concentrations in infant formula products. ^1^During initial review, data related to the contaminant assessed, whether the contaminant was assessed in human milk, infant formula, and/or infant biospecimens, and the country of data collection were extracted. ^2^The extracted data helped identify articles for inclusion in the 5 systematic review questions. Using Stata (Stata Corp), the articles were separated into datasets specific to each question and were further screenedto confirm that all inclusion and exclusion criteria were met.^3^ Non-United States countries rated “high” and “very high” in the Human Development Index (HDI) are only included in the heat maps. The HDI is a measurement of development including: quality of life, education, and quality of living [29]. Database details: CAB Abstracts (Ovid), and CENTRAL (Cochrane Library), CINAHL (EBSCO), Embase (Ovid), Medline (Ovid). J Enviro Exp Assess, *Journal of Environmental Exposure Assessment*; PRISMA, Preferred Reporting Items for Systematic Reviews and Meta-Analyses.

The database search identified 6255 unique records that were screened at title/abstract level (Figure 3). During initial full-text screening, 558 of 686 full-text articles were excluded because they were not relevant to IF. The remaining 128 articles underwent a second full-text screening to confirm eligibility. Five articles were excluded at this confirmatory level due to outcome and study design (Supplemental Table 8). One additional article identified from the *Journal of Environmental Exposure Assessment* was included.

#### Contaminants assessed in IF in the United States

**FIGURE 4:**
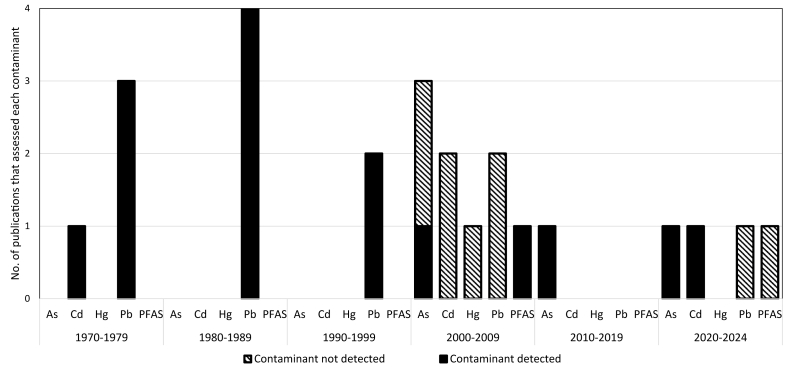
Arsenic (As), cadmium (Cd), lead (Pb), mercury (Hg), and per- and polyfluoroalkyl substances (PFAS) assessed and detected over time in infant formula products analyzed in United States studies.

We identified 16 United States studies that assessed arsenic (*n* = 5) [[56], [57], [58], [59], [60]], cadmium (*n* = 4) [42,56,58,60], lead (*n* = 12) [18,42,44,46,54,56,58,[60], [61], [62], [63], [64]], mercury (*n* = 1) [60], and/or PFAS (*n* = 2) [65,66]. All study designs were cross-sectional, except for 2 prospective cohort studies [[56], [57], [58], [59], [60],^65^,^66^]. The year of sample collection was often not reported, but the earliest reported was 1968, and the most recent was 2023. Therefore, we rely on publication dates to describe the studies, which ranged from 1971 to 2024 (Figure 4). The 7 studies published between 2002 and 2024 (Table 6) [18,42,44,46,54,[61], [62], [63], [64]] included assessments of all contaminants. The 9 studies published between 1971 and 1997 (Supplemental Table 9) [44,64] only included cadmium and lead assessments (Supplemental Table 10). The TEP critically appraised all studies as “acceptable,” except for 1 published in 1980 that lacked information on quality controls for lead assessment [46].

**TABLE 6:** Study characteristics (*n* = 7 studies) for studies that assessed arsenic, cadmium, lead, mercury, and per- and polyfluoroalkyl substances (PFAS) concentrations in infant formula products in the United States published between 2000 and 2024.

Author, year	Contaminant(s)	Data collection	Infant formula sampling strategy^1^	Analytic methods (critical appraisal)^2^	Funding source
Bogdan [65], 2024	PFAS	Minnesota (NR) (purchasing data to inform sampling protocol from December 2021 to January 2023)	17 different products; 10 based on WIC purchasing statistics for the most popular contract and medical formulas	Liquid chromatography tandem-mass spectrometry (Shimadzu Prominence, AB Sciex 5500Q) (Acceptable)	Clean Water Fund from the 2008 Minnesota Clean Water, Land, and Legacy Amendment
TatahMentan [56], 2024	Arsenic Cadmium Lead	Products consumed in the United States that were purchased online (NR)	NR	Inductively coupled plasma–mass spectrometry (Agilent 7900 ICP-MS) (Acceptable)	National Institute on Minority Health and Health Disparities, Tulane University’s Carol Lavin Bernick Faculty Grant
Jackson [57], 2012	Arsenic	Hanover, NH (NR)	Purchased 15 different products from local supermarkets from 5 main brands.	ICP-MS (7700×, Agilent, Santa Clara, CA); speciation analysis was by anion-exchange chromatography coupled to ICP-MS. (Acceptable)	National Institute of Environmental Health Sciences, NIH, and Environmental Protection Agency
Tao [66], 2008	PFAS	Washington, DC; Boston, MA (2007)	21 different products from 5 manufacturers representing >99% of the United States market, purchased from local stores.	HPLC (Agilent 1100 Series) coupled with an API 2000 electrospray triple-quadrupole mass spectrometer (ESI-MS/MS, Applied Biosystems; Foster City, CA). (Acceptable)	Japan Society for the Promotion of Science, Ministry of Environment of Japan, Japanese Ministry of Education, Culture, Sports, Science and Technology
Olu-Owolabi [58], 2007	Arsenic Cadmium Lead	Multiple cities across the United States but not described (2003–2004)	Purchased at various times of the year from different supermarkets or grocery stores in different cities to allow for randomness, ≥5 samples of each brand collected; only 1 brand purchased for the United States.	Wet oxidation method and radial inductive coupled plasma emission spectrometry (Perkin Elmer, OPTIMA 3000). (Acceptable)	NR
Vela [59], 2004	Arsenic	Cincinnati, OH (NR)	Purchased 4 products from a local supermarket.	ICP-MS Model PQ3 (Thermo Elemental, Franklin, MA) for total and speciated arsenic determinations. (Acceptable)	US Environmental Protection Agency
Ikem [60], 2002	Arsenic Cadmium Lead Mercury	Auburn, AL (2000)	Purchased 5 different brands from different supermarkets across major supermarket chains at various times of the year allows for randomness.	A Perkin-Elmer DV 3300 inductively coupled plasma–optical emission spectrophotometer (ICP–OES). (Acceptable)	National Aeronautics and Space Administration, US Department of Energy

Studies are ordered by descending year of publication date.

Data extracted from studies published before 2000 are shown in Supplemental Table 9.

Abbreviations: ESI-MS/MS, electrospray triple-quadrupole mass spectrometer; ICP-MS, inductively coupled plasma–mass spectrometry; ICP–OES, inductively coupled plasma–optical emission spectrophotometer; NR, not reported; PFAS, per- and polyfluoroalkyl substances; WIC, Special Supplemental Nutrition Program for Women, Infants, and Children.

1 All studies were cross-sectional and descriptive assessments of infant formula.

2 A technical expert panel with expertise in human biomonitoring and surveillance of environmental toxicants, specifically for heavy metals and per- and polyfluoroalkyl substances (PFAS), critically appraised the sample collection and analytic methods used for measuring contaminants as “acceptable” or “some concerns” due to insufficient reporting of methods or quality control.

For ROB, all studies either had some concerns or were at high risk (Table 7). The outcome assessment domain had some concerns for all studies due to a lack of reporting on blinding during sample preparation and processing. For the sampling strategy domain, 2 studies were at low ROB because the product selection was informed by market data to obtain a representative sample of IF [65,66]. Three studies had some concerns because they did not use market data to inform sampling, but did consider location and time of year [42,58,60]. The remaining studies were at high ROB for not considering or reporting details on the types, brands, and quantity of samples tested, which also contributed to ambiguity in the sampling scheme.

**TABLE 7:** Risk of bias for studies that assessed arsenic, cadmium, lead, mercury, and per- and polyfluoroalkyl substances (PFAS) concentrations in infant formula products in the United States.

Author, year	Sampling strategy	Missing data	Outcome assessment	Reporting	Overall
**Arsenic**					
TatahMentan [56], 2024	High	Low	Some concerns	Low	High
Jackson [57], 2012	High	Low	Some concerns	Low	High
Olu-Owolabi [58], 2007	Some concerns	Low	Some concerns	Low	Some concerns
Vela [59], 2004	High	Low	Some concerns	Low	High
Ikem [60], 2002	Some concerns	Low	Some concerns	Low	Some concerns
**Cadmium**					
TatahMentan [56], 2024	High	Low	Some concerns	Low	High
Olu-Owolabi [58], 2007	Some concerns	Low	Some concerns	Low	Some concerns
Ikem [60], 2002	Some concerns	Low	Some concerns	Low	Some concerns
Murthy [42], 1971	High	Low	Some concerns	Low	High
**Lead**					
TatahMentan [56], 2024	High	Low	Some concerns	Low	High
Olu-Owolabi [58], 2007	Some concerns	Low	Some concerns	Low	Some concerns
Ikem [60], 2002	Some concerns	Low	Some concerns	Low	Some concerns
Baum [61], 1997	High	Low	Some concerns	Low	High
Thompson [62], 1993	High	Some concerns	Some concerns	Low	High
Rabinowitz [44], 1985	High	Some concerns	Some concerns	Low	High
Ryu [64], 1983	High	Low	Some concerns	Low	High
Munro [63], 1987	High	Some concerns	Some concerns	Some concerns	High
Walker Jr. [46], 1980	High	Low	Some concerns	Low	High
Lamm [54], 1974	High	Low	Some concerns	Low	High
Lamm [18], 1973	High	Some concerns	Some concerns	Low	High
Murthy [42], 1971	Some concerns	Low	Some concerns	Low	Some concerns
**Mercury**					
Ikem [60], 2002	Some concerns	Low	Some concerns	Low	Some concerns
**PFAS**					
Bogdan [65], 2024	Low	Low	Some concerns	Low	Some concerns
Tao [66], 2008	Low	Some concerns	Some concerns	Low	Some concerns

See Supplemental Table 5 for operationalization of domains.

#### Arsenic

Arsenic was assessed in 5 studies (all critically appraised as “acceptable”) and detected in 3 studies (Table 8) [[56], [57], [58], [59], [60]] published between 2002 and 2024. All products (*n* = 27 although product overlap could not be determined due to inadequate reporting of brand and product names) were powdered, except in 1 study that did not specify the form [57], and were not diluted before sample processing. Among 14 dairy-based products assessed, arsenic was detected in 11 products (*n* = 9, ranging from 2.6 ± 0.44 to 9.38 ± 0.31 (mean ± SD) ng/g in 1 study [57]; and *n* = 2, ranging from 11 to 20 ng/g in another study [56]). Of these dairy-based IF, arsenic was detected in all 4 that reported containing rice [*n* = 2, ranging from 11 to 20 ng/g from 1 study [56]; *n* = 2, 6.02 ± 0.26 to 8.19 ± 0.63 (mean ± SD) ng/g from another study [57]]. Arsenic was also detected in the single nondairy IF with rice 11.89 ± 0.64 (mean ± SD) ng/g [57]. Arsenic was not detected in any of the 3 soy-based products [58,60]. Only 1 of these studies assessed speciated arsenic in samples that had total arsenic concentrations of > 6 ng/g (9 of 15 products); 100% of the measured arsenic was inorganic arsenic [57].

**TABLE 8:** Arsenic, cadmium, lead, mercury, and per- and polyfluoroalkyl substances (PFAS) contaminants in infant formula in the United States (*n* = 7 studies) for studies published between 2002 and 2024.

Author, year	Sample preparation method before analysis (original sample form; dilution source and volume/ratio)	Infant formulas sampled	Arsenic^1^ (ng/g, no. detected/total)	Cadmium (ng/g, no. detected/total)	Lead (no. detected/total)	Mercury^2^ (no. detected/total)	PFAS (pg/mL, no. detected/total)
Bogdan [65], 2024	Form: powdered; dilution: ultrapure water at 20% weight/volume	Dairy	—	—	—	—	PFOA: ND, 0/12 products PFOS: ND,^3^ 1/12 products PFNA: ND, 0/12 products PFHxS: ND, 0/12 products
		Soy	—	—	—	—	PFOA: ND, 0/4 products PFOS: ND, 0/4 products PFNA: ND, 0/4 products PFHxS: ND, 0/4 products
		Amino acid, nondairy	—	—	—	—	PFOA: ND, 0/1 products PFOS: ND, 0/1 products PFNA: ND, 0/1 products PFHxS: ND, 0/1 products
TatahMentan [56], 2024^4^	Form: powdered; dilution: no	Milk-based, with rice starch, iron	11–20 (range), 2/2 samples	2–3 (range), 2/2 samples	ND, 0/2 samples	—	—
Jackson [57], 2012^5^	Form: NR; dilution: no	Dairy, no rice	2.6 ± 0.44 to 9.38 ± 0.31,^6^ 7/7 products (inorganic)	—	—	—	—
		Dairy with rice	6.02 ± 0.26 to 8.19 ± 0.63,^6^ 2/2 products (inorganic)				
		No dairy, no rice	6.95 ± 0.43 to 11.43 ± 1.09,^6^ 5/5 products (inorganic)				
		No dairy with rice	11.89 ± 0.64, 1/1products (inorganic)				
Tao [66], 2008^7^	Form: powdered; dilution: Milli-Q water with unknown volume/ratio Form: concentrated liquid; Dilution: 1:1 with Milli-Q water Form: ready-to-feed; dilution: no	Largely milk- or soy-based from plastic, glass, cardboard, or metal containers, including organic and nonorganic types	—	—	—	—	PFOA: ND, 0/2 samples PFOS: <11 to 11.3, 1/2 samples PFNA: ND, 0/21samples PFHxS: 1.35 to 3.59, 2/21samples (range)
Olu-Owolabi [58], 2007	Form: powdered; dilution: no	Soy + iron in glass bottle	ND, 0/1 products	ND, 0/1 products	ND, 0/1 products	—	—
Vela [59], 2004	Form: powdered (dry weight); dilution: no	Iron fortified	12 ± 2 to 17 ± 3,^6^ 3/3 samples	—	—	—	—
		Low iron	16 ± 1, 1/1samples				
Ikem [60], 2002	Form: powdered; dilution: no^8^	Milk-based, iron fortified in metal container or milk-based, iron fortified hypoallergenic in paper container	ND, 0/3 samples	ND, 0/3 samples	ND, 0/3 samples	ND, 0/3 samples	—
		Soy-based, iron fortified in either metal or paper container	ND, 0/2 samples	ND, 0/2 samples	ND, 0/2 samples	ND, 0/2 samples	

Values reported as mean ± SD unless noted otherwise.

Studies are ordered by descending year of publication date.

Concentration data were converted, as needed, so that results were reported in the same unit within each contaminant for comparison.

A technical expert panel with expertise in human biomonitoring and surveillance of environmental toxicants, specifically for heavy metals and per- and polyfluoroalkyl substances (PFAS), critically appraised the sample collection and analytic methods used for measuring contaminants as “acceptable.” Results of studies published before 2000 are shown in Supplemental Table 10.

There may be overlap among the infant formula products sampled, which cannot be determined due to a lack of reported descriptions. More details about the infant formulas, including brand names, if available, are available in Supplemental Table 12.

Abbreviations: ND, not detected; ng/g, nanogram per gram; PFHxS, perfluorohexane sulfonate; PFNA, perfluorononanoic acid; PFOA, perfluorooctanoic acid; PFOS, perfluorooctane sulfonate.

1 Only 1 study, Jackson, 2012 [57], reported speciation of arsenic.

2 No studies reported speciation of mercury.

3 PFOS was detected in 1 dairy-based product (brand not specified): mean ± SD was 0.054 ± 0.003 ng/g analyzed in powder form.

4 Values were extracted from the supplement file.

5 Measured speciated arsenic in IF samples that had total arsenic concentrations of >6 ng/g (9 of 15 products); 100% of the measured arsenic was inorganic arsenic.

6 Reported as a range of sample means ± SD for the 3 IF tested.

7 Mean and median values were not reported; mean and median values were calculated only if >70% of the samples had concentrations above the limit of quantitation (LOQ). Sample detection was calculated.

8 Preparation method not explicitly stated, but did not state that the powdered infant formula was diluted or that water was added.

Overall, detection was inconsistent, sample size was small, sampling schemes were inadequate, and brand or manufacturer information was lacking, which limited the ability to determine if the 27 products were unique from one another. Arsenic was inconsistently detected across studies, and it was unclear if the 27 products assessed were unique because the IF type was not adequately described. Therefore, the evidence did not support a conclusion on the composition and variability of total or inorganic arsenic in IF in the United States. Although we could not draw conclusions, all reported concentrations ranged between 2.6 and 20 ng/g which is at or below the European Union maximum guideline for powdered formula (0.02 mg/kg or 20 ng/g) [67].

#### Cadmium

Cadmium was assessed in 3 studies [56,58,60] (all were critically appraised as “acceptable”) published between 2002 and 2024. All IF (*n* = 8 products) were powdered and not diluted before sample processing. Cadmium was detected in 1 study that assessed 2 milk-based products with rice starch and iron ranging from 2 to 3 ng/g [56] but was not detected in the other 2 studies assessing soy- and milk-based IF with iron fortification [58,60] (Table 8).

Cadmium was also assessed in a study published in 1971 (Supplemental Table 9) [42] and was detected in all 4 IF that were diluted 1:1 (original form, water source, and dilution timing not reported). Concentrations ranged from 20 ± 4 to 39 ± 7 (mean ± SD) ng/g with higher values for soy-based compared with dairy-based products (Supplemental Table 10) [42].

Overall, results were inconsistent, sample preparation varied, sample size was small, sampling schemes were inadequate, and brand or manufacturer information was lacking, which limited the ability to determine if the 12 products were unique from one another. Therefore, the evidence did not support a conclusion for the composition and variability of cadmium in IF in the United States.

#### Lead

Lead was assessed but not detected in 3 studies [56,58,60] (all critically appraised as “acceptable”) published between 2002 and 2024. The products (*n* = 8) were powdered and not diluted before sample processing. Products were described as soy-based with iron (*n* = 3) or milk-based with iron (*n* = 5; 2 included rice starch) (Table 8).

Lead was assessed and detected in 9 studies [18,42,44,46,54,[61], [62], [63], [64]] (all were critically appraised as “acceptable” except 1 that had “some concerns” [46]) published between 1971 and 1997. Lead was detected in IF regardless of variations in the protein-based, preparation method, or water source (Supplemental Table 10). The lowest mean lead concentration was 0.24 ± 0.06 ng/g in a milk-based IF (original formula form was concentrate, dilution information was not reported) [62], and the highest mean was 540 ± 110 ng/g in a soy-based product (original formula form not reported, diluted 1:1 at an unknown timepoint with unknown water source) [42].

Overall, most of the data were from before 1997, whereas studies published between 2002 and 2024 had small sample sizes and lacked brand and manufacturing information. Thus, the evidence did not support a conclusion on the composition and variability of lead in IF in the United States.

#### Mercury

Mercury was assessed but not detected in 1 study published in 2002 (critically appraised as “acceptable”) (Table 6) [60]. Products tested (*n* = 5) included powdered and undiluted milk- or soy-based IF (Table 8) [60].

Results from a single study with a small sample size are not representative of the United States market and did not support a conclusion on the composition and variability of mercury in IF in the United States.

#### Per- and polyfluoroalkyl substances

PFAS were assessed in 2 studies published in 2008 [66] and 2024 [65] (both critically appraised as “acceptable”; Table 6) that collectively assessed 38 IF products. The study from 2008 [66] assessed 21 products, largely milk- or soy-based, including organic and nonorganic, with brands representing 99% of the United States market [66]. Powdered and concentrated liquid IF were diluted with Milli-Q water (powdered dilution not reported; concentrated liquid diluted 1:1); ready-to-eat IF was not diluted before sample processing. The study from 2024 [65] assessed 17 different powdered products with a sampling scheme largely based on Minnesota purchasing data with 10 products from the Women, Infants, and Children program purchases. These included dairy, soy, nondairy amino acid hypoallergenic blends, medical formulas, organic, non-genetically modified organism, and with and without rice starch. All were diluted with ultrapure water at 20% weight/volume. In these 2 studies (Table 8), PFOA and PFNA were not detected in any IF. PFOS and PFHxS were detected in ≤10% [65] and ≤30% [66] of samples.

Overall, the evidence suggested that PFAS were largely not detected in IF available in the United States. This was supported by a total of 38 IF (≥17 unique products) assessed that included a variety of IF available in the United States between 2008 and 2024 identified via comprehensive sampling strategies based on purchasing data. The certainty of this conclusion was moderate due to concerns of publication bias (Table 9). Sensitivity analyses were not conducted because there were no articles at high ROB.

**TABLE 9:** Certainty of evidence^1^ for conclusions about per- and polyfluoroalkyl substances (PFAS) concentrations in infant formula products in the United States.

No. of articles	Risk of bias^2^	Inconsistency^3^	Indirectness^3^	Imprecision^3^	Publication bias^4^
The available evidence suggested that PFAS are largely not detected in infant formula assessed in studies from the United States. (certainty: moderate^1^).					

2 Bogdan [65], 2024 Tao [66], 2008	Not serious	Not serious	Not serious	Not serious	Strongly detected

All data were descriptive (from nonrandomized studies of exposures [39], i.e., cross-sectional studies). Thus, upgrading for residual confounding, magnitude of effect, and dose–response were not considered.

Abbreviation: GRADE, Grading of Recommendations, Assessment, Development, and Evaluation.

1 GRADE rating: very low, low, moderate, or high certainty.

2 Domain only downgrades. Rating choices: extremely serious, very serious, serious, or not serious. The conclusion was not downgraded in this domain.

3 Domain only downgrades. Rating choices: very serious, serious, or not serious. This conclusion was not downgraded in these domains.

4 Domain only downgrades. Rating choices: strongly detected or undetected. This conclusion was downgraded for high risk of publication bias because industry or regulatory data are routinely collected but not published in peer-reviewed literature.

#### Heat maps for countries that are “high” or “very high” HDI

There were 124 articles about contaminants in IF from countries rated “high” or “very high” on the HDI [29], including the 16 United States studies. Lead in IF was most frequently assessed (*n* = 119 articles), followed by cadmium (*n* = 95), arsenic (*n* = 55), mercury (*n* = 36), and PFAS (*n* = 22). Spain, United States, and Poland had the most published evidence on concentrations of these contaminants in IF (Supplemental Figure 3 and Supplemental Table 11).

## Discussion

Infants rely on HM and IF as primary food sources to support healthy development. It is crucial to understand potential environmental contaminant exposure from these foods. Our systematic review found a paucity of evidence about arsenic, cadmium, lead, and mercury concentrations in HM and IF in the United States which precluded our ability to draw conclusions. For PFAS, we found that PFOA and PFOS were detected in HM in the United States which aligns with findings from other countries [68] and is likely a reflection of lifelong ubiquitous PFAS exposure. Higher exposure to PFOA and PFOS, as well as other PFAS chemicals, is associated with a higher risk of pregnancy complications, low birth weight, alterations in sex hormones, infertility, DNA damage [69,70], allergies, susceptibility to infectious diseases, immunosuppression [70,71], and autoimmune diseases [71]. However, the science is still emerging on this topic. Associations with these health outcomes depend on dose, frequency, and duration of exposure as well as individual factors such as baseline health status and other social determinants of health [72]. Also, these associations are from observational studies so research into causal mechanisms is needed. Lastly, we found that PFAS was undetected in the majority of IF products assessed in the United States studies.

The presence of PFAS in HM and not IF may reflect the high regulatory control of IF in the United States, managed by ingredient sourcing, processing, and manufacturing. Most data in this systematic review represent contaminant concentrations in IF prepared in a laboratory setting, often undiluted, and do not necessarily reflect contaminant concentrations in IF when prepared at home and consumed by infants. Additionally, PFAS (and other contaminants) can be introduced into IF during preparation using home water sources [2]. Therefore, the contaminant concentrations in HM and IF reported in this review should not be directly compared because HM is an unregulated whole food that does not require further preparation. Overall, public health organizations worldwide recommend HM as the best source of nutrition for most infants [4,73]. Monitoring and surveillance of environmental contaminant exposure during pregnancy and lactation are needed in the United States to limit transfer of potential contaminants into HM and mitigate infant exposure [74,75].

The United States Environmental Protection Agency issued concerns about PFAS exposure in the late 1990s. PFAS are human-made chemicals often referred to as “forever chemicals” because they are widely used and break down slowly over time [76]. People are exposed to PFAS via food, water, consumer goods, and dust across the lifespan, including during pregnancy and lactation [77]. There is likely a combination of long- and short-term exposures due to pervasive PFAS use and limited degradation over time [78]. A previous systematic review indicated that PFAS can be excreted into HM, likely by binding to various proteins [68]. Additionally, PFAS can be introduced to HM when not fed directly from the breast through potential contamination from storage containers, feeding bottles, cleansing products, and breast pumps, as these can be sources of PFAS [79]. Our results build upon this research by describing the median concentrations of PFAS in HM in the United States from peer-reviewed studies, which was undetected ≤36.1 pg/mL for PFOA and undetected ≤106 pg/mL for PFOS. However, there are no established reference values for PFAS in HM to contextualize these results, emphasizing a need for future research. Eliminating PFAS in HM is not a realistic goal due to the ubiquity of PFAS in the environment, although efforts can be made to reduce PFAS in foods over time [25]. The United States Centers for Disease Control and Prevention states that “current science suggests the benefits of breastfeeding or providing human milk outweigh potential risks of PFAS” [80] exposure.

Timely and representative data on arsenic, cadmium, lead, and mercury in HM in the United States are lacking. Of 11, 8 included studies were published in the 1970–1980s. Heavy metal exposure has decreased since then, especially lead [[81], [82], [83]], limiting the utility of older data to reflect current exposure [[84], [85], [86], [87], [88], [89]]. Furthermore, most of the included studies reported results derived from small samples that were not representative of the United States population. Contaminant exposure amount and source can differ based on geographical location (e.g., proximity to industrial sites [90] or urban areas [91]), occupations (e.g., construction [92] or mining [93]), as well as environmental inequalities (e.g., disproportional exposure to environmental contaminants largely via air pollution [94,95]). Most studies in our review were conducted on the East or West Coast, emphasizing the need for more representative data collection, including in the middle of the United States, where data are lacking and exposures may differ from coastal regions. Additionally, future research should include more detailed demographic and biological information on the lactating individual, including nutritional status, dietary intake, drinking water source as well as HM collection methods, timing, and gestational age of the infant. Collecting this type of data may include the use of complex, multistage, probability sampling or collecting milk bank samples around the country using a regional food sampling strategy [96] like what is used in the FDA’s Total Diet Study (TDS) [97].

It is unclear whether the amount of contaminants detected in HM in the included studies poses a risk because there are few reference values for comparison. For arsenic, there are no established tolerable intake levels for infants by the WHO [98]. The established values for infant rice cereal (100 ng/g [99]) are not applicable because these are consumed episodically compared with HM. For cadmium, the WHO indicated that exposure ≤20.6 μg/kg body weight per month for children 6 mo to 12 y may be safe [100]. The potential for cadmium exposure to reach those thresholds in infants from HM in the United States is unclear based on the evidence identified in our systematic review. For lead, the United States Centers for Disease Control and Prevention recommends discarding milk that contains ≥40 μg/dL (400 μg/L) of lead [101]. This is to avoid toxicity or poisoning and is orders of magnitude higher than the highest reported value in our results (26 ± 11 μg/L) (46). For mercury, the WHO set 1.4–1.7 μg/L as “normal” in HM [102]. In our results, concentrations in HM collected in Iowa were below this limit (0.93 ± 0.23 μg/L) [48], but were 2–5× higher than “normal” in Alaska where seafood intake is high [49]. For all these contaminants, additional research would be needed to develop reference values for HM, similar to those for drinking water and some infant foods [103,104]. This is particularly important for contaminants in which there are associations between higher maternal exposure and higher HM concentrations, such as lead, mercury, PFOS, and PFOA outlined in a recent systematic review [105]. Reference values would facilitate routine screening during pregnancy to identify high-risk individuals and provide strategies to reduce exposure.

A major limitation precluding conclusions for arsenic, cadmium, lead, and mercury in IF was the lack of recent United States evidence. Of 16, 7 studies were published since 2000, and only 2 in the last decade. Formulations and ingredients of IF have changed over time as well as manufacturing improvements to reduce contamination (e.g., in the early 1970s to reduce lead [18]). More generally, heavy metal exposure in the United States, particularly lead, also decreased since the 1970s because of regulatory changes beyond IF manufacturing (e.g., unleaded gasoline and paint products) [83,106,107]. These systemic changes contributed to an overall reduction of lead in foods. FDA regulations for food contact substances have evolved also, including packaging materials, processing equipment, preparation surfaces, and cookware [108]. Lastly, imported IF have increased, most notably during the shortage in 2022 when Operation Fly Formula used government partnerships to rapidly increase imports [109]. Outdated evidence would not capture these changes over time highlighting the need for routine and transparent data on contaminant assessment in IF, which is recommended as part of Operation Stork that was announced in 2025 [23].

Inadequate methodological reporting further hindered interpretation of the IF evidence for arsenic, cadmium, lead, and mercury. First, most studies used poorly described convenience samples of IF purchased locally. Only the 2 studies about PFAS selected products based on post-2000 market data [65,66]. Second, authors generally did not report brand, product name, formulation (e.g., ingredients, protein-base, nutrients added), processing method, and manufacturing facility or location; only 4 of the post-2000 studies (2 assessed PFAS only) reported the brand and product names [56,58,65,66]. These factors could represent sources of contamination depending on the manufacturing equipment used, as well as variation in water and ingredient sources. Third, there was an inconsistency in the reporting of dilution methods used to prepare the IF samples for analysis, particularly for studies that assessed lead, cadmium, and mercury before 2000. Analyzing undiluted IF will result in higher detected concentrations than diluted IF and could lead to miscalculations of contaminant exposure without proper adjustment. Overall, lack of detail reported for the sampling schemes, product details, and dilution methods in studies that assessed arsenic, cadmium, lead, and mercury precluded our ability to assess whether the analyzed IF were representative of the current United States market and product preparation instructions.

One strategy to improve data availability about contaminant assessment in IF could be to expand on the FDA’s TDS. The TDS is a program that monitors nutrients and contaminants in foods in the United States, including IF. In the TDS, a representative value for a food is modeled and presented as a hybrid mean based on multistage and multi-location sampling across years [110]. The TDS offers a representative value for contaminants which does not currently allow for comparisons across IF types or individual products (e.g., protein-bases, with or without rice starch, or specialty products [17]). Increasing the quantity and types of IF assessed in TDS, as well as reporting product-specific data, could expand the diversity and utility of the TDS program. This strategy could obtain more representative and contemporaneous data about contaminants in IF. It could also facilitate future research because TDS could be linked to other United States dietary databases and national surveillance datasets with health outcomes [111].

FDA regulations require that manufacturers test IF to meet nutritional and safety standards and to keep these records for IF lots. The FDA additionally conducts annual inspections of IF manufacturing facilities and collects and analyzes samples internally [112] for various compounds, including some of those highlighted in this review. Thus, the contaminants of interest may be regularly tested in IF across the country, but these data are not publicly reported or published in peer-reviewed journals. Publicly publishing these data would align with an increase in transparent data reporting practices for infant cereals [113] and other infant foods [[114], [115], [116]] around the world. Although there are challenges and barriers for the formula industry sharing this type of data, this would align with the current consumer demand for increased transparency and traceability of the food supply more generally [117,118].

Our systematic review has several strengths. We followed rigorous methodological standards of PRISMA [27] and AMSTAR 2 [28] to ensure that the a priori registered protocol and methods were of high quality. We also had 2 different TEPs (1 for protocol development and 1 for critically appraising the methods of included articles). We had experts in library sciences and systematic review methodology develop and peer review the database search. The FDA commissioned this systematic review, which was conducted independently by our research team to minimize bias. The FDA set out to summarize only published, peer-reviewed literature to complement internally assessed data so we did not include gray literature or clinical trial registries and did not contact authors for unpublished data. Therefore, our systematic review does not represent the full scope of information available, particularly for IF. This could be considered a limitation, which is why the certainty of evidence for PFAS in IF was downgraded for publication bias risk. Another limitation for IF was that we created a ROB assessment to address bias particular to the research question, but it was not validated or tested for inter-rater reliability.

Lastly, given that the FDA’s mission pertains to the safety of the United States food supply, we only synthesized data from the United States and presented data from other countries in a heat map. Our heat maps can help identify priorities for future systematic reviews on the evidence available in other relevant countries to help supplement these knowledge gaps for the United States. Future systematic reviews on these data could also facilitate comparisons across countries. However, the evidence from other countries may have similar limitations as those described in detail for the United States.

In conclusion, we found that PFOA and PFOS were detected in HM samples in the United States, indicating the importance of minimizing exposure to the large class of PFAS chemicals during pregnancy and lactation. PFAS were not detected in IF assessed in studies from the United States but at-home preparation, such as adding water to powdered formula, may introduce contaminants depending on the water source. There was a notable lack of peer-reviewed evidence assessing arsenic, cadmium, lead, and mercury in HM or IF in the United States. Evidence that we identified from other countries should be further examined in systematic reviews to help address these research gaps. National biomonitoring of HM and transparency of routine data collected by IF manufacturers are also needed.

## Author contributions

The authors’ responsibilities were as follows – MKS, AJM: designed the research; KGD, MMF: served on the Technical Expert Panel to inform the protocol; MJF, KMH: designed and conducted the literature search; RCT, LEO, AAB, RT, SMA, TB, MKS, AJM: conducted the research; RCT, LEO, AAB, SMA, CNU: prepared the data; ALG, CAH: served on the Technical Expert Panel to critically appraise sample collection procedures and contaminant analytic methods; LEO: synthesized the data; RCT, LEO: wrote the paper; CNU, MKS, AJM: edited the paper; and all authors: read and approved the final manuscript.

## Data availability

All data are described in detail in the tables and supplementary materials.

## Funding

Funding was provided by the US Department of Health and Human Services—Food and Drug Administration contract #75F40123P00255.

## Conflict of interest

AJM serves as an Associate Editor for *The American Journal of Clinical Nutrition*. All other authors report no conflicts of interest.
