# Supplementary material for: Assessment of arsenic, cadmium, lead, mercury, and per- and polyfluoroalkyl substances concentrations in human milk and infant formula in the United States: a systematic review
Source: Am J Clin Nutr. 2025 Sep 23;122(4):1006–26. doi: 10.1016/j.ajcnut.2025.07.039 (PMC12674019; doi:10.1016/j.ajcnut.2025.07.039)
Supplement: Supplementary file 1 [file mmc1.docx]

**SUPPLEMENTAL MATERIAL**

Contents

[**Supplemental Table 1.** Series of systematic review questions related to potential contaminants in human milk and infant formula commissioned by the US Food and Drug Administration’s Human Foods Program within its mission area of food chemical safety.	2](#_Toc204586448)

[**Supplemental Table 2.** Electronic database search strategy for questions 2-6 listed in Supplemental Table 1.	3](#_Toc204586449)

[**Supplemental Table 3.** Inclusion/exclusion criteria for a systematic review about the composition/variability of contaminants in human milk and infant formula (PROSPERO: CRD42024528756).	15](#_Toc204586450)

[**Supplemental Table 4.** Operationalization of ROBINS-E^1^ for studies assessing contaminants in human milk.	17](#_Toc204586451)

[**Supplemental Table 5.** Risk of bias domains used for studies assessing contaminants in infant formula.	19](#_Toc204586452)

[**Supplemental Table 6.** Full text articles excluded at confirmatory review for human milk with reason.^1^	20](#_Toc204586453)

[**Supplemental Table 7.** Reference list of human milk articles included in heat map.	22](#_Toc204586454)

[**Supplemental Table 8.** Full text articles excluded at confirmatory review for infant formula with reason.^1^	54](#_Toc204586455)

[**Supplemental Table 9.** Study characteristics of infant formula studies published before the year 2000.	56](#_Toc204586456)

[**Supplemental Table 10.** Results of infant formula studies published before the year 2000.^1,2^	58](#_Toc204586457)

[**Supplemental Table 11.** Reference list of infant formula articles included in heat map.	60](#_Toc204586458)

[**Supplemental Table 12.** Details of infant formula products as described by authors of the United States studies.	70](#_Toc204586459)

[**Supplemental Figure 1.** Analytic framework.	84](#_Toc204586460)

[**Supplemental Figure 2.** Heat map of published literature^1^ on assessed contaminants in human milk from countries that are ‘high’ or ‘very high’ on the Human Development Index.^2^	85](#_Toc204586461)

[**Supplemental Figure 3.** Heat map of published literature^1^ on assessed contaminants in infant formula from countries that were ‘high’ or ‘very high’ on the Human Development Index^2^.	87](#_Toc204586462)

[**Appendix A.** PRISMA 2020 checklist.	89](#_Toc204586463)

[**Appendix B.** AMSTAR 2 checklist.	94](#_Toc204586464)

[**References**	99](#_Toc204586465)

## **Supplemental Table 1.** Series of systematic review questions related to potential contaminants in human milk and infant formula commissioned by the US Food and Drug Administration’s Human Foods Program within its mission area of food chemical safety.

1. What is the relationship between exposure to contaminants during pregnancy and lactation and contaminant concentrations in human milk? (International Prospective Register of Systematic Reviews [PROSPERO]: CRD42024530326)

2. What is the composition/variability of contaminants in human milk and infant formula? (PROSPERO: CRD42024528756)

3. What is the bioavailability of contaminants from human milk and infant formula when consumed by the infant? (PROSPERO: CRD42024530332)

4. What factors impact the bioavailability of contaminants from human milk and infant formula when consumed by the infant? (PROSPERO: CRD42024530336)

5. What is the relationship between contaminants and the bioavailability of other components in human milk and infant formula? (PROSPERO: CRD42024530339)

6. What are the observed contaminant concentrations in infants in the United States consuming human milk and/or infant formula? (PROSPERO: CRD42024530344)

PROSPERO, International Prospective Register of Systematic Reviews.

## **Supplemental Table 2.** Electronic database search strategy for questions 2-6 listed in Supplemental Table 1.

Librarian(s): Margaret Foster, Kyle Holland

Peer-reviewer name and/or organization: Sheila Green, Texas A&M University Medical Sciences Library

Peer-review date: April 8, 2024

Total number of records identified from each database:

**MEDLINE (Ovid):** 2418

**Embase (Ovid):** 3232

**CAB Abstracts (Ovid):** 3451^1^

**CINAHL (EBSCO):** 343

**CENTRAL (Cochrane Library):** 439

Number of duplicates removed outside of Distiller: 3321

Number of duplicates removed in Distiller: 307

**Hand search of Journal of Environmental Exposure Assessment**: 26

Number of duplicates removed from journal hand search: 13

^1^This database was added after PROSPERO registration.

Search strategy for each database:

Resources that contributed important and/or substantial elements to the below search strings.

| Resource | Contribution |
| --- | --- |
| Frigerio, G., Ferrari, C.M. & Fustinoni, S. Prenatal and childhood exposure to per-/polyfluoroalkyl substances (PFASs) and its associations with childhood overweight and/or obesity: a systematic review with meta-analyses. *Environ Health* 22, 56 (2023). https://doi.org/10.1186/s12940-023-01006-6 | Many PFAS terms sourced. |

Database: MEDLINE

Platform: Ovid

Date of search: April 2, 2025

Limits: Language: English; Not animal studies

| Concept | Line | Search Strategy |
| --- | --- | --- |
| human milk | 1 | exp Milk, Human/ |
|  | 2 | (((human? or breast* or maternal or mother*) adj milk*) or breastmilk? or colostrum).ti,ab,kf. |
|  | 3 | or/1-2 |
| infant formula | 4 | exp Infant Formula/ or exp Bottle Feeding/ |
|  | 5 | (((baby or babies or newborn* or "new born*" or infant* or neonat* or "neo nat*") adj formula*) or bottlefe* or (bottle adj (feed* or fed))).ti,ab,kf. |
|  | 6 | or/4-5 |
| pregnancy | 7 | exp Pregnancy/ or exp Pregnancy Complications/ or exp Pregnancy Outcome/ |
|  | 8 | (pregnan* or prenatal* or "pre natal*" or postnatal* or "post natal*" or antenatal* or "ante natal*" or perinatal* or "peri natal*" or postpartum or "post partum" or f?etus* or f?etal or maternal or gravid* or birth*).ti,ab,kf. |
|  | 9 | or/7-8 |
| lactation | 10 | exp Lactation/ or exp Breast Feeding/ |
|  | 11 | (lactat* or breastfe* or (breast adj (fed or feed*))).ti,ab,kf. |
|  | 12 | or/10-11 |
| neonates | 13 | exp Infant/ |
|  | 14 | ((age? adj2 ("0" or "1" or "2" or zero or one or two)) or (("0" or "1" or "2" or zero or one or two) adj2 ("year? of age" or "yr or age" or "ys or age" or "yrs or age" or "y or age" or "year? old" or "yr old" or "ys old" or "yrs old" or "y old")) or ((month? or mth? or ms or m or week? or wks or wk or "w" or day? or ds or d) adj2 (old or "of age")) or (age? adj3 (month? or mth? or week? or wks or wk or day?)) or infan* or baby or babies or newborn* or "new born*" or neonat* or "neo nat*" or preemie?).ti,ab,kf. |
|  | 15 | or/13-14 |
| contaminants | 16 | exp Mercury/ or exp Mercury Compounds/ or exp Organomercury Compounds/ or exp Mercury Poisoning/ |
|  | 17 | (mercur* or methylmercur* or organomercur* or MeHg or CH3Hg or (Hg and (metal* or organometal* or cation* or ion* or isotop* or radioisotop* or element* or microelement* or organic or inorganic))).ti,ab,kf. |
|  | 18 | exp Lead Poisoning/ or exp Lead/ or exp Lead Radioisotopes/ or exp Tetraethyl Lead/ |
|  | 19 | lead.ti,kf,nm. or (pb or 208Pb or organolead* or plumbum or plumbic* or plumbate* or plumbous or tetraethyllead or (lead adj8 ("208" or metal* or organometal* or cation* or ion* or isotop* or radioisotop* or element* or microelement* or organic or inorganic or ore or blood* or chemical? or poison* or contam*))).ti,ab,kf. |
|  | 20 | exp Arsenic Poisoning/ or exp Arsenic/ or exp Arsenicals/ |
|  | 21 | ((arsen$2 not arsenal) or arsen##$3 or arsine$1 or organoars*).ti,ab,kf. |
|  | 22 | exp Cadmium/ or exp Cadmium Poisoning/ or exp Cadmium Radioisotopes/ or exp Cadmium Compounds/ |
|  | 23 | (cadmi* or organocadmi* or (Cd and (metal* or organometal* or cation* or ion* or isotop* or radioisotop* or element* or microelement* or organic or inorganic))).ti,ab,kf. |
|  | 24 | exp Fluorocarbons/ |
|  | 25 | (perfluor* or polyfluor* or methylperfluor* or ethylperfluor* or fluorocarb* or fluorochemical* or fluorosurfactant* or fluorotelomer* or fluorinate* or PFAS$1 or PFBA$1 or PFBS$1 or PFC$1 or PFCA$1 or PFDA$1 or PFDoDA$1 or PFDS$1 or PFECHS$1 or PFESA$1 or PFEtCHxS$1 or PFHp$1 or PFHpA$1 or PFHpS$1 or PFHxA$1 or PFHxS$1 or PFNA$1 or PFNS$1 or PFOA$1 or PFOS$1 or PFOSA$1 or PFPA$1 or PFPeA$1 or PFPeS$1 or PFPiA$1 or PFSA$1 or PFTE$1 or PFTeDA$1 or PFTrDA$1 or PFUnDA$1 or FOSA$1 or EtFOSA$1 or MeFOSA$1 or DONA$1 or FTOH$1 or FTSA$1 or HFPO$1 or LCPFA$1 or SCPFA$1 or Teflon or freon or GenX).ti,ab,kf. |
|  | 26 | Metals, Heavy/ or Heavy Metal Poisoning/ or (heavy adj metal*).ti,ab,kf. |
|  | 27 | or/16-26 |
| no animal studies | 28 | (exp Animals/ not (exp Animals/ and Humans/)) or exp Animal Experimentation/ or exp Models, Animal/ |
|  | 29 | ((animal? adj2 (study or model* or experiment*)) or mouse or mice or rat or rats or monkey or monkeys or "preclinical study").ti. |
|  | 30 | or/28-29 |
| Protocol Question 2-6 | 31 | ((3 or 6 or 12) and 27) not 30 |
|  | 32 | .l/ 31 en=y |

Database: Embase

Platform: Ovid

Date of search: April 2, 2025

Limits: English Language; Not animal studies

| Concept | Line | | | Search Strategy |
| --- | --- | --- | --- | --- |
| human milk & infant | | 1 | exp infant feeding/ or exp baby food/ | |
| formula | | 2 | (((human? or breast* or maternal or mother*) adj milk*) or breastmilk? or colostrum or (((baby or babies or newborn* or "new born*" or infant* or neonat* or "neo nat*") adj formula*) or bottlefe* or (bottle adj (feed* or fed)))).ti,ab,kf. | |
|  | | 3 | or/1-2 | |
| pregnancy | | 4 | exp pregnancy/ or exp pregnancy disorder/ or exp "parameters concerning the fetus, newborn and pregnancy"/ | |
|  | | 5 | (pregnan* or prenatal* or "pre natal*" or postnatal* or "post natal*" or antenatal* or "ante natal*" or perinatal* or "peri natal*" or postpartum or "post partum" or f?etus* or f?etal or maternal or gravid* or birth*).ti,ab,kf. | |
|  | | 6 | or/4-5 | |
| lactation | | 7 | lactation/ or exp breast feeding/ | |
|  | | 8 | (lactat* or breastfe* or (breast adj (fed or feed*))).ti,ab,kf. | |
|  | | 9 | or/7-8 | |
| neonates | | 10 | exp infant/ | |
|  | | 11 | ((age? adj2 ("0" or "1" or "2" or zero or one or two)) or (("0" or "1" or "2" or zero or one or two) adj2 ("year? of age" or "yr or age" or "ys or age" or "yrs or age" or "y or age" or "year? old" or "yr old" or "ys old" or "yrs old" or "y old")) or ((month? or mth? or ms or m or week? or wks or wk or "w" or day? or ds or d) adj2 (old or "of age")) or (age? adj3 (month? or mth? or week? or wks or wk or day?)) or infan* or baby or babies or newborn* or "new born*" or neonat* or "neo nat*" or preemie?).ti,ab,kf. | |
|  | | 12 | or/10-11 | |
| contaminants | | 13 | mercury/ or mercury derivative/ or exp mercurialism/ or exp organomercury compound/ | |
|  | | 14 | (mercur* or methylmercur* or organomercur* or MeHg or CH3Hg or (Hg and (metal* or organometal* or cation* or ion* or isotop* or radioisotop* or element* or microelement* or organic or inorganic))).ti,ab,kf. | |
|  | | 15 | lead/ or lead poisoning/ or exp organolead compound/ | |
|  | | 16 | lead.ti,kf,hw. or (pb or 208Pb or organolead* or plumbum or plumbic* or plumbate* or plumbous or tetraethyllead or (lead adj8 ("208" or metal* or organometal* or cation* or ion* or isotop* or radioisotop* or element* or microelement* or organic or inorganic or ore or blood* or chemical? or poison* or contam*))).ti,ab,kf. | |
|  | | 17 | arsenic/ or arsenic derivative/ or arsenic poisoning/ or exp arsine derivative/ or exp organoarsenic derivative/ | |
|  | | 18 | ((arsen$2 not arsenal) or arsen##$3 or arsine$1 or organoars*).ti,ab,kf. | |
|  | | 19 | cadmium/ or cadmium derivative/ or cadmium poisoning/ | |
|  | | 20 | (cadmi* or organocadmi* or (Cd and (metal* or organometal* or cation* or ion* or isotop* or radioisotop* or element* or microelement* or organic or inorganic))).ti,ab,kf. | |
|  | | 21 | exp perfluoroalkanoic acid/ or exp organofluorine derivative/ or polytetrafluoroethylene/ or freon/ | |
|  | | 22 | (perfluor* or polyfluor* or methylperfluor* or ethylperfluor* or fluorocarb* or fluorochemical* or fluorosurfactant* or fluorotelomer* or fluorinate* or PFAS$1 or PFBA$1 or PFBS$1 or PFC$1 or PFCA$1 or PFDA$1 or PFDoDA$1 or PFDS$1 or PFECHS$1 or PFESA$1 or PFEtCHxS$1 or PFHp$1 or PFHpA$1 or PFHpS$1 or PFHxA$1 or PFHxS$1 or PFNA$1 or PFNS$1 or PFOA$1 or PFOS$1 or PFOSA$1 or PFPA$1 or PFPeA$1 or PFPeS$1 or PFPiA$1 or PFSA$1 or PFTE$1 or PFTeDA$1 or PFTrDA$1 or PFUnDA$1 or FOSA$1 or EtFOSA$1 or MeFOSA$1 or DONA$1 or FTOH$1 or FTSA$1 or HFPO$1 or LCPFA$1 or SCPFA$1 or Teflon or freon or GenX).ti,ab,kf. | |
|  | | 23 | heavy metal/ or heavy metal blood level/ or heavy metal poisoning/ or (heavy adj metal*).ti,ab,kf. | |
|  | | 24 | or/13-23 | |
| no animal studies | | 25 | (exp animal/ not (exp animal/ and exp human/)) or nonhuman/ or exp animal experiment/ or exp experimental organism/ | |
|  | | 26 | ((animal? adj2 (study or model* or experiment*)) or mouse or mice or rat or rats or monkey or monkeys or "preclinical study").ti. | |
|  | | 27 | or/25-26 | |
| Protocol Question 2-6 | | 28 | ((3 or 9) and 24) not 27 | |
|  | | 29 | .l/ 28 en=y | |

Database: CAB Abstracts

This database was searched but not mentioned in PROSPERO.

Platform: Ovid

Date of search: April 2, 2025 Limits: English Language; Not animal studies

| Concept | | Line | Search Strategy |
| --- | --- | --- | --- |
| human milk | 1 | | human milk/ |
|  | 2 | | (((human? or breast* or maternal or mother*) adj milk*) or breastmilk? or colostrum).ti,ot,ab,id,hw. |
|  | 3 | | or/1-2 |
| infant formula | 4 | | infant formulae/ or bottle feeding/ or humanized milk/ |
|  | 5 | | (((baby or babies or newborn* or "new born*" or infant* or neonat* or "neo nat*") adj formula*) or bottlefe* or (bottle adj (feed* or fed))).ti,ot,ab,id,hw. |
|  | 6 | | or/4-5 |
| pregnancy | 7 | | exp pregnancy/ or exp pregnancy complications/ or pregnancy outcome/ or pregnant women/ or exp birth/ |
|  | 8 | | (pregnan* or prenatal* or "pre natal*" or postnatal* or "post natal*" or antenatal* or "ante natal*" or perinatal* or "peri natal*" or postpartum or "post partum" or f?etus* or f?etal or maternal or gravid* or birth*).ti,ot,ab,id,hw. |
|  | 9 | | or/7-8 |
| lactation | 10 | | lactation/ or human lactation/ or exp lactating women/ or exp breast feeding/ |
|  | 11 | | (lactat* or breastfe* or (breast adj (fed or feed*))).ti,ot,ab,id,hw. |
|  | 12 | | or/10-11 |
| neonates | 13 | | exp infants/ |
|  | 14 | | ((age? adj2 ("0" or "1" or "2" or zero or one or two)) or (("0" or "1" or "2" or zero or one or two) adj2 ("year? of age" or "yr or age" or "ys or age" or "yrs or age" or "y or age" or "year? old" or "yr old" or "ys old" or "yrs old" or "y old")) or ((month? or mth? or ms or m or week? or wks or wk or "w" or day? or ds or d) adj2 (old or "of age")) or (age? adj3 (month? or mth? or week? or wks or wk or day?)) or infan* or baby or babies or newborn* or "new born*" or neonat* or "neo nat*" or preemie?).ti,ot,ab,id,hw. |
|  | 15 | | or/13-14 |
| contaminants | 16 | | mercury/ or exp mercury compounds/ or exp mercury fungicides/ |
|  | 17 | | (mercur* or methylmercur* or organomercur* or MeHg or CH3Hg or (Hg and (metal* or organometal* or cation* or ion* or isotop* or radioisotop* or element* or microelement* or organic or inorganic))).ti,ot,ab,id,hw. |
|  | 18 | | lead/ or lead poisoning/ |
|  | 19 | | (lead or pb or 208Pb or organolead* or plumbum or plumbic* or plumbate* or plumbous or tetraethyllead or (lead adj8 ("208" or metal* or organometal* or cation* or ion* or isotop* or radioisotop* or element* or microelement* or organic or inorganic or ore or blood* or chemical? or poison* or contam*))).ti,ot,ab,id,hw. |
|  | 20 | | arsenic/ or exp arsenicals/ |
|  | 21 | | ((arsen$2 not arsenal) or arsen##$3 or arsine$1 or organoars*).ti,ot,ab,id,hw. |
|  | 22 | | cadmium/ |
|  | 23 | | (cadmi* or organocadmi* or (Cd and (metal* or organometal* or cation* or ion* or isotop* or radioisotop* or element* or microelement* or organic or inorganic))).ti,ot,ab,id,hw. |
|  | 24 | | exp organofluorine compounds/ or exp organofluorine rodenticides/ or freons/ |
|  | 25 | | (perfluor* or polyfluor* or methylperfluor* or ethylperfluor* or fluorocarb* or fluorochemical* or fluorosurfactant* or fluorotelomer* or fluorinate* or PFAS$1 or PFBA$1 or PFBS$1 or PFC$1 or PFCA$1 or PFDA$1 or PFDoDA$1 or PFDS$1 or PFECHS$1 or PFESA$1 or PFEtCHxS$1 or PFHp$1 or PFHpA$1 or PFHpS$1 or PFHxA$1 or PFHxS$1 or PFNA$1 or PFNS$1 or PFOA$1 or PFOS$1 or PFOSA$1 or PFPA$1 or PFPeA$1 or PFPeS$1 or PFPiA$1 or PFSA$1 or PFTE$1 or PFTeDA$1 or PFTrDA$1 or PFUnDA$1 or FOSA$1 or EtFOSA$1 or MeFOSA$1 or DONA$1 or FTOH$1 or FTSA$1 or HFPO$1 or LCPFA$1 or SCPFA$1 or Teflon or freon or GenX).ti,ot,ab,id,hw. |
|  | 26 | | heavy metals/ or (heavy adj metal*).ti,ot,ab,id,hw. |
|  | 27 | | or/16-26 |
| no animal studies | 28 | | ((animals or plants or fungi or prokaryotes or viruses).sh,bt. not man/) or animal experiments/ or exp laboratory animals/ or animal models/ |
|  | 29 | | ((animal? adj2 (study or model* or experiment*)) or mouse or mice or rat or rats or monkey or monkeys or "preclinical study").ti. |
|  | 30 | | or/28-29 |
| Protocol Question 2-6 | 31 | | ((3 or 6 or 12) and 27) not 30 |
|  | 32 | | limit 31 to english |

Database: CINAHL

Platform: EBSCO

Date of search: April 2, 2025

Limits: English Language; Not animal studies

Expanders: Apply equivalent subjects

| Concept | | Line | Search Strategy |
| --- | --- | --- | --- |
| human milk | S1 | | (MH "Milk, Human+") or (MH "Milk Banks") |
|  | S2 | | ((((TI human# OR AB human# OR SU human#) OR (TI breast* OR AB breast* OR SU breast*) OR (TI maternal OR AB maternal OR SU maternal) OR (TI mother* OR AB mother* OR SU mother*)) W1 (TI milk* OR AB milk* OR SU milk*)) OR (TI breastmilk# OR AB breastmilk# OR SU breastmilk#) OR (TI colostrum OR AB colostrum OR SU colostrum)) |
|  | S3 | | S1 OR S2 |
| infant formula | S4 | | (MH "Infant Formula+") |
|  | S5 | | ((((TI baby OR AB baby OR SU baby) OR (TI babies OR AB babies OR SU babies) OR (TI newborn* OR AB newborn* OR SU newborn*) OR (TI "new born*" OR AB "new born*" OR SU "new born*") OR (TI infant* OR AB infant* OR SU infant*) OR (TI neonat* OR AB neonat* OR SU neonat*) OR (TI "neo nat*" OR AB "neo nat*" OR SU "neo nat*")) W1 (TI formula* OR AB formula* OR SU formula*)) OR (TI bottlefe* OR AB bottlefe* OR SU bottlefe*) OR ((TI bottle OR AB bottle OR SU bottle) W1 ((TI feed* OR AB feed* OR SU feed*) OR (TI fed OR AB fed OR SU fed)))) |
|  | S6 | | S4 OR S5 |
| pregnancy | S7 | | (MH "Pregnancy") |
|  | S8 | | ((TI pregnan* OR AB pregnan* OR SU pregnan*) OR (TI prenatal* OR AB prenatal* OR SU prenatal*) OR (TI "pre natal*" OR AB "pre natal*" OR SU "pre natal*") OR (TI postnatal* OR AB postnatal* OR SU postnatal*) OR (TI "post natal*" OR AB "post natal*" OR SU "post natal*") OR (TI antenatal* OR AB antenatal* OR SU antenatal*) OR (TI "ante natal*" OR AB "ante natal*" OR SU "ante natal*") OR (TI perinatal* OR AB perinatal* OR SU perinatal*) OR (TI "peri natal*" OR AB "peri natal*" OR SU "peri natal*") OR (TI postpartum OR AB postpartum OR SU postpartum) OR (TI "post partum" OR AB "post partum" OR SU "post partum") OR (TI f#etus* OR AB f#etus* OR SU f#etus*) OR (TI f#etal OR AB f#etal OR SU f#etal) OR (TI maternal OR AB maternal OR SU maternal) OR (TI gravid* OR AB gravid* OR SU gravid*) OR (TI birth* OR AB birth* OR SU birth*)) |
|  | S9 | | S7 OR S8 |
| lactation | S10 | | (MH "Lactation") or (MH "Infant Feeding+") or (MH "Infant Nutrition+") |
|  | S11 | | ((TI lactat* OR AB lactat* OR SU lactat*) OR (TI breastfe* OR AB breastfe* OR SU breastfe*) OR ((TI breast OR AB breast OR SU breast) W1 ((TI fed OR AB fed OR SU fed) OR (TI feed* OR AB feed* OR SU feed*)))) |
|  | S12 | | S10 OR S11 |
| neonates | S13 | | (MH "Infant+") |
|  | S14 | | (((TI age# OR AB age# OR SU age#) N2 ((TI 0 OR AB 0 OR SU 0) OR (TI 1 OR AB 1 OR SU 1) OR (TI 2 OR AB 2 OR SU 2) OR (TI zero OR AB zero OR SU zero) OR (TI one OR AB one OR SU one) OR (TI two OR AB two OR SU two))) OR (((TI 0 OR AB 0 OR SU 0) OR (TI 1 OR AB 1 OR SU 1) OR (TI 2 OR AB 2 OR SU 2) OR (TI zero OR AB zero OR SU zero) OR (TI one OR AB one OR SU one) OR (TI two OR AB two OR SU two)) N2 ((TI "year# of age" OR AB "year# of age" OR SU "year# of age") OR (TI "yr or age" OR AB "yr or age" OR SU "yr or age") OR (TI "ys or age" OR AB "ys or age" OR SU "ys or age") OR (TI "yrs or age" OR AB "yrs or age" OR SU "yrs or age") OR (TI "y or age" OR AB "y or age" OR SU "y or age") OR (TI "year# old" OR AB "year# old" OR SU "year# old") OR (TI "yr old" OR AB "yr old" OR SU "yr old") OR (TI "ys old" OR AB "ys old" OR SU "ys old") OR (TI "yrs old" OR AB "yrs old" OR SU "yrs old") OR (TI "y old" OR AB "y old" OR SU "y old"))) OR (((TI month# OR AB month# OR SU month#) OR (TI mth# OR AB mth# OR SU mth#) OR (TI ms OR AB ms OR SU ms) OR (TI m OR AB m OR SU m) OR (TI week# OR AB week# OR SU week#) OR (TI wks OR AB wks OR SU wks) OR (TI wk OR AB wk OR SU wk) OR (TI w OR AB w OR SU w) OR (TI day# OR AB day# OR SU day#) OR (TI ds OR AB ds OR SU ds) OR (TI d OR AB d OR SU d)) N2 ((TI old OR AB old OR SU old) OR (TI "of age" OR AB "of age" OR SU "of age"))) OR ((TI age# OR AB age# OR SU age#) N3 ((TI month# OR AB month# OR SU month#) OR (TI mth# OR AB mth# OR SU mth#) OR (TI week# OR AB week# OR SU week#) OR (TI wks OR AB wks OR SU wks) OR (TI wk OR AB wk OR SU wk) OR (TI day# OR AB day# OR SU day#))) OR (TI infan* OR AB infan* OR SU infan*) OR (TI baby OR AB baby OR SU baby) OR (TI babies OR AB babies OR SU babies) OR (TI newborn* OR AB newborn* OR SU newborn*) OR (TI "new born*" OR AB "new born*" OR SU "new born*") OR (TI neonat* OR AB neonat* OR SU neonat*) OR (TI "neo nat*" OR AB "neo nat*" OR SU "neo nat*") OR (TI preemie# OR AB preemie# OR SU preemie#)) |
|  | S15 | | S13 OR S14 |
| contaminants | S16 | | (MH "Mercury") or (MH "Mercury Poisoning") |
|  | S17 | | ((TI mercur* OR AB mercur* OR SU mercur*) OR (TI methylmercur* OR AB methylmercur* OR SU methylmercur*) OR (TI organomercur* OR AB organomercur* OR SU organomercur*) OR (TI MeHg OR AB MeHg OR SU MeHg) OR (TI CH3Hg OR AB CH3Hg OR SU CH3Hg) OR ((TI Hg OR AB Hg OR SU Hg) AND ((TI metal* OR AB metal* OR SU metal*) OR (TI organometal* OR AB organometal* OR SU organometal*) OR (TI cation* OR AB cation* OR SU cation*) OR (TI ion* OR AB ion* OR SU ion*) OR (TI isotop* OR AB isotop* OR SU isotop*) OR (TI radioisotop* OR AB radioisotop* OR SU radioisotop*) OR (TI element* OR AB element* OR SU element*) OR (TI microelement* OR AB microelement* OR SU microelement*) OR (TI organic OR AB organic OR SU organic) OR (TI inorganic OR AB inorganic OR SU inorganic)))) |
|  | S18 | | (MH "Lead") or (MH "Lead Exposure") or (MH "Lead Poisoning") |
|  | S19 | | (TI lead,kf.) OR ((TI pb OR AB pb OR SU pb) OR (TI 208Pb OR AB 208Pb OR SU 208Pb) OR (TI organolead* OR AB organolead* OR SU organolead*) OR (TI plumbum OR AB plumbum OR SU plumbum) OR (TI plumbic* OR AB plumbic* OR SU plumbic*) OR (TI plumbate* OR AB plumbate* OR SU plumbate*) OR (TI plumbous OR AB plumbous OR SU plumbous) OR (TI tetraethyllead OR AB tetraethyllead OR SU tetraethyllead) OR ((TI lead OR AB lead OR SU lead) N8 ((TI 208 OR AB 208 OR SU 208) OR (TI metal* OR AB metal* OR SU metal*) OR (TI organometal* OR AB organometal* OR SU organometal*) OR (TI cation* OR AB cation* OR SU cation*) OR (TI ion* OR AB ion* OR SU ion*) OR (TI isotop* OR AB isotop* OR SU isotop*) OR (TI radioisotop* OR AB radioisotop* OR SU radioisotop*) OR (TI element* OR AB element* OR SU element*) OR (TI microelement* OR AB microelement* OR SU microelement*) OR (TI organic OR AB organic OR SU organic) OR (TI inorganic OR AB inorganic OR SU inorganic) OR (TI ore OR AB ore OR SU ore) OR (TI blood* OR AB blood* OR SU blood*) OR (TI chemical# OR AB chemical# OR SU chemical#) OR (TI poison* OR AB poison* OR SU poison*) OR (TI contam* OR AB contam* OR SU contam*)))) |
|  | S20 | | (MH "Arsenic") or (MH "Arsenicals+") or (MH "Arsenic Poisoning") |
|  | S21 | | (((TI arsen* OR AB arsen* OR SU arsen*) NOT (TI arsenal OR AB arsenal OR SU arsenal)) OR (TI arsine# OR AB arsine# OR SU arsine#) OR (TI organoars* OR AB organoars* OR SU organoars*)) |
|  | S22 | | (MH "Cadmium") or (MH "Cadmium Compounds+") |
|  | S23 | | ((TI cadmi* OR AB cadmi* OR SU cadmi*) OR (TI organocadmi* OR AB organocadmi* OR SU organocadmi*) OR ((TI Cd OR AB Cd OR SU Cd) AND ((TI metal* OR AB metal* OR SU metal*) OR (TI organometal* OR AB organometal* OR SU organometal*) OR (TI cation* OR AB cation* OR SU cation*) OR (TI ion* OR AB ion* OR SU ion*) OR (TI isotop* OR AB isotop* OR SU isotop*) OR (TI radioisotop* OR AB radioisotop* OR SU radioisotop*) OR (TI element* OR AB element* OR SU element*) OR (TI microelement* OR AB microelement* OR SU microelement*) OR (TI organic OR AB organic OR SU organic) OR (TI inorganic OR AB inorganic OR SU inorganic)))) |
|  | S24 | | (MH “Hydrocarbons, Fluorinated+”) |
|  | S25 | | ((TI perfluor* OR AB perfluor* OR SU perfluor*) OR (TI polyfluor* OR AB polyfluor* OR SU polyfluor*) OR (TI methylperfluor* OR AB methylperfluor* OR SU methylperfluor*) OR (TI ethylperfluor* OR AB ethylperfluor* OR SU ethylperfluor*) OR (TI fluorocarb* OR AB fluorocarb* OR SU fluorocarb*) OR (TI fluorochemical* OR AB fluorochemical* OR SU fluorochemical*) OR (TI fluorosurfactant* OR AB fluorosurfactant* OR SU fluorosurfactant*) OR (TI fluorotelomer* OR AB fluorotelomer* OR SU fluorotelomer*) OR (TI fluorinate* OR AB fluorinate* OR SU fluorinate*) OR (TI PFAS# OR AB PFAS# OR SU PFAS#) OR (TI PFBA# OR AB PFBA# OR SU PFBA#) OR (TI PFBS# OR AB PFBS# OR SU PFBS#) OR (TI PFC# OR AB PFC# OR SU PFC#) OR (TI PFCA# OR AB PFCA# OR SU PFCA#) OR (TI PFDA# OR AB PFDA# OR SU PFDA#) OR (TI PFDoDA# OR AB PFDoDA# OR SU PFDoDA#) OR (TI PFDS# OR AB PFDS# OR SU PFDS#) OR (TI PFECHS# OR AB PFECHS# OR SU PFECHS#) OR (TI PFESA# OR AB PFESA# OR SU PFESA#) OR (TI PFEtCHxS# OR AB PFEtCHxS# OR SU PFEtCHxS#) OR (TI PFHp# OR AB PFHp# OR SU PFHp#) OR (TI PFHpA# OR AB PFHpA# OR SU PFHpA#) OR (TI PFHpS# OR AB PFHpS# OR SU PFHpS#) OR (TI PFHxA# OR AB PFHxA# OR SU PFHxA#) OR (TI PFHxS# OR AB PFHxS# OR SU PFHxS#) OR (TI PFNA# OR AB PFNA# OR SU PFNA#) OR (TI PFNS# OR AB PFNS# OR SU PFNS#) OR (TI PFOA# OR AB PFOA# OR SU PFOA#) OR (TI PFOS# OR AB PFOS# OR SU PFOS#) OR (TI PFOSA# OR AB PFOSA# OR SU PFOSA#) OR (TI PFPA# OR AB PFPA# OR SU PFPA#) OR (TI PFPeA# OR AB PFPeA# OR SU PFPeA#) OR (TI PFPeS# OR AB PFPeS# OR SU PFPeS#) OR (TI PFPiA# OR AB PFPiA# OR SU PFPiA#) OR (TI PFSA# OR AB PFSA# OR SU PFSA#) OR (TI PFTE# OR AB PFTE# OR SU PFTE#) OR (TI PFTeDA# OR AB PFTeDA# OR SU PFTeDA#) OR (TI PFTrDA# OR AB PFTrDA# OR SU PFTrDA#) OR (TI PFUnDA# OR AB PFUnDA# OR SU PFUnDA#) OR (TI FOSA# OR AB FOSA# OR SU FOSA#) OR (TI EtFOSA# OR AB EtFOSA# OR SU EtFOSA#) OR (TI MeFOSA# OR AB MeFOSA# OR SU MeFOSA#) OR (TI DONA# OR AB DONA# OR SU DONA#) OR (TI FTOH# OR AB FTOH# OR SU FTOH#) OR (TI FTSA# OR AB FTSA# OR SU FTSA#) OR (TI HFPO# OR AB HFPO# OR SU HFPO#) OR (TI LCPFA# OR AB LCPFA# OR SU LCPFA#) OR (TI SCPFA# OR AB SCPFA# OR SU SCPFA#) OR (TI Teflon OR AB Teflon OR SU Teflon) OR (TI freon OR AB freon OR SU freon) OR (TI GenX OR AB GenX OR SU GenX)) |
|  | S26 | | (MH "Metals, Heavy") OR (MH "Heavy Metal Poisoning") OR ((TI heavy OR AB heavy OR SU heavy) W1 (TI metal* OR AB metal* OR SU metal*)) |
|  | S27 | | S16 OR S17 OR S18 OR S19 OR S20 OR S21 OR S22 OR S23 OR S24 OR S25 OR S26 |
| no animal studies | S28 | | ((MH Animals+) NOT ((MH Animals+) AND (MH Humans))) OR (MH "Animal Experimentation+") OR (MH "Models, Animal+") |
|  | S29 | | (((TI animal#) N2 ((TI study) OR (TI model*) OR (TI experiment*))) OR (TI mouse) OR (TI mice) OR (TI rat) OR (TI rats) OR (TI monkey) OR (TI monkeys) OR (TI "preclinical study")) |
|  | S30 | | S28 OR S29 |
| Protocol Question 2-6 | S31 | | ((S3 OR S6 OR S12) AND S27) NOT S30 |
|  | S32 | | S31 AND (LA english) |

Database: CENTRAL

Platform: Cochrane Library

Date of search: April 2, 2025

Limits: English Language; Not animal studies

| Concept | | Line | Search Strategy |
| --- | --- | --- | --- |
| human milk | #1 | | [mh "Milk, Human"] |
|  | #2 | | (((human?:ti,ab,kw OR breast*:ti,ab,kw OR maternal:ti,ab,kw OR mother*:ti,ab,kw) NEXT milk*:ti,ab,kw) OR breastmilk?:ti,ab,kw OR colostrum:ti,ab,kw) |
|  | #3 | | #1 OR #2 |
| infant formula | #4 | | [mh "Infant Formula"] OR [mh "Bottle Feeding"] |
|  | #5 | | (((baby:ti,ab,kw OR babies:ti,ab,kw OR newborn*:ti,ab,kw OR ("new" NEXT born*):ti,ab,kw OR infant*:ti,ab,kw OR neonat*:ti,ab,kw OR ("neo" NEXT nat*):ti,ab,kw) NEXT formula*:ti,ab,kw) OR bottlefe*:ti,ab,kw OR (bottle:ti,ab,kw NEXT (feed*:ti,ab,kw OR fed:ti,ab,kw))) |
|  | #6 | | #4 OR #5 |
| pregnancy | #7 | | [mh Pregnancy] OR [mh "Pregnancy Complications"] OR [mh "Pregnancy Outcome"] |
|  | #8 | | (pregnan*:ti,ab,kw OR prenatal*:ti,ab,kw OR ("pre" NEXT natal*):ti,ab,kw OR postnatal*:ti,ab,kw OR ("post" NEXT natal*):ti,ab,kw OR antenatal*:ti,ab,kw OR ("ante" NEXT natal*):ti,ab,kw OR perinatal*:ti,ab,kw OR ("peri" NEXT natal*):ti,ab,kw OR postpartum:ti,ab,kw OR "post partum":ti,ab,kw OR f?etus*:ti,ab,kw OR f?etal:ti,ab,kw OR maternal:ti,ab,kw OR gravid*:ti,ab,kw OR birth*:ti,ab,kw) |
|  | #9 | | #7 OR #8 |
| lactation | #10 | | [mh Lactation] OR [mh "Breast Feeding"] |
|  | #11 | | (lactat*:ti,ab,kw OR breastfe*:ti,ab,kw OR (breast:ti,ab,kw NEXT (fed:ti,ab,kw OR feed*:ti,ab,kw))) |
|  | #12 | | #10 OR #11 |
| neonates | #13 | | [mh Infant] |
|  | #14 | | ((age?:ti,ab,kw NEAR/2 (0:ti,ab,kw OR 1:ti,ab,kw OR 2:ti,ab,kw OR zero:ti,ab,kw OR one:ti,ab,kw OR two:ti,ab,kw)) OR ((0:ti,ab,kw OR 1:ti,ab,kw OR 2:ti,ab,kw OR zero:ti,ab,kw OR one:ti,ab,kw OR two:ti,ab,kw) NEAR/2 ((year? NEXT "of age"):ti,ab,kw OR "yr or age":ti,ab,kw OR "ys or age":ti,ab,kw OR "yrs or age":ti,ab,kw OR "y or age":ti,ab,kw OR (year? NEXT "old"):ti,ab,kw OR "yr old":ti,ab,kw OR "ys old":ti,ab,kw OR "yrs old":ti,ab,kw OR "y old":ti,ab,kw)) OR ((month?:ti,ab,kw OR mth?:ti,ab,kw OR ms:ti,ab,kw OR m:ti,ab,kw OR week?:ti,ab,kw OR wks:ti,ab,kw OR wk:ti,ab,kw OR w:ti,ab,kw OR day?:ti,ab,kw OR ds:ti,ab,kw OR d:ti,ab,kw) NEAR/2 (old:ti,ab,kw OR "of age":ti,ab,kw)) OR (age?:ti,ab,kw NEAR/3 (month?:ti,ab,kw OR mth?:ti,ab,kw OR week?:ti,ab,kw OR wks:ti,ab,kw OR wk:ti,ab,kw OR day?:ti,ab,kw)) OR infan*:ti,ab,kw OR baby:ti,ab,kw OR babies:ti,ab,kw OR newborn*:ti,ab,kw OR ("new" NEXT born*):ti,ab,kw OR neonat*:ti,ab,kw OR ("neo" NEXT nat*):ti,ab,kw OR preemie?:ti,ab,kw) |
|  | #15 | | #13 OR #14 |
| contaminants | #16 | | [mh Mercury] OR [mh "Mercury Compounds"] OR [mh "Organomercury Compounds"] OR [mh "Mercury Poisoning"] |
|  | #17 | | (mercur*:ti,ab,kw OR methylmercur*:ti,ab,kw OR organomercur*:ti,ab,kw OR MeHg:ti,ab,kw OR CH3Hg:ti,ab,kw OR (Hg:ti,ab,kw AND (metal*:ti,ab,kw OR organometal*:ti,ab,kw OR cation*:ti,ab,kw OR ion*:ti,ab,kw OR isotop*:ti,ab,kw OR radioisotop*:ti,ab,kw OR element*:ti,ab,kw OR microelement*:ti,ab,kw OR organic:ti,ab,kw OR inorganic:ti,ab,kw))) |
|  | #18 | | [mh "Lead Poisoning"] OR [mh Lead] OR [mh "Lead Radioisotopes"] OR [mh "Tetraethyl Lead"] |
|  | #19 | | lead:ti,kw OR (pb:ti,ab,kw OR 208Pb:ti,ab,kw OR organolead*:ti,ab,kw OR plumbum:ti,ab,kw OR plumbic*:ti,ab,kw OR plumbate*:ti,ab,kw OR plumbous:ti,ab,kw OR tetraethyllead:ti,ab,kw OR (lead:ti,ab,kw NEAR/8 (208:ti,ab,kw OR metal*:ti,ab,kw OR organometal*:ti,ab,kw OR cation*:ti,ab,kw OR ion*:ti,ab,kw OR isotop*:ti,ab,kw OR radioisotop*:ti,ab,kw OR element*:ti,ab,kw OR microelement*:ti,ab,kw OR organic:ti,ab,kw OR inorganic:ti,ab,kw OR ore:ti,ab,kw OR blood*:ti,ab,kw OR chemical?:ti,ab,kw OR poison*:ti,ab,kw OR contam*:ti,ab,kw))) |
|  | #20 | | [mh "Arsenic Poisoning"] OR [mh Arsenic] OR [mh Arsenicals] |
|  | #21 | | ((arsen*:ti,ab,kw NOT arsenal:ti,ab,kw) OR arsine?:ti,ab,kw OR organoars*:ti,ab,kw) |
|  | #22 | | [mh Cadmium] OR [mh "Cadmium Poisoning"] OR [mh "Cadmium Radioisotopes"] OR [mh "Cadmium Compounds"] |
|  | #23 | | (cadmi*:ti,ab,kw OR organocadmi*:ti,ab,kw OR (Cd:ti,ab,kw AND (metal*:ti,ab,kw OR organometal*:ti,ab,kw OR cation*:ti,ab,kw OR ion*:ti,ab,kw OR isotop*:ti,ab,kw OR radioisotop*:ti,ab,kw OR element*:ti,ab,kw OR microelement*:ti,ab,kw OR organic:ti,ab,kw OR inorganic:ti,ab,kw))) |
|  | #24 | | [mh Fluorocarbons] |
|  | #25 | | (perfluor*:ti,ab,kw OR polyfluor*:ti,ab,kw OR methylperfluor*:ti,ab,kw OR ethylperfluor*:ti,ab,kw OR fluorocarb*:ti,ab,kw OR fluorochemical*:ti,ab,kw OR fluorosurfactant*:ti,ab,kw OR fluorotelomer*:ti,ab,kw OR fluorinate*:ti,ab,kw OR PFAS?:ti,ab,kw OR PFBA?:ti,ab,kw OR PFBS?:ti,ab,kw OR PFC?:ti,ab,kw OR PFCA?:ti,ab,kw OR PFDA?:ti,ab,kw OR PFDoDA?:ti,ab,kw OR PFDS?:ti,ab,kw OR PFECHS?:ti,ab,kw OR PFESA?:ti,ab,kw OR PFEtCHxS?:ti,ab,kw OR PFHp?:ti,ab,kw OR PFHpA?:ti,ab,kw OR PFHpS?:ti,ab,kw OR PFHxA?:ti,ab,kw OR PFHxS?:ti,ab,kw OR PFNA?:ti,ab,kw OR PFNS?:ti,ab,kw OR PFOA?:ti,ab,kw OR PFOS?:ti,ab,kw OR PFOSA?:ti,ab,kw OR PFPA?:ti,ab,kw OR PFPeA?:ti,ab,kw OR PFPeS?:ti,ab,kw OR PFPiA?:ti,ab,kw OR PFSA?:ti,ab,kw OR PFTE?:ti,ab,kw OR PFTeDA?:ti,ab,kw OR PFTrDA?:ti,ab,kw OR PFUnDA?:ti,ab,kw OR FOSA?:ti,ab,kw OR EtFOSA?:ti,ab,kw OR MeFOSA?:ti,ab,kw OR DONA?:ti,ab,kw OR FTOH?:ti,ab,kw OR FTSA?:ti,ab,kw OR HFPO?:ti,ab,kw OR LCPFA?:ti,ab,kw OR SCPFA?:ti,ab,kw OR Teflon:ti,ab,kw OR freon:ti,ab,kw OR GenX:ti,ab,kw) |
|  | #26 | | [mh ^"Metals, Heavy"] OR [mh ^"Heavy Metal Poisoning"] OR (heavy:ti,ab,kw NEXT metal*:ti,ab,kw) |
|  | #27 | | #16 OR #17 OR #18 OR #19 OR #20 OR #21 OR #22 OR #23 OR #24 OR #25 OR #26 |
| no animal studies | #28 | | ([mh Animals] NOT ([mh Animals] AND [mh ^Humans])) OR [mh "Animal Experimentation"] OR [mh "Models, Animal"] |
|  | #29 | | ((animal?:ti NEAR/2 (study:ti OR model*:ti OR experiment*:ti)) OR mouse:ti OR mice:ti OR rat:ti OR rats:ti OR monkey:ti OR monkeys:ti OR "preclinical study":ti) |
|  | #30 | | #28 OR #29 |
| Protocol | #31 | | ((#3 OR #6 OR #12) AND #27) NOT #30 |
| Question 2-6 | #32 | | #31 AND english:la |

Journal of Environmental Exposure Assessment

Platform: https://www.oaepublish.com/jeea

Date of search: April 21, 2025

Limits: None

| Key Terms Searched in the Journal’s Search Engine, One at a Time | Results |
| --- | --- |
| Human milk | 4 |
| Infant formula | 3 |
| Infant | 5 (all duplicates) |
| Arsenic | 2 |
| Cadmium | 1 |
| Lead | 0 |
| Mercury | 1 |
| PFAS | 2 (1 duplicate) |
| Per- and polyfluoroalkyl substances | 1 (duplicate) |
| Metal | 1 |
| Heavy metals | 0 |
| Breastmilk | 2 (all duplicates) |
| Breast milk | 2 (all duplicates) |
| Baby | 2 (all duplicates) |

## **Supplemental Table 3.** Inclusion/exclusion criteria for a systematic review about the composition/variability of contaminants in human milk and infant formula (PROSPERO: CRD42024528756).

| Criterion | Include | Exclude |
| --- | --- | --- |
| Population | Human milk samples  Infant formula samples (powdered or liquid) | Not human milk or infant formula (e.g., toddler formula) |
| Exposure | Not applicable | Not applicable |
| Comparator | Not applicable | Not applicable |
| Outcome | Composition/variability of contaminants in human milk and infant formula  Contaminants of interest: arsenic (total and inorganic), lead, cadmium, mercury (total and methylmercury), per- and polyfluoroalkyl substances (PFAS) | Components in human milk or infant formula other than: arsenic, lead, cadmium, mercury, PFAS |
| Setting | Articles from countries with a ‘high’ or ‘very high’ level of human development^1^ will be included in an evidence map and articles from the United States only will be included in a qualitative synthesis describing human milk or infant formula | Countries with a medium or low level of human development^1^ |
| Study design | Randomized controlled trials  Non-randomized controlled trials and quasi-experimental studies  Prospective or retrospective cohort studies  Cross-sectional studies  Descriptive studies and other designs without a comparison group | Modeling/simulation studies  Case-control studies  Literature reviews, including systematic reviews and meta-analyses |
| Publication status | Peer-reviewed studies published in scholarly journals | Other content published in scholarly journals (e.g., conference abstracts, letters to the editor, commentaries)  Dissertations/theses  Grey literature |
| Date range | Not applicable | Not applicable |
| Language | English | Other languages |

^1^Human Development Index considers quality of life, education, and standard of living. Available at <https://hdr.undp.org/data-center/human-development-index#/indicies/HDI>.

PFAS, per- and polyfluoroalkyl substances; PROSPERO, International Prospective Register of Systematic Reviews.

## **Supplemental Table 4.** Operationalization of ROBINS-E^1^ for studies assessing contaminants in human milk.

| Domains | ROBINS-E Signaling Question | Operationalization |
| --- | --- | --- |
| Domain 1: Risk of bias due to confounding | No changes; studies were considered at low risk because there were no confounders in the analytical framework (Supplemental Figure 1) identified by the Technical Expert Panel. | |
| Domain 2: Risk of bias arising from measurement of the exposure | No changes; studies were considered at low risk because the exposure was not considered to contribute to bias based on the analytical framework (Supplemental Figure 1) informed by the Technical Expert Panel. | |
| Domain 3: Risk of bias in selection of participants into the study (or into the analysis) | “3.1 Did follow-up begin at (or close to) the start of the exposure window for most participants?”  Guidance states: “For exposures known to have a long latency period (i.e. outcomes that are impacted by the exposure are not expected to occur until after this period), bias is unlikely to be introduced if follow-up starts during the latency period for most participants. In this situation, it is reasonable to answer ‘Yes’ or ‘Probably Yes’ to this question.” | Operationalized as ‘Yes’ or ‘Probably Yes’ for cross-sectional studies because contaminant exposure is likely to have a long latency period. |
|  | “3.3 Was selection of participants into the study (or into the analysis) based on participant characteristics observed after the start of the exposure window being studied?” | Operationalized as the following for cross-sectional studies: Was selection of participants into the study (or into the analysis) based on participant characteristics? |
| Domain 4: Risk of bias due to post-exposure interventions | No change; studies were considered at low risk if they were assessing lifetime contaminant exposure in a general population, in which a reason for intervention would be unlikely. Studies that measured contaminant exposure related to an environmental or poisoning event in which an intervention may be expected before measurement of the outcome were handled on a case-by-case basis. | |
| Domain 5: Risk of bias due to missing data | “5.1 Were complete data on exposure status available for all, or nearly all, participants?”  “5.3 Were complete data on confounding variables available for all, or nearly all, participants?” | Operationalized as ‘Yes’ or ‘Probably Yes’ for all studies because confounding and exposure were not considered as influential sources of bias based on the analytical framework developed by the Technical Expert Panel. |
| Domain 6: Risk of bias arising from measurement of outcomes | No changes. | |
| Domain 7: Risk of bias in selection of the reported result | No changes. | |

^1^Table only includes items that were operationalized in a specific way based on the analytical framework for the research question, informed by the Technical Expert Panel. For full list of domains and signaling questions, please visit: <https://www.riskofbias.info/welcome/robins-e-tool>

Robins-E, Risk of Bias in Non-randomized Studies - of Exposure.

## **Supplemental Table 5.** Risk of bias domains used for studies assessing contaminants in infant formula.

| Sampling Bias/ Generalizability | Missing Data | Outcome | Reporting |
| --- | --- | --- | --- |
| Are the formula samples included in the study representative of formulas available in the US marketplace? This considered how products were selected, as well as locations and time of year. | Were complete data available for all or nearly all contaminants for all or nearly all analyzed samples (formulas)? | Were outcome assessors aware of the type of formula being analyzed?    Could assessment of the outcome have been influenced by knowledge of the type of formula analyzed? | Did the authors report results for all outcomes that they stated they would measure?  Are the results reported likely to be selected based on the basis of desirability of the results from different subgroups (i.e., samples, types, or subgroups of formula)? |
| Domain-level ratings: High, some concerns, or low | Domain-level ratings: High, some concerns, or low | Domain-level ratings: High, some concerns, or low | Domain-level ratings: High, some concerns, or low |
| Overall rating: High, some concerns, or low (based on highest rating across the 4 domains) | | | |

## **Supplemental Table 6.** Full text articles excluded at confirmatory review for human milk with reason.^1^

| Article | Reason for Exclusion |
| --- | --- |
| Keller, Je; Calafat, Antonia M; Kato, Kayoko; Ellefson, Mark E; Reagen, William K; Strynar, Mark; O’Connell, Steven; Butt, Craig M; Mabury, Scott A; Small, Jeff; Muir, Derek C G; Leigh, Stefan D; Schantz, Michele M. Determination of perfluorinated alkyl acid concentrations in human serum and milk standard reference materials. Analytical and bioanalytical chemistry 397 (2010): 439-51 | No outcome |
| Tao, Lin; Ma, Jing; Kunisue, Tatsuya; Libelo, E Laurence; Tanabe, Shinsuke; Kannan, Kurunthachalam. Perfluorinated compounds in human breast milk from several Asian countries, and in infant formula and dairy milk from the United States. Environmental science & technology 42 (2008): 8597-602 | No outcome |
| Kuklenyik, Zsuzsanna; Reich, John Adam; Tully, Jason S; Needham, Larry L; Calafat, Antonia M. Automated solid-phase extraction and measurement of perfluorinated organic acids and amides in human serum and milk. Environmental science & technology 38 (2004): 3698-704 | No outcome |
| Marques RC; Dórea JG; Fonseca MF; Bastos WR; Malm O. Hair mercury in breast-fed infants exposed to thimerosal-preserved vaccines. European journal of pediatrics 166,9 (2007):935-941 | No outcome |
| Laue, Hannah E; Moroishi, Yuka; Palys, Thomas J; Christensen, Brock C; Criswell, Rachel L; Peterson, Lisa A; Huset, Carin A; Baker, Emily R; Karagas, Margaret R; Madan, Juliette C; Romano, Megan E. Early-life exposure to per- and polyfluoroalkyl substances and infant gut microbial composition. Environmental epidemiology (Philadelphia, Pa.) 7 (2023): e238 | Duplicate data |
| Wang, Yuting; Gui, Jiang; Howe, Caitlin G; Emond, Jennifer A; Criswell, Rachel L; Gallagher, Lisa G; Huset, Carin A; Peterson, Lisa A; Botelho, Julianne Cook; Calafat, Antonia M; Christensen, Brock; Karagas, Margaret R; Romano, Megan E. Association of diet with per- and polyfluoroalkyl substances in plasma and human milk in the New Hampshire Birth Cohort Study. The Science of the total environment 933 (2024): 173157 | Duplicate data |
| von Ehrenstein, Ondine S; Fenton, Suzanne E; Kato, Kayoko; Kuklenyik, Zsuzsanna; Calafat, Antonia M; Hines, Erin P. Polyfluoroalkyl chemicals in the serum and milk of breastfeeding women. Reproductive toxicology (Elmsford, N.Y.) 27 (2009): 239-245 | Identified and reviewed during manual search of included articles; database duplicate |
| Zheng, Guomao; Schreder, Erika; Dempsey, Jennifer C; Uding, Nancy; Chu, Valerie; Andres, Gabriel; Sathyanarayana, Sheela; Salamova, Amina. Per- and Polyfluoroalkyl Substances (PFAS) in Breast Milk: Concerning Trends for Current-Use PFAS. Environmental science & technology 55 (2021): 7510-7520 | Identified and reviewed during manual of included articles; database duplicate |
| Murthy, G K; Rhea, U S. Cadmium, copper, iron, lead, manganese, and zinc in evaporated milk, infant products, and human milk. Journal of dairy science 54 (1971): 1001-5 | Identified and reviewed during manual search of included articles; database duplicate |
| Andjelkovic, M.; Van Overmeire, I.; Joly, L.; Poma, G.; Malarvannan, G.; Vleminckx, C.; Malysheva, S. V.; Vanhouche, M.; Van Loco, J.; Van Nieuwenhuyse, A.; Covaci, A. Persistent organic pollutants in human milk of Belgian mothers: levels, time trend and exposure assessment for nursing infants. Journal of Environmental Exposure Assessment 3 (2024): 23 | Identified and reviewed during manual search of Journal of Environmental Exposure Assessment; no outcome |

## **Supplemental Table 7.** Reference list of human milk articles included in heat map.

| Article | Contaminant(s) assessed | Country |
| --- | --- | --- |
| Abafe, Ovokeroye A; Macheka, Linda R; Olowoyo, Joshua O. Confirmatory Analysis of Per and Polyfluoroalkyl Substances in Milk and Infant Formula Using UHPLC-MS/MS. Molecules (Basel, Switzerland). 2021. 26: | Per- and polyfluoroalkyl substances (PFAS) | South Africa |
| Abballe, Annalisa; Ballard, Terri J; Dellatte, Elena; di Domenico, Alessandro; Ferri, Fabiola; Fulgenzi, Anna Rita; Grisanti, Giulio; Iacovella, Nicola; Ingelido, Anna Maria; Malisch, Rainer; Miniero, Roberto; Porpora, Maria Grazia; Risica, Serena; Ziemacki, Gianni; De Felip, Elena. Persistent environmental contaminants in human milk: concentrations and time trends in Italy. Chemosphere. 2008. 73:S220-7 | Lead (Pb), cadmium (Cd), mercury (Hg) | Italy |
| Abdallah, Mohamed Abou-Elwafa; Wemken, Nina; Drage, Daniel Simon; Tlustos, Christina; Cellarius, Claire; Cleere, Kathy; Morrison, John J; Daly, Sean; Coggins, Marie Ann; Harrad, Stuart. Concentrations of perfluoroalkyl substances in human milk from Ireland: Implications for adult and nursing infant exposure. Chemosphere. 2020. 246:125724 | PFAS | Ireland |
| Abdulrazzaq, Yousef M; Osman, Nawal; Nagelkerke, Nicolaas; Kosanovic, Melita; Adem, Abdu. Trace element composition of plasma and breast milk of well-nourished women. Journal of environmental science and health. Part A, Toxic/hazardous substances & environmental engineering. 2008. 43:329-34 | As, Pb, Cd, Hg | United Arab Emirates |
| Alkanani, T.; Friel, J. K.; Jackson, S. E.; Longerich, H. P. Comparison between digestion procedures for the multielemental analysis of milk by inductively coupled plasma mass spectrometry. Journal of Agricultural and Food Chemistry. 1994. 42:1965-1970 | As, Pb, Cd | Canada |
| Almeida, Agostinho A; Lopes, Cristina M P V; Silva, Ana M S; Barrado, Enrique. Trace elements in human milk: correlation with blood levels, inter-element correlations and changes in concentration during the first month of lactation. Journal of trace elements in medicine and biology : organ of the Society for Minerals and Trace Elements (GMS). 2008. 22:196-205 | As, Pb | Portugal |
| Al-Saleh, Iman. Health Risk Assessment of Trace Metals Through Breast Milk Consumption in Saudi Arabia. Biological trace element research. 2021. 199:4535-4545 | Pb, Cd, Hg | Saudi Arabia |
| Al-Saleh, Iman; Abduljabbar, Mai; Al-Rouqi, Reem; Elkhatib, Rola; Alshabbaheen, Ammar; Shinwari, Neptune. Mercury (Hg) exposure in breast-fed infants and their mothers and the evidence of oxidative stress. Biological trace element research. 2013. 153:145-54 | Hg | Saudi Arabia |
| Al-Saleh, Iman; Abduljabbar, Mai; Al-Rouqi, Reem; Eltabache, Chafica; Al-Rajudi, Tahreer; Elkhatib, Rola; Nester, Michael. The extent of mercury (Hg) exposure among Saudi mothers and their respective infants. Environmental monitoring and assessment. 2015. 187:678 | Hg | Saudi Arabia |
| Al-Saleh, Iman; Al-Mohawes, Somiah; Al-Rouqi, Reem; Elkhatib, Rola. Selenium status in lactating mothers-infants and its potential protective role against the neurotoxicity of methylmercury, lead, manganese, and DDT. Environmental research. 2019. 176:108562 | Pb, Hg | Saudi Arabia |
| Al-Saleh, Iman; Elkhatib, Rola; Al-Rouqi, Reem; Abduljabbar, Mai; Eltabache, Chafica; Al-Rajudi, Tahreer; Nester, Michael. Alterations in biochemical markers due to mercury (Hg) exposure and its influence on infant's neurodevelopment. International journal of hygiene and environmental health. 2016. 219:898-914 | Hg | Saudi Arabia |
| Al-Saleh, Iman; Moncari, Lina; Jomaa, Ahmed; Elkhatib, Rola; Al-Rouqi, Reem; Eltabache, Chafica; Al-Rajudi, Tahreer; Alnuwaysir, Hissah; Nester, Michael; Aldhalaan, Hesham. Effects of early and recent mercury and lead exposure on the neurodevelopment of children with elevated mercury and/or developmental delays during lactation: A follow-up study. International journal of hygiene and environmental health. 2020. 230:113629 | Pb, Hg | Saudi Arabia |
| Al-Saleh, Iman; Nester, Michael; Abduljabbar, Mai; Al-Rouqi, Reem; Eltabache, Chafica; Al-Rajudi, Tahreer; Elkhatib, Rola. Mercury (Hg) exposure and its effects on Saudi breastfed infant's neurodevelopment. International journal of hygiene and environmental health. 2016. 219:129-41 | Hg | Saudi Arabia |
| Al-Saleh, Iman; Shinwari, Neptune; Mashhour, Abdullah. Heavy metal concentrations in the breast milk of Saudi women. Biological trace element research. 2003. 96:21-37 | Pb, Cd, Hg | Saudi Arabia |
| Al-sheyab, Nihaya A; Al-Qudah, Khaled M; Tahboub, Yahya R. Levels of perfluorinated compounds in human breast milk in Jordan: the impact of sociodemographic characteristics. Environmental science and pollution research international. 2015. 22:12415-23 | PFAS | Jordan |
| Amarasiriwardena, C. J.; Jayawardene, I.; Lupoli, N.; Barnes, R. M.; Hernandez-Avila, M.; Hu, H.; Ettinger, A. S. Comparison of digestion procedures and methods for quantification of trace lead in breast milk by isotope dilution inductively coupled plasma mass spectrometry. Analytical Methods. 2013. 5:1676-1681 | Pb | Mexico |
| Amiri, Leila; Khalili Doroodzani, Atefeh; Ostovar, Afshin; Dobaradaran, Sina; Mohammadi, Azam; Nabipour, Iraj; Raeisi, Alireza; Malekizadeh, Hasan; Farhadi, Akram; Saeedi, Reza; Afrashteh, Sima; Nazmara, Shahrokh; Keshtkar, Mozhgan. Lactational Exposure of Human Infants to Metal (loid)s: A Comparison of Industrial and Urban Inhabitants in North of the Persian Gulf. Biological trace element research. 2024. 202:1829-1842 | Pb, Hg | Iran (Islamic Republic of) |
| Amziane, Ahmed; Monteau, Fabrice; El Djalil Lalaouna, Abd; Alamir, Barkahom; Le Bizec, Bruno; Dervilly, Gaud. Optimization and validation of a fast supercritical fluid chromatography tandem mass spectrometry method for the quantitative determination of a large set of PFASs in food matrices and human milk. Journal of chromatography. B, Analytical technologies in the biomedical and life sciences. 2022. 1210:123455 | PFAS | Algeria |
| Antignac, Jean-Philippe; Veyrand, Bruno; Kadar, Hanane; Marchand, Philippe; Oleko, Amivi; Le Bizec, Bruno; Vandentorren, Stephanie. Occurrence of perfluorinated alkylated substances in breast milk of French women and relation with socio-demographical and clinical parameters: results of the ELFE pilot study. Chemosphere. 2013. 91:802-8 | PFAS | France |
| Astolfi, Maria Luisa; Marconi, Elisabetta; Protano, Carmela; Vitali, Matteo; Schiavi, Elisa; Mastromarino, Paola; Canepari, Silvia. Optimization and validation of a fast digestion method for the determination of major and trace elements in breast milk by ICP-MS. Analytica chimica acta. 2018. 1040:49-62 | As, Pb, Cd | Italy |
| Astolfi, Maria Luisa; Protano, Carmela; Schiavi, Elisa; Marconi, Elisabetta; Capobianco, Daniela; Massimi, Lorenzo; Ristorini, Martina; Baldassarre, Maria Elisabetta; Laforgia, Nicola; Vitali, Matteo; Canepari, Silvia; Mastromarino, Paola. A prophylactic multi-strain probiotic treatment to reduce the absorption of toxic elements: In-vitro study and biomonitoring of breast milk and infant stools. Environment international. 2019. 130:104818 | As, Pb, Cd, Hg | Italy |
| Aumeistere, Liva; Belusko, Alina; Ciprovica, Inga. Assessment of Heavy Metals and Trace Elements in the Human Milk of Women Living in Latvia and an Evaluation of Influencing Factors. Nutrients. 2024. 16: | As, Pb, Cd | Latvia |
| Awad, Raed; Zhou, Yihui; Nyberg, Elisabeth; Namazkar, Shahla; Yongning, Wu; Xiao, Qianfen; Sun, Yaije; Zhu, Zhiliang; Bergman, Ake; Benskin, Jonathan P. Emerging per- and polyfluoroalkyl substances (PFAS) in human milk from Sweden and China. Environmental science. Processes & impacts. 2020. 22:2023-2030 | PFAS | China, Sweden |
| Bahmani, P.; Sadeghi, S.; Ghahremani, E.; Daraei, H. Evaluation of lead and cadmium levels in breast milk in Sanandaj, Iran. Journal of Advances in Environmental Health Research. 2018. 6:144-151 | Pb, Cd | Iran (Islamic Republic of) |
| Balta, I.; Ola, D.; Filip, R.; Pui, A. M.; Salantai, R.; Cotirta, M.; Tantea, I.; Longodor, A. L.; Marchis, Z.; Coroian, A. Heavy metals and physico-chemical composition of maternal breast milk and colostrum. Scientific Bulletin. Series F. Biotechnologies. 2019. 23:91-101 | Pb | Romania |
| Baluja, G; Hernandez, L M; Gonzalez, M J; Rico, M C. Presence of organochlorine pesticides, polychlorinated biphenyls, and mercury in Spanish human milk samples. Bulletin of environmental contamination and toxicology. 1982. 28:573-7 | Hg | Spain |
| Baranowska, I. The concentration of some trace elements in human milk. Polish Journal of Environmental Studies. 1994. 3:8-May | Pb, Cd | Poland |
| Baranowska-Bosiacka, Irena; Kosinska, Ida; Jamiol, Dominika; Gutowska, Izabela; Prokopowicz, Adam; Rebacz-Maron, Ewa; Goschorska, Marta; Olszowski, Tomasz; Chlubek, Dariusz. Environmental Lead (Pb) Exposure Versus Fatty Acid Content in Blood and Milk of the Mother and in the Blood of Newborn Children. Biological trace element research. 2016. 170:279-87 | Pb | Poland |
| Barbarossa, Andrea; Masetti, Riccardo; Gazzotti, Teresa; Zama, Daniele; Astolfi, Annalisa; Veyrand, Bruno; Pession, Andrea; Pagliuca, Giampiero. Perfluoroalkyl substances in human milk: a first survey in Italy. Environment international. 2013. 51:27-30 | PFAS | Italy |
| Barnett N.W.; Chen L.S.; Kirkbright G.F. Determination of trace concentrations of lead and nickel in freeze-dried human milk by atomic absorption spectrometry and inductively-coupled plasma emission spectrometry. Analytica Chimica Acta. 1983. Vol. 149:115-121 | Pb | United Kingdom |
| Bassil, Maya; Daou, Farah; Hassan, Hussein; Yamani, Osama; Kharma, Joelle Abi; Attieh, Zouheir; Elaridi, Jomana. Lead, cadmium and arsenic in human milk and their socio-demographic and lifestyle determinants in Lebanon. Chemosphere. 2018. 191:911-921 | As, Pb, Cd | Lebanon |
| Bastos, Wanderley R; Vieira, Solange M; Manzatto, Angelo G; Dorea, Jose G; Rubira, Marcelo C; de Souza, Victor Francisco P; da Costa Junior, Walkimar A; Souza Bastos, Maria T. Heterogeneity of Multimedia Exposures to Neurotoxic Elements (Al, As, Cd, Pb, Mn, and Hg) in Breastfed Infants from Porto Velho, Brazil. Biological trace element research. 2018. 184:15-Jul | As, Pb, Cd, Hg | Brazil |
| Behrooz, R. D.; Sari, A. E.; Mishmast-Nehi, A.; Sepehrikia, S.; Barghi, M. Mercury concentration in the milk of mothers living near the southern coast of the Caspian Sea during different stages of lactation period. Toxicological and Environmental Chemistry. 2013. 95:860-869 | Hg | Iran (Islamic Republic of) |
| Behrooz, Reza Dahmardeh; ; Khammar, Sanaz; Rajaei, Fatemeh; Burger, Joanna; Soman, Sidhi; Chakraborty, Paromita. Mercury in saliva, milk, and hair of nursing mothers in southeastern Iranian mothers: levels, distribution and risk assessment. Environmental geochemistry and health. 2024. 46:521 | Hg | Iran (Islamic Republic of) |
| Behrooz, Reza Dahmardeh; Esmaili-Sari, Abbas; Peer, Fatemeh Einollahi; Amini, Malihe. Mercury concentration in the breast milk of Iranian women. Biological trace element research. 2012. 147:36-43 | Hg | Iran (Islamic Republic of) |
| Beser, Maria Isabel; Pardo, Olga; Beltran, Joaquim; Yusa, Vicent. Determination of 21 perfluoroalkyl substances and organophosphorus compounds in breast milk by liquid chromatography coupled to orbitrap high-resolution mass spectrometry. Analytica chimica acta. 2019. 1049:123-132 | PFAS | Spain |
| Beyene, Tesfalidet; Zosky, Graeme R.; Gibson, Peter G.; McDonald, Vanessa M.; Holliday, Elizabeth G.; Horvat, Jay C.; Vertigan, Anne E.; Van Buskirk, Joe; Morgan, Geoffrey G.; Jegasothy, Edward; Hanigan, Ivan; Murphy, Vanessa E.; Jensen, Megan E. The impact of the 2019/2020 Australian landscape fires on infant feeding and contaminants in breast milk in women with asthma. International Breastfeeding Journal. 2023. 18: | Pb | Australia |
| Biego, G. H.; Joyeux, M.; Hartemann, P.; Debry, G. Determination of mineral contents in different kinds of milk and estimation of dietary intake in infants. Food Additives and Contaminants. 1998. 15:775-781 | Pb, Cd | France |
| Bjorklund, Karin Ljung; Vahter, Marie; Palm, Brita; Grander, Margaretha; Lignell, Sanna; Berglund, Marika. Metals and trace element concentrations in breast milk of first time healthy mothers: a biological monitoring study. Environmental health : a global access science source. 2012. 11:92 | As, Pb, Cd | Sweden |
| Bjornberg, Karolin Ask; Vahter, Marie; Berglund, Birgitta; Niklasson, Boel; Blennow, Mats; Sandborgh-Englund, Gunilla. Transport of methylmercury and inorganic mercury to the fetus and breast-fed infant. Environmental health perspectives. 2005. 113:1381-5 | Hg | Sweden |
| Blomberg, Annelise J; Haug, Line S; Lindh, Christian; Sabaredzovic, Azemira; Pineda, Daniela; Jakobsson, Kristina; Nielsen, Christel. Changes in perfluoroalkyl substances (PFAS) concentrations in human milk over the course of lactation: A study in Ronneby mother-child cohort. Environmental research. 2023. 219:115096 | PFAS | Sweden |
| Blomberg, Annelise J; Noren, Erika; Haug, Line S; Lindh, Christian; Sabaredzovic, Azemira; Pineda, Daniela; Jakobsson, Kristina; Nielsen, Christel. Estimated Transfer of Perfluoroalkyl Substances (PFAS) from Maternal Serum to Breast Milk in Women Highly Exposed from Contaminated Drinking Water: A Study in the Ronneby Mother-Child Cohort. Environmental health perspectives. 2023. 131:17005 | PFAS | Sweden |
| Bloom H.; Lewis I.C.; Noller B.N. Elemental analysis of dried milks used in infant feeding. Australian Paediatric Journal. 1978. 14:259-264 | Pb, Cd | Australia |
| Boischio, A A; Henshel, D S. Linear regression models of methyl mercury exposure during prenatal and early postnatal life among riverside people along the upper Madeira river, Amazon. Environmental research. 2000. 83:150-61 | Hg | Brazil |
| Byrne, A. R.; Kosta, L.; Ravnik, V.; Stupar, J.; Hudnik, V. A study of certain trace elements in milk. Nuclear activation techniques in the life sciences 1978. . :255-269 | As, Cd, Hg | Slovenia |
| Bzikowska-Jura, Agnieszka; Wesolowska, Aleksandra; Sobieraj, Piotr; Nawrocka, Agnieszka; Filipek, Aleksandra; Durkalec, Maciej; Katrynska, Danuta; Jedziniak, Piotr. Essential and non-essential element concentrations in human milk samples and the assessment of infants' exposure. Scientific reports. 2024. 14:8140 | As, Pb, Cd, Hg | Poland |
| Cardoso, Osmar O; Juliao, Fabiana C; Alves, Renato I S; Baena, Antonio R; Diez, Isabel G; Suzuki, Meire N; Celere, Beatriz S; Nadal, Marti; Domingo, Jose L; Segura-Munoz, Susana I. Concentration profiles of metals in breast milk, drinking water, and soil: relationship between matrices. Biological trace element research. 2014. 160:116-22 | Pb, Cd, Hg | Brazil |
| Carignan, Courtney C; Cottingham, Kathryn L; Jackson, Brian P; Farzan, Shohreh F; Gandolfi, A Jay; Punshon, Tracy; Folt, Carol L; Karagas, Margaret R. Estimated exposure to arsenic in breastfed and formula-fed infants in a United States cohort. Environmental health perspectives. 2015. 123:500-6 | As | United States |
| Cariou, Ronan; Veyrand, Bruno; Yamada, Ami; Berrebi, Alain; Zalko, Daniel; Durand, Sophie; Pollono, Charles; Marchand, Philippe; Leblanc, Jean-Charles; Antignac, Jean-Philippe; Le Bizec, Bruno. Perfluoroalkyl acid (PFAA) levels and profiles in breast milk, maternal and cord serum of French women and their newborns. Environment international. 2015. 84:71-81 | PFAS | France |
| Casey, C E. The content of some trace elements in infant milk foods and supplements available in New Zealand. The New Zealand medical journal. 1977. 85:275-8 | Pb, Cd | New Zealand |
| Castillo-Castaneda, Patricia Carolina; Gaxiola-Robles, Ramon; Labrada-Martagon, Vanessa; Acosta Vargas, Baudilio; Mendez-Rodriguez, Lia Celina; Zenteno-Savin, Tania. Oxidative damage to proteins related to metals and antioxidant defenses in breastmilk. Nutricion hospitalaria. 2017. 34:59-64 | Pb, Cd | Mexico |
| Castriotta, Luigi; Rosolen, Valentina; Biggeri, Annibale; Ronfani, Luca; Catelan, Dolores; Mariuz, Marika; Bin, Maura; Brumatti, Liza Vecchi; Horvat, Milena; Barbone, Fabio. The role of mercury, selenium and the Se-Hg antagonism on cognitive neurodevelopment: A 40-month follow-up of the Italian mother-child PHIME cohort. International journal of hygiene and environmental health. 2020. 230:113604 | Hg | Italy |
| Castro, Francisca; Harari, Florencia; Llanos, Miguel; Vahter, Marie; Ronco, Ana Maria. Maternal-child transfer of essential and toxic elements through breast milk in a mine-waste polluted area. American journal of perinatology. 2014. 31:993-1002 | As, Pb, Cd | Chile |
| Cebi, Aysegul; Sengul, Umit. Toxic metal and trace element status in the breast milk of Turkish new-born mothers. Journal of trace elements in medicine and biology : organ of the Society for Minerals and Trace Elements (GMS). 2022. 74:127066 | As, Pb, Cd | Turkiye |
| Cechova, Eliska; Scheringer, Martin; Seifertova, Marta; Mikes, Ondrej; Kroupova, Kristyna; Kuta, Jan; Forns, Joan; Eggesbo, Merete; Quaak, Ilona; de Cock, Marijke; van de Bor, Margot; Patayova, Henrieta; Palkovicova Murinova, Lubica; Kocan, Anton. Developmental neurotoxicants in human milk: Comparison of levels and intakes in three European countries. The Science of the total environment. 2017. 579:637-645 | Hg | Netherlands, Norway, Slovakia |
| Cerbino, M R; Vieira, Jose Cavalcante Souza; Braga, C P; Oliveira, G; Padilha, I F; Silva, T M; Zara, L F; Silva, N J Jr; Padilha, P M. Metalloproteomics Approach to Analyze Mercury in Breast Milk and Hair Samples of Lactating Women in Communities of the Amazon Basin, Brazil. Biological trace element research. 2018. 181:216-226 | Hg | Brazil |
| Cerna, Milena; Grafnetterova, Anna Pinkr; Dvorakova, Darina; Pulkrabova, Jana; Maly, Marek; Janos, Tomas; Vodrazkova, Nicole; Tupa, Zdenka; Puklova, Vladimira. Biomonitoring of PFOA, PFOS and PFNA in human milk from Czech Republic, time trends and estimation of infant's daily intake. Environmental research. 2020. 188:109763 | PFAS | Czechia |
| Chen, Chen; Ma, Chaochen; Li, Qiyao; Hang, Jin Guo; Shen, Jiantong; Nakayama, Shoji F; Kido, Teruhiko; Lin, Yibin; Feng, Hao; Jung, Chau-Ren; Sun, Xian Liang; Lou, Jianlin. Prenatal Exposure to Heavy Metals and Adverse Birth Outcomes: Evidence From an E-Waste Area in China. GeoHealth. 2023. 7:e2023GH000897 | As, Pb, Cd | China |
| Chi, Zhi Hao; ; Liu, Lan; Zheng, Jingyun; Tian, Lei; Chevrier, Jonathan; Bornman, Riana; Obida, Muvhulawa; Gates Goodyer, Cindy; Hales, Barbara F; Bayen, Stephane. Investigation of common and unreported parabens alongside other plastic-related contaminants in human milk using non-targeted strategies. Chemosphere. 2025. 373:144154 | PFAS | Canada, South Africa |
| Chu, T. C.; Navarrete, V. R.; Kaji, H.; Izawa, G.; Shiokawa, T.; Ishii, K.; Morita, S.; Tawara, H. A study on proton induced X-ray analysis and its application to environmental samples. Journal of Radioanalytical Chemistry. 1977. 36:195-207 | Pb | Japan |
| Cinar, Nursan; Ozdemir, Sami; Yucel, Oya; Ucar, Fatma. In which regions is breast-feeding safer from the impact of toxic elements from the environment?. Bosnian journal of basic medical sciences. 2011. 11:234-9 | Pb, Cd | Turkiye |
| Concha, G; Vogler, G; Lezcano, D; Nermell, B; Vahter, M. Exposure to inorganic arsenic metabolites during early human development. Toxicological sciences : an official journal of the Society of Toxicology. 1998. 44:185-90 | As | Argentina |
| Concha, G; Vogler, G; Nermell, B; Vahter, M. Low-level arsenic excretion in breast milk of native Andean women exposed to high levels of arsenic in the drinking water. International archives of occupational and environmental health. 1998. 71:42-6 | As | Argentina |
| Coni, E.; Stacchini, A.; Caroli, S.; Falconieri, P. Analytical approach to obtaining reference values for minor and trace elements in human milk. Journal of Analytical Atomic Spectrometry. 1990. 5:581-586 | Pb, Cd | Italy |
| Coni, E; Falconieri, P; Ferrante, E; Semeraro, P; Beccaloni, E; Stacchini, A; Caroli, S. Reference values for essential and toxic elements in human milk. Annali dell'Istituto superiore di sanita. 1990. 26:119-30 | Pb, Cd | Italy |
| Counter, S Allen; Buchanan, Leo H; Ortega, Fernando; Chiriboga, Roberto; Correa, Rommy; Collaguaso, Maria Angela. Lead levels in the breast milk of nursing andean mothers living in a lead-contaminated environment. Journal of toxicology and environmental health. Part A. 2014. 77:993-1003 | As, Pb, Cd, Hg | Ecuador |
| Covaciu, F. D.; Feher, I.; Molnar, C.; Floare-Avram, V.; Dehelean, A. Characterization of the fatty acid and elemental composition of human milk with chemometric processing to determine the nutritional value. Analytical Letters. 2023. 56:344-356 | As, Pb, Cd | Romania |
| Criswell, Rachel L; Wang, Yuting; Christensen, Brock; Botelho, Julianne C; Calafat, Antonia M; Peterson, Lisa A; Huset, Carin A; Karagas, Margaret R; Romano, Megan E. Concentrations of Per- and Polyfluoroalkyl Substances in Paired Maternal Plasma and Human Milk in the New Hampshire Birth Cohort. Environmental science & technology. 2023. 57:463-472 | PFAS | United States |
| Cunha, Leandro Rodrigues da; Costa, Teresa Helena Macedo da; Caldas, Eloisa Dutra. Mercury concentration in breast milk and infant exposure assessment during the first 90 days of lactation in a midwestern region of Brazil. Biological trace element research. 2013. 151:30-7 | Hg | Brazil |
| da Costa, Sergio L; Malm, Olaf; Dorea, Jose G. Breast-milk mercury concentrations and amalgam surface in mothers from Brasilia, Brazil. Biological trace element research. 2005. 106:145-51 | Hg | Brazil |
| Dabeka, R W; Karpinski, K F; McKenzie, A D; Bajdik, C D. Survey of lead, cadmium and fluoride in human milk and correlation of levels with environmental and food factors. Food and chemical toxicology : an international journal published for the British Industrial Biological Research Association. 1986. 24:913-21 | Pb, Cd | Canada |
| Dabeka, R W; McKenzie, A D. Lead and cadmium levels in commercial infant foods and dietary intake by infants 0-1 year old. Food additives and contaminants. 1988. 5:333-42 | Pb, Cd | Canada |
| Dalili H.; Shariat M.; Kavyani Z.; Fazel M.; Raji F.; Jamali F.; Ghorban-Sabagh V. Correlation between lead in maternal blood, umbilical cord blood, and breast milk with newborn anthropometric characteristics. Iranian Journal of Neonatology. 2019. 10:11-Jun | Pb | Iran (Islamic Republic of) |
| Dargahi, Abdollah; ; Zandian, Hamed; Samiee, Fateme; Farzipour, Shahla; Sheikhmemari, Negin; Kazemipour-Khabbazi, Salva. Assessment of toxic metals and trace elements in breast milk of mothers and their health risks to infants: A cross-sectional study in Ardabil, Northwest Iran. Food and chemical toxicology : an international journal published for the British Industrial Biological Research Association. 2025. 200:115389 | As, Pb, Cd, Hg | Iran (Islamic Republic of) |
| De Felip, Elena; Bianchi, Fabrizio; Bove, Crescenzo; Cori, Liliana; D'Argenzio, Angelo; D'Orsi, Giancarlo; Fusco, Mario; Miniero, Roberto; Ortolani, Rosanna; Palombino, Raffaele; Parlato, Antonino; Pelliccia, Maria Grazia; Peluso, Filomena; Piscopo, Giovanni; Pizzuti, Renato; Porpora, Maria Grazia; Protano, Domenico; Senofonte, Oreste; Spena, Silvana Russo; Simonetti, Andrea; di Domenico, Alessandro. Priority persistent contaminants in people dwelling in critical areas of Campania Region, Italy (SEBIOREC biomonitoring study). The Science of the total environment. 2014. 487:420-35 | As, Pb, Cd, Hg | Italy |
| Deroma, L; Parpinel, M; Tognin, V; Channoufi, L; Tratnik, J; Horvat, M; Valent, F; Barbone, F. Neuropsychological assessment at school-age and prenatal low-level exposure to mercury through fish consumption in an Italian birth cohort living near a contaminated site. International journal of hygiene and environmental health. 2013. 216:486-93 | Hg | Italy |
| Desalegn, Anteneh Assefa; van der Ent, Wietske; Lenters, Virissa; Iszatt, Nina; Stigum, Hein; Lyche, Jan Ludvig; Berg, Vidar; Kirstein-Smardzewska, Karolina J; Esguerra, Camila Vicencio; Eggesbo, Merete. Perinatal exposure to potential endocrine disrupting chemicals and autism spectrum disorder: From Norwegian birth cohort to zebrafish studies. Environment international. 2023. 181:108271 | PFAS | Norway |
| Dillon, H K; Wilson, D J; Schaffner, W. Lead concentrations in human milk. American journal of diseases of children (1960). 1974. 128:491-2 | Pb | United States |
| dos Santos, Felipe Andre; Cavecci, Bruna; Vieira, Jose Cavalcante Souza; Franzini, Vanessa Pezza; Santos, Ademir; de Lima Leite, Aline; Buzalaf, Marilia Afonso Rabelo; Zara, Luiz Fabricio; de Magalhaes Padilha, Pedro. A Metalloproteomics Study on the Association of Mercury With Breast Milk in Samples From Lactating Women in the Amazon Region of Brazil. Archives of environmental contamination and toxicology. 2015. 69:223-9 | Hg | Brazil |
| Drasch, G; Aigner, S; Roider, G; Staiger, F; Lipowsky, G. Mercury in human colostrum and early breast milk. Its dependence on dental amalgam and other factors. Journal of trace elements in medicine and biology : organ of the Society for Minerals and Trace Elements (GMS). 1998. 12:23-7 | Hg | Germany |
| Drexler, H; Schaller, K H. The mercury concentration in breast milk resulting from amalgam fillings and dietary habits. Environmental research. 1998. 77:124-9 | Hg | Germany |
| Durrant S.F.; Ward N.I. Multi-elemental analysis of human milk by inductively coupled plasma-source mass spectrometry: Implications for infant health. Journal of Micronutrient Analysis. 1989. 5:111-126 | As, Pb, Cd, Hg | United Kingdom |
| Dursun, Arzu; Yurdakok, Kadriye; Yalcin, Songul S; Tekinalp, Gulsevin; Aykut, Osman; Orhan, Gunnur; Morgil, Goksel Koc. Maternal risk factors associated with lead, mercury and cadmium levels in umbilical cord blood, breast milk and newborn hair. The journal of maternal-fetal & neonatal medicine : the official journal of the European Association of Perinatal Medicine, the Federation of Asia and Oceania Perinatal Societies, the International Society of Perinatal Obstetricians. 2016. 29:954-61 | Pb, Cd, Hg | Turkiye |
| Ecsedi-Angyal, M.; Tatar, E.; Ovari, M.; Kurin-Csorgei, K.; Zaray, G.; Mihucz, V. G. Determination of low-level arsenic, lead, cadmium and mercury concentration in breast milk of Hungarian women. International Journal of Environmental Analytical Chemistry. 2020. 100:549-566 | As, Pb, Cd, Hg | Hungary |
| Eick, Stephanie M; ; Sehgal, Neha; Salamova, Amina; Fiedler, Nancy; Hood, Robert B; Yakimavets, Volha; Promkam, Nattawadee; Prapamontol, Tippawan; Suttiwan, Panrapee; Sittiwang, Supattra; Mangklabruks, Ampica; Naksen, Warangkana; Panuwet, Parinya; Barr, Dana Boyd. Per- and polyfluoroalkyl substances in paired serum and breastmilk samples among pregnant farmworkers in Thailand. International journal of hygiene and environmental health. 2025. 264:114509 | PFAS | Thailand |
| Enyoh, Christian Ebere; Ovuoraye, Prosper E; Qingyue, Wang; Wang, Weiqian. Examining the impact of nanoplastics and PFAS exposure on immune functions through inhibition of secretory immunoglobin A in human breast milk. Journal of hazardous materials. 2023. 459:132103 | PFAS | Japan |
| Ernawati; Sutomo A.H.; Indwiani A.A. Overview of 8-hydroxy-2'-deoxyguanosine (8-ohdg) as DNA damage biomarker in infants who live near gold mines, Yogyakarta, Indonesia. Indian Journal of Public Health Research and Development. 2021. 12:101-105 | Hg | Indonesia |
| Ettinger, Adrienne S; Tellez-Rojo, Martha Maria; Amarasiriwardena, Chitra; Gonzalez-Cossio, Teresa; Peterson, Karen E; Aro, Antonio; Hu, Howard; Hernandez-Avila, Mauricio. Levels of lead in breast milk and their relation to maternal blood and bone lead levels at one month postpartum. Environmental health perspectives. 2004. 112:926-31 | Pb | Mexico |
| Ettinger, Adrienne S; Tellez-Rojo, Martha Maria; Amarasiriwardena, Chitra; Peterson, Karen E; Schwartz, Joel; Aro, Antonio; Hu, Howard; Hernandez-Avila, Mauricio. Influence of maternal bone lead burden and calcium intake on levels of lead in breast milk over the course of lactation. American journal of epidemiology. 2006. 163:48-56 | Pb | Mexico |
| Fernando, N. P.; Shalltout, A.; Adel, S. How do we cope with lactation and the mother-infant dyad exposed to lead?. Refrigeration Science and Technology. 1981. :185-187 | Pb | Kuwait |
| Fiedler, H.; Sadia, M.; Krauss, T.; Baabish, A.; Yeung, L. W. Y. Perfluoroalkane acids in human milk under the global monitoring plan of the Stockholm convention on persistent organic pollutants (2008-2019). Frontiers of Environmental Science and Engineering. 2022. 16: | PFAS | Brazil |
| Fiedler, Heidelore; Sadia, Mohammad. Regional occurrence of perfluoroalkane substances in human milk for the global monitoring plan under the Stockholm Convention on Persistent Organic Pollutants during 2016-2019. Chemosphere. 2021. 277:130287 | PFAS | Sweden |
| Filho H.J.I.; Baccan N.; Kus M.M.M.; Soares V.A.; Salazar R.F.S.; Alcantara M.A.K.; Nascimento L.F.C. Determination of essential and toxic elements in maternal milk collected in Taubate, Sao Paulo State, Brazil. Journal of Food, Agriculture and Environment. 2014. 12:88-92 | Pb, Cd | Brazil |
| Forns, J; Iszatt, N; White, R A; Mandal, S; Sabaredzovic, A; Lamoree, M; Thomsen, C; Haug, L S; Stigum, H; Eggesbo, M. Perfluoroalkyl substances measured in breast milk and child neuropsychological development in a Norwegian birth cohort study. Environment international. 2015. 83:176-82 | PFAS | Norway |
| Freire, Carmen; Iribarne-Duran, Luz Maria; Gil, Fernando; Olmedo, Pablo; Serrano-Lopez, Laura; Pena-Caballero, Manuela; Hurtado, Jose-Antonio; Alvarado-Gonzalez, Nelva E; Fernandez, Mariana F; Peinado, Francisco M; Artacho-Cordon, Francisco; Olea, Nicolas. Concentrations and determinants of lead, mercury, cadmium, and arsenic in pooled donor breast milk in Spain. International journal of hygiene and environmental health. 2022. 240:113914 | As, Pb, Cd, Hg | Spain |
| Friel, J K; Andrews, W L; Jackson, S E; Longerich, H P; Mercer, C; McDonald, A; Dawson, B; Sutradhar, B. Elemental composition of human milk from mothers of premature and full-term infants during the first 3 months of lactation. Biological trace element research. 1999. 67:225-47 | Pb, Cd | Canada |
| Fromme, Hermann; Fuchs, Veronika; Albrecht, Michael; Aschenbrenner, Bettina; Rohl, Claudia; Janitzki, Nora; Herber-Jonat, Susanne; Wockner, Mandy; Volkel, Wolfgang; Flemmer, Andreas W; Schober, Wolfgang. Polychlorinated dioxins and dibenzofurans (PCDD/F), polybrominated dioxins and dibenzofurans (PBDD/F), polychlorinated biphenyls (PCB), polybrominated diphenyl ethers (PBDE), and per- and polyfluoroalkyl substances (PFAS) in German breast milk samples (LU. The Science of the total environment. 2022. 825:154066 | PFAS | Germany |
| Fromme, Hermann; Mosch, Christine; Morovitz, Maria; Alba-Alejandre, Irene; Boehmer, Sigrun; Kiranoglu, Mandy; Faber, Fabienne; Hannibal, Iris; Genzel-Boroviczeny, Orsolya; Koletzko, Berthold; Volkel, Wolfgang. Pre- and postnatal exposure to perfluorinated compounds (PFCs). Environmental science & technology. 2010. 44:7123-9 | PFAS | Germany |
| Fujii, Yukiko; Yan, Junxia; Harada, Kouji H; Hitomi, Toshiaki; Yang, Hyeran; Wang, Peiyu; Koizumi, Akio. Levels and profiles of long-chain perfluorinated carboxylic acids in human breast milk and infant formulas in East Asia. Chemosphere. 2012. 86:315-21 | PFAS | China, Japan, Korea (Republic of) |
| Fujita, M; Takabatake, E. Mercury levels in human maternal and neonatal blood, hair and milk. Bulletin of environmental contamination and toxicology. 1977. 18:205-9 | Hg | Japan |
| Galster, W A. Mercury in Alaskan Eskimo mothers and infants. Environmental health perspectives. 1976. 15:135-40 | Hg | United States |
| Garcia Salcedo, Jose Javier; Roh, Taehyun; Nava Rivera, Lydia Enith; Betancourt Martinez, Nadia Denys; Carranza Rosales, Pilar; San Miguel Salazar, Maria Francisco; Rivera Guillen, Mario Alberto; Serrano Gallardo, Luis Benjamin; Nino Castaneda, Maria Sonadora; Guzman Delgado, Nacny Elena; Millan Orozco, Jair; Ortega Morales, Natalia; Moran Martinez, Javier. Comparative Biomonitoring of Arsenic Exposure in Mothers and Their Neonates in Comarca Lagunera, Mexico. International journal of environmental research and public health. 2022. 19: | As | Mexico |
| Garcia-Esquinas, Esther; Perez-Gomez, Beatriz; Fernandez, Mario Antonio; Perez-Meixeira, Ana Maria; Gil, Elisa; de Paz, Concha; Iriso, Andres; Sanz, Juan Carlos; Astray, Jenaro; Cisneros, Margot; de Santos, Amparo; Asensio, Angel; Garcia-Sagredo, Jose Miguel; Garcia, Jose Frutos; Vioque, Jesus; Pollan, Marina; Lopez-Abente, Gonzalo; Gonzalez, Maria Jose; Martinez, Mercedes; Bohigas, Pedro Arias; Pastor, Roberto; Aragones, Nuria. Mercury, lead and cadmium in human milk in relation to diet, lifestyle habits and sociodemographic variables in Madrid (Spain). Chemosphere. 2011. 85:268-76 | Pb, Cd, Hg | Spain |
| Gaxiola-Robles R.; Labrada-Martagon V.; Mendez-Rodriguez L.C.; Olguin-Monroy N.O.; Castillo-Castaneda P.C.; Zenteno-Savin T. Interaction between Selenium (Se) and Mercury (Hg) affects the activity of glutathione S-transferase in breast milk; possible relationship with fish and shellfish intake. Free Radical Biology and Medicine. 2013. 65:S112 | As, Hg | Mexico |
| Gaxiola-Robles, Ramon; Labrada-Martagon, Vanessa; Bitzer-Quintero, Oscar Kurt; Zenteno-Savin, Tania; Mendez-Rodriguez, Lia Celina. Purine nucleoside phosphorylase and the enzymatic antioxidant defense system in breast milk from women with different levels of arsenic exposure. Nutricion hospitalaria. 2015. 31:2289-96 | As | Mexico |
| Gaxiola-Robles, Ramon; Labrada-Martagon, Vanessa; Celis de la Rosa, Alfredo de Jesus; Acosta-Vargas, Baudilio; Mendez-Rodriguez, Lia Celina; Zenteno-Savin, Tania. Interaction between mercury (Hg), arsenic (As) and selenium (Se) affects the activity of glutathione S-transferase in breast milk; possible relationship with fish and sellfish intake. Nutricion hospitalaria. 2014. 30:436-46 | As, Hg | Mexico |
| Goudarzi, M A; Parsaei, P; Nayebpour, F; Rahimi, E. Determination of mercury, cadmium and lead in human milk in Iran. Toxicology and industrial health. 2013. 29:820-3 | Pb, Cd, Hg | Iran (Islamic Republic of) |
| Grandjean, P; Weihe, P; Needham, L L; Burse, V W; Patterson, D G Jr; Sampson, E J; Jorgensen, P J; Vahter, M. Relation of a seafood diet to mercury, selenium, arsenic, and polychlorinated biphenyl and other organochlorine concentrations in human milk. Environmental research. 1995. 71:29-38 | As, Hg | Denmark |
| Grzunov Letinic, Judita; Matek Saric, Marijana; Piasek, Martina; Jurasovic, Jasna; Varnai, Veda Marija; Sulimanec Grgec, Antonija; Orct, Tatjana. Use of human milk in the assessment of toxic metal exposure and essential element status in breastfeeding women and their infants in coastal Croatia. Journal of trace elements in medicine and biology : organ of the Society for Minerals and Trace Elements (GMS). 2016. 38:117-125 | Pb, Cd, Hg | Croatia |
| Guerranti, Cristiana; Perra, Guido; Corsolini, Simonetta; Focardi, Silvano E. Pilot study on levels of perfluorooctane sulfonic acid (PFOS) and perfluorooctanoic acid (PFOA) in selected foodstuffs and human milk from Italy. Food chemistry. 2013. 140:197-203 | PFAS | Italy |
| Gulson, B L; Jameson, C W; Mahaffey, K R; Mizon, K J; Patison, N; Law, A J; Korsch, M J; Salter, M A. Relationships of lead in breast milk to lead in blood, urine, and diet of the infant and mother. Environmental health perspectives. 1998. 106:667-74 | Pb | Australia |
| Gulson, B L; Mizon, K J; Korsch, M J; Mahaffey, K R; Taylor, A J. Dietary intakes of selected elements from longitudinal 6-day duplicate diets for pregnant and nonpregnant subjects and elemental concentrations of breast milk and infant formula. Environmental research. 2001. 87:160-74 | Pb | Australia |
| Gulson, B L; Mizon, K J; Palmer, J M; Patison, N; Law, A J; Korsch, M J; Mahaffey, K R; Donnelly, J B. Longitudinal study of daily intake and excretion of lead in newly born infants. Environmental research. 2001. 85:232-45 | Pb | Australia |
| Gundacker, C; Pietschnig, B; Wittmann, K J; Lischka, A. Human milk mercury (Hg) and lead (Pb) levels in Vienna. Advances in experimental medicine and biology. 2000. 478:387-8 | Pb, Hg | Austria |
| Gundacker, Claudia; Frohlich, Sonja; Graf-Rohrmeister, Klaudia; Eibenberger, Barbara; Jessenig, Verena; Gicic, Dijana; Prinz, Susanne; Wittmann, Karl Johann; Zeisler, Harald; Vallant, Birgit; Pollak, Arnold; Husslein, Peter. Perinatal lead and mercury exposure in Austria. The Science of the total environment. 2010. 408:5744-9 | Pb, Hg | Austria |
| Gundacker, Claudia; Pietschnig, Beate; Wittmann, Karl J; Lischka, Andreas; Salzer, Hans; Hohenauer, Leonhard; Schuster, Ernst. Lead and mercury in breast milk. Pediatrics. 2002. 110:873-8 | Pb, Hg | Austria |
| Gundacker, Claudia; Pietschnig, Beate; Wittmann, Karl J; Salzer, Hans; Stoger, Helmut; Reimann-Dorninger, Gerda; Schuster, Ernst; Lischka, Andreas. Smoking, cereal consumption, and supplementation affect cadmium content in breast milk. Journal of exposure science & environmental epidemiology. 2007. 17:39-46 | Cd | Austria |
| Gurbay, Aylin; Charehsaz, Mohammad; Eken, Ayse; Sayal, Ahmet; Girgin, Gozde; Yurdakok, Murat; Yigit, Sule; Erol, Dilek Demir; Sahin, Gonul; Aydin, Ahmet. Toxic metals in breast milk samples from Ankara, Turkey: assessment of lead, cadmium, nickel, and arsenic levels. Biological trace element research. 2012. 149:117-22 | As, Pb, Cd | Turkiye |
| Hall, Amber M; ; Ashley-Martin, Jillian; Lei Liang, Chun; Papandonatos, George D; Arbuckle, Tye E; Borghese, Michael M; Buckley, Jessie P; Cecil, Kim M; Chen, Aimin; Dodds, Linda; Fisher, Mandy; Lanphear, Bruce P; Fk Rawn, Dorothea; Yolton, Kimberly; Braun, Joseph M. Personal care product use and per- and polyfluoroalkyl substances in pregnant and lactating people in the Maternal-Infant Research on Environmental Chemicals study. Environment international. 2024. 193:109094 | PFAS | Canada |
| Hallen, I P; Jorhem, L; Lagerkvist, B J; Oskarsson, A. Lead and cadmium levels in human milk and blood. The Science of the total environment. 1995. 166:149-55 | Pb, Cd | Sweden |
| Hamzaoglu, Onur; Yavuz, Melike; Turker, Gulcan; Savli, Hakan. Air Pollution and Heavy Metal Concentration in Colostrum and Meconium in Two Different Districts of an Industrial City: A Preliminary Report. International Medical Journal. 2014. 21:77-82 | As, Pb, Cd, Hg | Turkiye |
| Han, Feng; Liu, Jiaying; Wang, Yuxin; Li, Jingguang; Lyu, Bing; Zhao, Yunfeng; Wu, Yongning. Penetration of Perfluorooctanesulfonate Isomers and Their Alternatives from Maternal Blood to Milk and Its Associations with Chemical Properties and Milk Primary Components. Environmental science & technology. 2023. 57:2457-2463 | PFAS | China |
| Han, Feng; Wang, Yuxin; Li, Jingguang; Lyu, Bing; Liu, Jiaying; Zhang, Jian; Zhao, Yunfeng; Wu, Yongning. Occurrences of legacy and emerging per- and polyfluoroalkyl substances in human milk in China: Results of the third National Human Milk Survey (2017-2020). Journal of hazardous materials. 2023. 443:130163 | PFAS | China |
| Hang, Jin Guo; Dong, Jing Jian; Feng, Hao; Huang, Jian Zhong; Wang, Zheng; Shen, Bin; Nakayama, Shoji F; Kido, Teruhiko; Jung, Chau-Ren; Ma, Chaochen; Sun, Xian Liang. Evaluating postnatal exposure to six heavy metals in a Chinese e-waste recycling area. Chemosphere. 2022. 308:136444 | As, Pb, Cd | China |
| Hanning, Rhona M; Sandhu, Ranjit; MacMillan, Angus; Moss, Lorraine; Tsuji, Leonard J S; Nieboer, Evert. Impact on blood Pb levels of maternal and early infant feeding practices of First Nation Cree in the Mushkegowuk Territory of northern Ontario, Canada. Journal of environmental monitoring : JEM. 2003. 5:241-5 | Pb | Canada |
| Hartmann, Christina; Kaiser, Andreas-Marius; Moche, Wolfgang; Weiss, Stefan; Raffesberg, Wolfgang; Scharf, Sigrid; Graf-Rohrmeister, Klaudia; Thanhaeuser, Margarita; Haiden, Nadja; Uhl, Maria. Persistent Organic Pollutants in Austrian Human Breast Milk Collected between 2013 and 2016. Journal of xenobiotics. 2024. 14:247-266 | PFAS | Austria |
| Hassan, Hussein F; Bou Ghanem, Haneen; Abi Kharma, Joelle; Abiad, Mohamad G; Elaridi, Jomana; Bassil, Maya. Perfluorooctanoic Acid and Perfluorooctane Sulfonate in Human Milk: First Survey from Lebanon. International journal of environmental research and public health. 2023. 20: | PFAS | Lebanon |
| Honda, Ryumon; Tawara, Kenji; Nishijo, Muneko; Nakagawa, Hideaki; Tanebe, Kyoko; Saito, Shigeru. Cadmium exposure and trace elements in human breast milk. Toxicology. 2003. 186:255-9 | Cd | Japan |
| Iszatt, Nina; Janssen, Stefan; Lenters, Virissa; Dahl, Cecilie; Stigum, Hein; Knight, Rob; Mandal, Siddhartha; Peddada, Shyamal; Gonzalez, Antonio; Midtvedt, Tore; Eggesbo, Merete. Environmental toxicants in breast milk of Norwegian mothers and gut bacteria composition and metabolites in their infants at 1 month. Microbiome. 2019. 7:34 | PFAS | Norway |
| Iwai, Kenta; Iwai-Shimada, Miyuki; Asato, Kaname; Nakai, Kunihiko; Kobayashi, Yayoi; Nakayama, Shoji F; Tatsuta, Nozomi. Intra- and Inter-Day Element Variability in Human Breast Milk: Pilot Study. Toxics. 2022. 10: | As, Pb, Cd, Hg | Japan |
| Iwai-Shimada, Miyuki; Satoh, Hiroshi; Nakai, Kunihiko; Tatsuta, Nozomi; Murata, Katsuyuki; Akagi, Hirokatsu. Methylmercury in the breast milk of Japanese mothers and lactational exposure of their infants. Chemosphere. 2015. 126:67-72 | Hg | Japan |
| Iyengar, G V; Kasperek, K; Feinendegen, L E; Wang, Y X; Weese, H. Determination of Co, Cu, Fe, Hg, Mn, Sb, Se and Zn in milk samples. The Science of the total environment. 1982. 24:267-74 | Hg | Multiple, not described |
| Iyengar, G. V. Radiochemical separations for inorganic trace elements in some biological reference materials, foods, tissues and body-fluids. Journal Radioanalytical and Nuclear Chemistry. 1987. 110:503-517 | Cd | Austria, Germany |
| Iyengar, G. V. Trace elements in human milk: results from a 6 nation survey. Zeitschrift fur die Gesamte Hygiene und ihre Grenzgebiete. 1984. 30:88 | Hg | Hungary, Sweden |
| Jagodic, Marta; Potocnik, Doris; Snoj Tratnik, Janja; Mazej, Darja; Pavlin, Majda; Trdin, Ajda; Eftimov, Tome; Kononenko, Lijana; Ogrinc, Nives; Horvat, Milena. Selected elements and fatty acid composition in human milk as indicators of seafood dietary habits. Environmental research. 2020. 180:108820 | As, Pb, Cd, Hg | Slovenia |
| Jin, Hangbiao; Mao, Lingling; Xie, Jiahui; Zhao, Meirong; Bai, Xiaoxia; Wen, Jie; Shen, Tao; Wu, Pengfei. Poly- and perfluoroalkyl substance concentrations in human breast milk and their associations with postnatal infant growth. The Science of the total environment. 2020. 713:136417 | PFAS | China |
| Jusko, Todd A; Oktapodas, Marina; Palkovicova Murinova, L'ubica; Babinska, Katarina; Babjakova, Jana; Verner, Marc-Andre; DeWitt, Jamie C; Thevenet-Morrison, Kelly; Conka, Kamil; Drobna, Beata; Chovancova, Jana; Thurston, Sally W; Lawrence, B Paige; Dozier, Ann M; Jarvinen, Kirsi M; Patayova, Henrieta; Trnovec, Tomas; Legler, Juliette; Hertz-Picciotto, Irva; Lamoree, Marja H. Demographic, Reproductive, and Dietary Determinants of Perfluorooctane Sulfonic (PFOS) and Perfluorooctanoic Acid (PFOA) Concentrations in Human Colostrum. Environmental science & technology. 2016. 50:7152-62 | PFAS | Slovakia |
| Juszkiewicz, T. Residues of xenobiotics in animals, milk, and environment in the light of a 13 year surveillance. Veterinary pharmacology and toxicology. 1983. :641-645 | Pb, Cd | Poland |
| Kadar, Hanane; Veyrand, Bruno; Barbarossa, Andrea; Pagliuca, Giampiero; Legrand, Arnaud; Bosher, Cecile; Boquien, Clair-Yves; Durand, Sophie; Monteau, Fabrice; Antignac, Jean-Philippe; Le Bizec, Bruno. Development of an analytical strategy based on liquid chromatography-high resolution mass spectrometry for measuring perfluorinated compounds in human breast milk: application to the generation of preliminary data regarding perinatal exposure in France. Chemosphere. 2011. 85:473-80 | PFAS | France |
| Kang, Habyeong; Choi, Kyungho; Lee, Haeng-Shin; Kim, Do-Hee; Park, Na-Youn; Kim, Sunmi; Kho, Younglim. Elevated levels of short carbon-chain PFCAs in breast milk among Korean women: Current status and potential challenges. Environmental research. 2016. 148:351-359 | PFAS | Korea (Republic of) |
| Kantol, M; Vartiainen, T. Changes in selenium, zinc, copper and cadmium contents in human milk during the time when selenium has been supplemented to fertilizers in Finland. Journal of trace elements in medicine and biology : organ of the Society for Minerals and Trace Elements (GMS). 2001. 15:7-Nov | Cd | Finland |
| Karrman, Anna; Domingo, Jose L; Llebaria, Xavier; Nadal, Marti; Bigas, Esther; van Bavel, Bert; Lindstrom, Gunilla. Biomonitoring perfluorinated compounds in Catalonia, Spain: concentrations and trends in human liver and milk samples. Environmental science and pollution research international. 2010. 17:750-8 | PFAS | Spain |
| Karrman, Anna; Ericson, Ingrid; van Bavel, Bert; Darnerud, Per Ola; Aune, Marie; Glynn, Anders; Lignell, Sanna; Lindstrom, Gunilla. Exposure of perfluorinated chemicals through lactation: levels of matched human milk and serum and a temporal trend, 1996-2004, in Sweden. Environmental health perspectives. 2007. 115:226-30 | PFAS | Sweden |
| Kelishadi, Roya; Hasanghaliaei, Najmeh; Poursafa, Parinaz; Keikha, Mojtaba; Ghannadi, Alireza; Yazdi, Maryam; Rahimi, Ebrahim. A randomized controlled trial on the effects of jujube fruit on the concentrations of some toxic trace elements in human milk. Journal of research in medical sciences : the official journal of Isfahan University of Medical Sciences. 2016. 21:108 | As, Pb, Cd | Iran (Islamic Republic of) |
| Khammar, S.; Pourkhabbaz, A.; Behrooz, R. D. Maternal determinants of breast-milk, hair and saliva mercury concentrations case study: (Zahedan city). International Research Journal of Applied and Basic Sciences. 2015. 9:440-448 | Hg | Iran (Islamic Republic of) |
| Khanjani, Narges; Jafari, Majideh; Ahmadi Mousavi, Effat. Breast milk contamination with lead and cadmium and its related factors in Kerman, Iran. Journal of environmental health science & engineering. 2018. 16:323-335 | Pb, Cd | Iran (Islamic Republic of) |
| Kilic Altun, Serap; Dinc, Hikmet; Temamogullari, Fusun Karacal; Paksoy, Nilgun. Analyses of Essential Elements and Heavy Metals by Using ICP-MS in Maternal Breast Milk from Sanliurfa, Turkey. International journal of analytical chemistry. 2018. 2018:1784073 | As, Pb | Turkiye |
| Kim J.H.; Moon N.; Lee J.-W.; Mehdi Q.; Yun M.-H.; Moon H.-B. Time-course trend and influencing factors for per- and polyfluoroalkyl substances in the breast milk of Korean mothers. Chemosphere. 2023. 310:136688 | PFAS | Korea (Republic of) |
| Kim, Ju Hee; Moon, Nalae; Ji, Eunsun; Moon, Hyo-Bang. Effects of postnatal exposure to phthalate, bisphenol a, triclosan, parabens, and per- and poly-fluoroalkyl substances on maternal postpartum depression and infant neurodevelopment: a korean mother-infant pair cohort study. Environmental science and pollution research international. 2023. 30:96384-96399 | PFAS | Korea (Republic of) |
| Kim, Seung-Kyu; Lee, Kyu Tae; Kang, Chang Seong; Tao, Lin; Kannan, Kurunthachalam; Kim, Kyung-Ryul; Kim, Chan-Kook; Lee, Jung Suk; Park, Pan Soo; Yoo, Yung Wook; Ha, Jeong Yi; Shin, Yong-Seung; Lee, Jong-Hyeon. Distribution of perfluorochemicals between sera and milk from the same mothers and implications for prenatal and postnatal exposures. Environmental pollution (Barking, Essex : 1987). 2011. 159:169-174 | PFAS | Korea (Republic of) |
| Kim, Sunmi; Choi, Kyungho; Ji, Kyunghee; Seo, Jihyeon; Kho, Younglim; Park, Jeongim; Kim, Sungkyoon; Park, Seokhwan; Hwang, Incheol; Jeon, Jongkwan; Yang, Hyeran; Giesy, John P. Trans-placental transfer of thirteen perfluorinated compounds and relations with fetal thyroid hormones. Environmental science & technology. 2011. 45:7465-72 | PFAS | Korea (Republic of) |
| Kim, Sunmi; Eom, Soyong; Kim, Hai-Joong; Lee, Jeong Jae; Choi, Gyuyeon; Choi, Sooran; Kim, Sungjoo; Kim, Su Young; Cho, Geumjoon; Kim, Young Don; Suh, Eunsook; Kim, Sung Koo; Kim, Seunghyo; Kim, Gun-Ha; Moon, Hyo-Bang; Park, Jeongim; Kim, Sungkyoon; Choi, Kyungho; Eun, So-Hee. Association between maternal exposure to major phthalates, heavy metals, and persistent organic pollutants, and the neurodevelopmental performances of their children at 1 to 2years of age- CHECK cohort study. The Science of the total environment. 2018. 624:377-384 | Pb, Hg | Korea (Republic of) |
| Klein, Laura D; Breakey, Alicia A; Scelza, Brooke; Valeggia, Claudia; Jasienska, Grazyna; Hinde, Katie. Concentrations of trace elements in human milk: Comparisons among women in Argentina, Namibia, Poland, and the United States. PloS one. 2017. 12:e0183367 | As, Pb, Cd | Argentina, Poland, United States |
| Klopov, V P. Levels of heavy metals in women residing in the Russian Arctic. International journal of circumpolar health. 1998. 57 Suppl 1:582-5 | Pb, Hg | Russian Federation |
| Kovar, I Z; Strehlow, C D; Richmond, J; Thompson, M G. Perinatal lead and cadmium burden in a British urban population. Archives of disease in childhood. 1984. 59:36-9 | Pb, Cd | United Kingdom |
| Koyashiki, Gina Ayumi Kobayashi; Paoliello, Monica Maria Bastos; Matsuo, Tiemi; de Oliveira, Marcia Maria Benevenuto; Mezzaroba, Leda; Carvalho, Maria de Fatima; Sakuma, Alice Momoyo; Turini, Conceicao; Vannuchi, Marli Terezinha Oliveira; Barbosa, Claudia Santiago Dias. Lead levels in milk and blood from donors to the Breast Milk Bank in Southern Brazil. Environmental research. 2010. 110:265-71 | Pb | Brazil |
| Krachler M.; Rossipal E. The assessment of concentrations of trace elements in human milk during lactation. Paediatria Croatica. 1997. 41:91-95 | Pb, Cd | Croatia |
| Krachler, M; Li, F S; Rossipal, E; Irgolic, K J. Changes in the concentrations of trace elements in human milk during lactation. Journal of trace elements in medicine and biology : organ of the Society for Minerals and Trace Elements (GMS). 1998. 12:159-76 | Pb, Cd | Austria |
| Krachler, M; Prohaska, T; Koellensperger, G; Rossipal, E; Stingeder, G. Concentrations of selected trace elements in human milk and in infant formulas determined by magnetic sector field inductively coupled plasma-mass spectrometry. Biological trace element research. 2000. 76:97-112 | As | Austria |
| Krachler, M; Rossipal, E; Micetic-Turk, D. Trace element transfer from the mother to the newborn--investigations on triplets of colostrum, maternal and umbilical cord sera. European Journal of Clinical Nutrition. 1999. 53:486-494 | Pb, Cd | Slovenia |
| Kubwabo, Cariton; Kosarac, Ivana; Lalonde, Kaela. Determination of selected perfluorinated compounds and polyfluoroalkyl phosphate surfactants in human milk. Chemosphere. 2013. 91:771-7 | PFAS | Canada |
| Kunter, Imge; Hurer, Nazife; Gulcan, Hayrettin Ozan; Ozturk, Baris; Dogan, Irfan; Sahin, Gonul. Assessment of Aflatoxin M1 and Heavy Metal Levels in Mothers Breast Milk in Famagusta, Cyprus. Biological trace element research. 2017. 175:42-49 | As, Pb, Cd, Hg | Cyprus |
| Lamm, S; Cole, B; Glynn, K; Ullmann, W. Lead content of milks fed to infants--1971-1972. The New England journal of medicine. 1973. 289:574-5 | Pb | United States |
| Lankova, Darina; Lacina, Ondrej; Pulkrabova, Jana; Hajslova, Jana. The determination of perfluoroalkyl substances, brominated flame retardants and their metabolites in human breast milk and infant formula. Talanta. 2013. 117:318-25 | PFAS | Czechia |
| Lara P.C.P.; Silveira J.N.; Neto W.B.; Beinner M.A.; da Silva J.B.B. Validation of a method for direct cadmium determination in breast milk by graphite furnace atomic absorption spectrometry using tantalum treated graphite tube and co-injection of iridium as chemical modifier. Journal of Chemical and Pharmaceutical Research. 2015. 7:366-371 | Cd | Brazil |
| Lara, P. C. P.; Silveira, J. N.; Neto, W. B.; Beinner, M. A.; Silva, J. B. B. da. Use of multivariate optimization to develop methods for direct copper and lead determination in breast milk by graphite furnace atomic absorption spectrometry. Food Analytical Methods. 2014. 7:790-797 | Pb | Brazil |
| Larsson, B; Slorach, S A; Hagman, U; Hofvander, Y. WHO collaborative breast feeding study. II. Levels of lead and cadmium in Swedish human milk, 1978-1979. Acta paediatrica Scandinavica. 1981. 70:281-4 | Pb, Cd | Sweden |
| Lee, Sunggyu; Kim, Sunmi; Park, Jeongim; Kim, Hai-Joong; Choi, Gyuyeon; Choi, Sooran; Kim, Sungjoo; Kim, Su Young; Kim, Sungkyoon; Choi, Kyungho; Moon, Hyo-Bang. Perfluoroalkyl substances (PFASs) in breast milk from Korea: Time-course trends, influencing factors, and infant exposure. The Science of the total environment. 2018. 612:286-292 | PFAS | Korea (Republic of) |
| Lenters V.; Cechova E.; Kocan A.; Legler J.; Leonards P.; Forns J.; Iszatt N.; Stigum H.; Eggesbo M. Early-life exposure to persistent organic pollutants and attention-deficit/hyperactivity disorder: A multi-pollutant assessment of a norwegian birth cohort. Occupational and Environmental Medicine. 2018. 75:A34-A35 | PFAS | Norway |
| Lenters, Virissa; Iszatt, Nina; Forns, Joan; Cechova, Eliska; Kocan, Anton; Legler, Juliette; Leonards, Pim; Stigum, Hein; Eggesbo, Merete. Early-life exposure to persistent organic pollutants (OCPs, PBDEs, PCBs, PFASs) and attention-deficit/hyperactivity disorder: A multi-pollutant analysis of a Norwegian birth cohort. Environment international. 2019. 125:33-42 | PFAS | Norway |
| Leotsinidis, Michalis; Alexopoulos, Athanasios; Kostopoulou-Farri, Evangelia. Toxic and essential trace elements in human milk from Greek lactating women: association with dietary habits and other factors. Chemosphere. 2005. 61:238-47 | Pb, Cd | Greece |
| Levi M.; Hjelm C.; Harari F.; Vahter M. ICP-MS measurement of toxic and essential elements in human breast milk. A comparison of alkali dilution and acid digestion sample preparation methods. Clinical Biochemistry. 2018. 53:81-87 | Cd | Argentina |
| Li S.; Wang G.; Fu K.; Qi C.; Wu X.; Kong Q.; Liang Y. Influencing factors of colostrum exposure to low-level lead and their relationship with early neural development of infants. Neural Regeneration Research. 2007. 2:557-560 | Pb | China |
| Li, Qiyao; Zhang, Yan; Chen, Chen; Lou, Jianlin; Wang, Shenghang; Hang, Jin Guo; Nakayama, Shoji F; Kido, Teruhiko; Feng, Hao; Sun, Xian Liang; Shan, Jiancong. Association Between Prenatal Exposure to Per- and Poly-Fluoroalkyl Substances From Electronic Waste Disassembly Areas and Steroid Hormones in Human Milk Samples. GeoHealth. 2024. 8:e2024GH001142 | PFAS | China |
| Lin YungChieh; Chang WeiHsiang; Li TsunChing; Iwata, O.; Chen HsiuLing. Health risk of infants exposed to lead and mercury through breastfeeding. Exposure and Health. 2022. 15:255-267 | Pb, Hg | China |
| Lin, Xiaoying; Wu, Xiaoling; Li, Xiangyu; Zhang, Duo; Zheng, Quanzhi; Xu, Jiayi; Lu, Shaoyou. Infant exposure to trace elements in breast milk, infant formulas and complementary foods from southern China. The Science of the total environment. 2022. 838:156597 | As, Pb, Cd | China |
| Linares, Ana Maria; Thaxton-Wiggins, Amanda; Unrine, Jason M. Concentrations of Lead and Arsenic in Mother's Milk and Children's Blood in Peruvian Breastfeeding Dyads. Journal of human lactation : official journal of International Lactation Consultant Association. 2024. 40:69-79 | As, Pb | Peru |
| Llorca, Marta; Farre, Marinella; Pico, Yolanda; Teijon, Marisa Lopez; Alvarez, Juan G; Barcelo, Damia. Infant exposure of perfluorinated compounds: levels in breast milk and commercial baby food. Environment international. 2010. 36:584-92 | PFAS | Spain |
| Macheka, Linda R; Abafe, Ovokeroye A; Mugivhisa, Liziwe L; Olowoyo, Joshua O. Occurrence and infant exposure assessment of per and polyfluoroalkyl substances in breast milk from South Africa. Chemosphere. 2022. 288:132601 | PFAS | South Africa |
| Mahfouz, Maya; Harmouche-Karaki, Mireille; Matta, Joseph; Mahfouz, Yara; Salameh, Pascale; Younes, Hassan; Helou, Khalil; Finan, Ramzi; Abi-Tayeh, Georges; Meslimani, Mohamad; Moussa, Ghada; Chahrour, Nada; Osseiran, Camille; Skaiki, Farouk; Narbonne, Jean-Francois. Maternal Serum, Cord and Human Milk Levels of Per- and Polyfluoroalkyl Substances (PFAS), Association with Predictors and Effect on Newborn Anthropometry. Toxics. 2023. 11: | PFAS | Lebanon |
| Mandia, Natalia; Bermejo-Barrera, Pilar; Herbello, Paloma; Lopez-Suarez, Olalla; Fraga, Jose M; Fernandez-Perez, Cristina; Couce, Maria L. Human Milk Concentrations of Minerals, Essential and Toxic Trace Elements and Association with Selective Medical, Social, Demographic and Environmental Factors. Nutrients. 2021. 13: | As, Pb, Cd, Hg | Spain |
| Mansour S.A.A.; Mohmoud A.M.A.; Ibrahim H.I.M.; Abdelghaffar M.A.S.; Marai A.A.M.; Abdul-Aziz A.E.; Abdrabo A.E.M. Mercury And Lead Levels in Pregnant Women And Their Newborns After Delivery in Cairo, Egypt. NeuroQuantology. 2022. 20:884-891 | Pb, Hg | Egypt |
| Mansouri, Borhan; Azadi, Nammam Ali; Sharafi, Kiomars; Nakhaee, Samaneh. The effects of active and passive smoking on selected trace element levels in human milk. Scientific reports. 2023. 13:20756 | As, Pb, Cd, Hg | Iran (Islamic Republic of) |
| Marques, Rejane C; Bernardi, Jose V E; Dorea, Jose G; de Fatima R Moreira, Maria; Malm, Olaf. Perinatal multiple exposure to neurotoxic (lead, methylmercury, ethylmercury, and aluminum) substances and neurodevelopment at six and 24 months of age. Environmental pollution (Barking, Essex : 1987). 2014. 187:130-5 | Pb, Hg | Brazil |
| Marques, Rejane C; Dorea, Jose G; Bastos, Wanderley R; Malm, Olaf. Changes in children hair-Hg concentrations during the first 5 years: maternal, environmental and iatrogenic modifying factors. Regulatory toxicology and pharmacology : RTP. 2007. 49:17-24 | Hg | Brazil |
| Marques, Rejane C; Dorea, Jose G; Bernardi, Jose V E; Bastos, Wanderley R; Malm, Olaf. Prenatal and postnatal mercury exposure, breastfeeding and neurodevelopment during the first 5 years. Cognitive and behavioral neurology : official journal of the Society for Behavioral and Cognitive Neurology. 2009. 22:134-41 | Hg | Brazil |
| Marques, Rejane C; Moreira, Maria de Fatima R; Bernardi, Jose Vicente E; Dorea, Jose G. Breast milk lead concentrations of mothers living near tin smelters. Bulletin of environmental contamination and toxicology. 2013. 91:549-54 | Pb | Brazil |
| Martin, Julia; Rodriguez-Gomez, Rocio; Zafra-Gomez, Alberto; Alonso, Esteban; Vilchez, Jose L; Navalon, Alberto. Polar stir bars for isolation and preconcentration of perfluoroalkyl substances from human milk samples prior to UHPLC-MS/MS analysis. Bioanalysis. 2016. 8:633-47 | PFAS | Spain |
| Martinez, Mari Angeles; Castro, Irma; Rovira, Joaquim; Ares, Susana; Rodriguez, Juan Miguel; Cunha, Sara Cristina; Casal, Susana; Fernandes, Jose Oliveira; Schuhmacher, Marta; Nadal, Marti. Early-life intake of major trace elements, bisphenol A, tetrabromobisphenol A and fatty acids: Comparing human milk and commercial infant formulas. Environmental research. 2019. 169:246-255 | As, Pb, Cd, Hg | Spain |
| Mata, Luis; Sanchez, Lourdes; Puyol, Pilar; Calvo, Miguel. Changes in the Distribution of Cadmium and Lead in Human and Bovine Milk Induced by Heating or Freezing. Journal of food protection. 1996. 59:46-50 | Pb, Cd | Spain |
| Michalke, B; Schramel, P. Protein fractionation and Cd-speciation in human breast milk by HPLC and voltammetry. Journal of trace elements and electrolytes in health and disease. 1990. 4:163-7 | Cd | Germany |
| Miklavcic, A; Cuderman, P; Mazej, D; Snoj Tratnik, J; Krsnik, M; Planinsek, P; Osredkar, J; Horvat, M. Biomarkers of low-level mercury exposure through fish consumption in pregnant and lactating Slovenian women. Environmental research. 2011. 111:1201-7 | Hg | Slovenia |
| Miklavcic, Ana; Casetta, Anica; Snoj Tratnik, Janja; Mazej, Darja; Krsnik, Mladen; Mariuz, Marika; Sofianou, Katia; Spiric, Zdravko; Barbone, Fabio; Horvat, Milena. Mercury, arsenic and selenium exposure levels in relation to fish consumption in the Mediterranean area. Environmental research. 2013. 120:17-Jul | As, Hg | Croatia, Greece, Italy, Slovenia |
| Milnerowicz H.; Chmarek M. Effect of smoking on concentrations of cadmium, copper, iron and zinc in early transitional human milk. Acta Toxicologica. 2003. 11:85-91 | Cd | Poland |
| Milnerowicz, H. Concentration of metals, ceruloplasmin, metallothionein and activity of N-acetyl-beta-D-glucosaminidase and gamma-glutamyltransferase in pregnant women who smoke and in those environmentally exposed to tobacco and in their infants. II. Influence of enviro. International journal of occupational medicine and environmental health. 1997. 10:273-82 | Cd | Poland |
| Milnerowicz, H; Slowinska, M. Concentration of metals, ceruloplasmin, metallothionein and the activity of N-acetyl-beta-D-glucosaminidase and gamma-glutamyltransferase in pregnant women who smoke and in those environmentally exposed to tobacco smoke and in their infants. Part I. International journal of occupational medicine and environmental health. 1997. 10:187-202 | Cd | Poland |
| Milnerowicz, Halina; Chmarek, Monika. Influence of smoking on metallothionein level and other proteins binding essential metals in human milk. Acta paediatrica (Oslo, Norway : 1992). 2005. 94:402-6 | Cd | Poland |
| Mohd-Taufek, N.; Cartwright, D.; Davies, M.; Hewavitharana, A. K.; Koorts, P.; Shaw, P. N.; Sumner, R.; Lee, E.; Whitfield, K. The simultaneous analysis of eight essential trace elements in human milk by ICP-MS. Food Analytical Methods. 2016. 9:2068-2075 | As, Pb, Cd | Australia |
| Mosch, C; Kiranoglu, M; Fromme, H; Volkel, W. Simultaneous quantitation of perfluoroalkyl acids in human serum and breast milk using on-line sample preparation by HPLC column switching coupled to ESI-MS/MS. Journal of chromatography. B, Analytical technologies in the biomedical and life sciences. 2010. 878:2652-8 | PFAS | Germany |
| Motas Guzman, Miguel; Clementini, Chiara; Perez-Carceles, Maria Dolores; Jimenez Rejon, Sandra; Cascone, Aurora; Martellini, Tania; Guerranti, Cristiana; Cincinelli, Alessandra. Perfluorinated carboxylic acids in human breast milk from Spain and estimation of infant's daily intake. The Science of the total environment. 2016. 544:595-600 | PFAS | Spain |
| Motas, Miguel; Jimenez, Sandra; Oliva, Jose; Camara, Miguel Angel; Perez-Carceles, Maria Dolores. Heavy Metals and Trace Elements in Human Breast Milk from Industrial/Mining and Agricultural Zones of Southeastern Spain. International journal of environmental research and public health. 2021. 18: | As, Pb, Cd, Hg | Spain |
| Muller C. Cadmium content of human milk. Trace Elements in Medicine. 1987. 4:7-Apr | Cd | Germany |
| Murthy, G K; Rhea, U S. Cadmium, copper, iron, lead, manganese, and zinc in evaporated milk, infant products, and human milk. Journal of dairy science. 1971. 54:1001-5 | Pb, Cd | United States |
| Nakhaee, Samaneh; Shadmani, Fatemeh Khosravi; Sharafi, Kiomars; Kiani, Amir; Azadi, Nammam Ali; Mansouri, Borhan; Karamimatin, Behzad; Farnia, Vahid. Evaluation of some toxic metals in breast milk samples with dietary and sociodemographic characteristics: a case study of Kermanshah, Western Iran. Environmental science and pollution research international. 2023. 30:4502-4509 | As, Pb, Cd, Hg | Iran (Islamic Republic of) |
| Narres H.-D.; Mohl C.; Stoeppler M. Metal analysis in difficult materials with platform furnace zeeman-atomic absorption spectroscopy. 2. Direct determination of cadmium and lead in milk. . 1985. 181:111-116 | Pb, Cd | Germany |
| Nashashibi, N; Cardamakis, E; Bolbos, G; Tzingounis, V. Investigation of kinetic of lead during pregnancy and lactation. Gynecologic and obstetric investigation. 1999. 48:158-62 | Pb | Greece |
| Naspolini, Nathalia Ferrazzo; Vanzele, Pedro A R; Totolo, Pedro; Schuroff, Paulo Alfonso; Fatori, Daniel; Vicentini Neto, Santos Alves; Barata-Silva, Cristiane; Dos Santos, Lisia Maria Gobbo; Fujita, Andre; Passos-Bueno, Maria Rita; Beltrao-Braga, Patricia C B; Campos, Alline C; Carvalho, Andre C P L F; Polanczyk, Guilherme V; Moreira, Josino Costa; Taddei, Carla R. Lead contamination in human milk affects infants' language trajectory: results from a prospective cohort study. Frontiers in public health. 2024. 12:1450570 | As, Pb, Cd, Hg | Brazil |
| Nazarpour, S.; Teymouri, L.; Teimoori, S.; Nojoumi, S. A.; Moghimi, A. Investigation of Cd(II) concentrations in mother's milk of Varamin region of Iran. Asian Journal of Chemistry. 2013. 25:5402-5406 | Cd | Iran (Islamic Republic of) |
| Nazlican, Ersin; Arica, Enes; Goren, Ismail Ethem; Kilincli, Betul; Mete, Burak; Daglioglu, Nebile. The risk estimation and assessment of heavy metal exposure by biomonitoring in the breast milk of mothers in the Cukurova Region, Turkey. Environmental science and pollution research international. 2022. 29:13963-13970 | As, Pb, Cd, Hg | Turkiye |
| Needham, Larry L; Grandjean, Philippe; Heinzow, Birger; Jorgensen, Poul J; Nielsen, Flemming; Patterson, Donald G Jr; Sjodin, Andreas; Turner, Wayman E; Weihe, Pal. Partition of environmental chemicals between maternal and fetal blood and tissues. Environmental science & technology. 2011. 45:1121-6 | Pb, Cd, Hg, PFAS | Denmark |
| Nicolle-Mir L. Transfer of blood lead to breast milk: Kinetics and contribution to infant exposure. Environnement, Risques et Sante. 2014. 13:450-451 | Pb | France |
| Nishijo, M; Nakagawa, H; Honda, R; Tanebe, K; Saito, S; Teranishi, H; Tawara, K. Effects of maternal exposure to cadmium on pregnancy outcome and breast milk. Occupational and environmental medicine. 2002. 59:394-397 | Cd | Japan |
| Norouzi, Elaheh; Bahramifar, Nader; Ghasempouri, Seyed Mahmoud. Effect of teeth amalgam on mercury levels in the colostrums human milk in Lenjan. Environmental monitoring and assessment. 2012. 184:375-80 | Hg | Iran (Islamic Republic of) |
| Nyberg, Elisabeth; Awad, Raed; Bignert, Anders; Ek, Caroline; Sallsten, Gerd; Benskin, Jonathan P. Inter-individual, inter-city, and temporal trends of per- and polyfluoroalkyl substances in human milk from Swedish mothers between 1972 and 2016. Environmental science. Processes & impacts. 2018. 20:1136-1147 | PFAS | Sweden |
| Okati, N.; Sari, A. E.; Ghasempouri, S. M. Evaluation of mercury pollution in breast milk and Iranian infants' hair. International Research Journal of Applied and Basic Sciences. 2013. 4:2857-2864 | Hg | Iran (Islamic Republic of) |
| Olowoyo, Joshua O; Macheka, Linda R; Mametja, Phiona M. Health Risk Assessments of Selected Trace Elements and Factors Associated with Their Levels in Human Breast Milk from Pretoria, South Africa. International journal of environmental research and public health. 2021. 18: | As, Pb, Cd | South Africa |
| Olszowski, Tomasz; Baranowska-Bosiacka, Irena; Rebacz-Maron, Ewa; Gutowska, Izabela; Jamiol, Dominika; Prokopowicz, Adam; Goschorska, Marta; Chlubek, Dariusz. Cadmium Concentration in Mother's Blood, Milk, and Newborn's Blood and Its Correlation with Fatty Acids, Anthropometric Characteristics, and Mother's Smoking Status. Biological trace element research. 2016. 174:20-Aug | Cd | Poland |
| Onieva Aguayo C.; Coll Risco I.; Romero Gallardo L.; Ocon O.; Gascon Cortes E.; Moreno L.; Aranda P.; Llopis J.; Aparicio V.A.; Sanchez Gonzalez C. Association between postpartum maternal body composition and colostrum breast milk mineral composition. The GESTAFIT project. Ars Pharmaceutica. 2018. 59:94 | As | Spain |
| Orun, Emel; Yalcin, S Songul; Aykut, Osman. Lead, mercury, and cadmium levels in breast milk and infant hair in the late period of lactation in Ankara, Turkey. International journal of environmental health research. 2022. 32:1950-1961 | Pb, Cd, Hg | Turkiye |
| Orun, Emel; Yalcin, S Songul; Aykut, Osman; Orhan, Gunnur; Koc-Morgil, Goksel; Yurdakok, Kadriye; Uzun, Ramazan. Mercury exposure via breast-milk in infants from a suburban area of Ankara, Turkey. The Turkish journal of pediatrics. 2012. 54:136-43 | Hg | Turkiye |
| Orun, Emel; Yalcin, S Songul; Aykut, Osman; Orhan, Gunnur; Morgil, Goksel Koc; Yurdakok, Kadriye; Uzun, Ramazan. Breast milk lead and cadmium levels from suburban areas of Ankara. The Science of the total environment. 2011. 409:2467-72 | Pb, Cd | Turkiye |
| Oskarsson, A; Palminger Hallen, I; Sundberg, J. Exposure to toxic elements via breast milk. The Analyst. 1995. 120:765-70 | Pb, Hg | Sweden |
| Oskarsson, A; Schultz, A; Skerfving, S; Hallen, I P; Ohlin, B; Lagerkvist, B J. Total and inorganic mercury in breast milk in relation to fish consumption and amalgam in lactating women. Archives of environmental health. 1996. 51:234-41 | Hg | Sweden |
| Pajewska-Szmyt, Martyna; Buszewski, Boguslaw; Gadzala-Kopciuch, Renata. Carbon dots as rapid assays for detection of mercury(II) ions based on turn-off mode and breast milk. Spectrochimica acta. Part A, Molecular and biomolecular spectroscopy. 2020. 236:118320 | Hg | Poland |
| Pangestuti, D. R.; Kartini, A.; Suhartono, S.; Lisnawati, N.; Putranto, T. T.; Susanto, N. Identification of lead contamination in the food chain and environment surrounding breastfeeding mothers in highland agricultural areas. Jurnal Kesehatan Lingkungan / Journal of Environmental Health. 2025. 17:22-30 | Pb | Indonesia |
| Parizek, Ondrej; Gramblicka, Tomas; Parizkova, Denisa; Polachova, Andrea; Bechynska, Kamila; Dvorakova, Darina; Stupak, Michal; Dusek, Jiri; Pavlikova, Jitka; Topinka, Jan; Sram, Radim J; Pulkrabova, Jana. Assessment of organohalogenated pollutants in breast milk from the Czech Republic. The Science of the total environment. 2023. 871:161938 | PFAS | Czechia |
| Park, Yelim; Lee, Aram; Choi, Kyungho; Kim, Hai-Joong; Lee, Jeong Jae; Choi, Gyuyeon; Kim, Sungjoo; Kim, Su Young; Cho, Geum Joon; Suh, Eunsook; Kim, Sung Koo; Eun, So-Hee; Eom, Soyong; Kim, Seunghyo; Kim, Gun-Ha; Moon, Hyo-Bang; Kim, Sungkyoon; Choi, Sooran; Kim, Young Don; Kim, Jinho; Park, Jeongim. Exposure to lead and mercury through breastfeeding during the first month of life: A CHECK cohort study. The Science of the total environment. 2018. 612:876-883 | Pb, Hg | Korea (Republic of) |
| Parr, R M; DeMaeyer, E M; Iyengar, V G; Byrne, A R; Kirkbright, G F; Schoch, G; Niinisto, L; Pineda, O; Vis, H L; Hofvander, Y. Minor and trace elements in human milk from Guatemala, Hungary, Nigeria, Philippines, Sweden, and Zaire. Results from a WHO/IAEA joint project. Biological trace element research. 1991. 29:51-75 | As, Pb, Cd, Hg | Hungary, Philippines, Sweden |
| Perrone, L; Di Palma, L; Di Toro, R; Gialanella, G; Moro, R. Interaction of trace elements in a longitudinal study of human milk from full-term and preterm mothers. Biological trace element research. 1994. 41:321-30 | Pb | Italy |
| Perrone, L; Di Palma, L; Di Toro, R; Gialanella, G; Moro, R. Trace element content of human milk during lactation. Journal of trace elements and electrolytes in health and disease. 1993. 7:245-7 | Pb | Italy |
| Petrovic, S. M.; Savic, S. R.; Petronijevic, Z. B. Macro- and micro-element analysis in milk samples by inductively coupled plasma - optical emission spectrometry. Acta Periodica Technologica. 2016. 47:51-62 | Pb, Cd | Serbia |
| Pico, Yolanda; Farre, Marinella; Barcelo, Damia. Quantitative profiling of perfluoroalkyl substances by ultrahigh-performance liquid chromatography and hybrid quadrupole time-of-flight mass spectrometry. Analytical and bioanalytical chemistry. 2015. 407:4247-59 | PFAS | Spain |
| Pietrzak-Fiecko, R.; Smoczynski, S. S. Cadmium and lead contents in human milk, cow's milk and infant formulas. Polish Journal of Natural Sciences. 2006. 21:1105-1112 | Pb, Cd | Poland |
| Pitkin, R M; Bahns, J A; Filer, L J Jr; Reynolds, W A. Mercury in human maternal and cord blood, placenta, and milk. Proceedings of the Society for Experimental Biology and Medicine. Society for Experimental Biology and Medicine (New York, N.Y.). 1976. 151:565-7 | Hg | United States |
| Plockinger B.; Ulm M.R.; Golaszewski T.; Meisinger V.; Suzin J.; Grudzinska M.; Zdziennicki A.; Dadak C. Lead, mercury, and cadmium exposure of neonates in Poland compared to Austria and other European countries. Trace Elements and Electrocytes. 1996. 13:22-25 | Pb, Cd, Hg | Poland |
| Poniedzialek, Barbara; Rzymski, Piotr; Piet, Malgorzata; Gasecka, Monika; Stroinska, Anna; Niedzielski, Przemyslaw; Mleczek, Miroslaw; Rzymski, Pawel; Wilczak, Maciej. Relation between polyphenols, malondialdehyde, antioxidant capacity, lactate dehydrogenase and toxic elements in human colostrum milk. Chemosphere. 2018. 191:548-554 | As, Pb, Cd | Poland |
| Pratt, Iona; Anderson, Wayne; Crowley, Dominique; Daly, Sean; Evans, Rhodri; Fernandes, Alwyn; Fitzgerald, Margaret; Geary, Michael; Keane, Declan; Morrison, John J; Reilly, Alan; Tlustos, Christina. Brominated and fluorinated organic pollutants in the breast milk of first-time Irish mothers: is there a relationship to levels in food?. Food additives & contaminants. Part A, Chemistry, analysis, control, exposure & risk assessment. 2013. 30:1788-98 | PFAS | Ireland |
| Prioteasa, L.; Prodana, M.; Demetrescu, I.; Ionita, D.; Maier, T.; Moisa, M. Heavy metals and other trace elements in the blood and breast milk from two different Romanian areas. Environmental Engineering and Management Journal. 2018. 17:2915-2921 | As, Pb, Cd | Romania |
| Raab, Ulla; Albrecht, Michael; Preiss, Ursula; Volkel, Wolfgang; Schwegler, Ursula; Fromme, Hermann. Organochlorine compounds, nitro musks and perfluorinated substances in breast milk - results from Bavarian Monitoring of Breast Milk 2007/8. Chemosphere. 2013. 93:461-7 | PFAS | Germany |
| Rabinowitz, M; Leviton, A; Needleman, H. Lead in milk and infant blood: a dose-response model. Archives of environmental health. 1985. 40:283-6 | Pb | United States |
| Radisch, B; Luck, W; Nau, H. Cadmium concentrations in milk and blood of smoking mothers. Toxicology letters. 1987. 36:147-52 | Cd | Germany |
| Rahati, Shiva; Hashemi, Mohammad; Orooji, Arezoo; Afshari, Asma; Sany, Seyedeh Belin Tavakoly. Health risk assessments of heavy metals and trace elements exposure in the breast milk of lactating mothers in the Northeastern Iran. Environmental science and pollution research international. 2024. 31:25892-25906 | As, Pb, Cd, Hg | Iran (Islamic Republic of) |
| Rahimi, E.; Hashemi, M.; Baghbadorani, Z. T. Determination of cadmium and lead in human milk. International Journal of Environmental Science and Technology. 2009. 6:671-676 | Pb, Cd | Iran (Islamic Republic of) |
| Raie R.M.; Smith H. Trace element deficiency and cot deaths. Medicine, Science and the Law. 1981. 21:41-46 | As, Hg | United Kingdom |
| Ramirez, G B; Cruz, M C; Pagulayan, O; Ostrea, E; Dalisay, C. The Tagum study I: analysis and clinical correlates of mercury in maternal and cord blood, breast milk, meconium, and infants' hair. Pediatrics. 2000. 106:774-81 | Hg | Philippines |
| Rawn, Dorothea F K; Dufresne, Guy; Clement, Genevieve; Fraser, William D; Arbuckle, Tye E. Perfluorinated alkyl substances in Canadian human milk as part of the Maternal-Infant Research on Environmental Chemicals (MIREC) study. The Science of the total environment. 2022. 831:154888 | PFAS | Canada |
| Rebelo, Fernanda M; Cunha, Leandro R da; Andrade, Patricia D; Costa Junior, Walkimar A da; Bastos, Wanderley R; Caldas, Eloisa D. Mercury in breast milk from women in the Federal District, Brazil and dietary risk assessment for breastfed infants. Journal of trace elements in medicine and biology : organ of the Society for Minerals and Trace Elements (GMS). 2017. 44:99-103 | Hg | Brazil |
| Rica C.C.; Kirkbright G.F. Determination of trace concentrations of lead and nickel in human milk by electrothermal atomisation atomic absorption spectrophotometric and inductively-coupled plasma emission spectroscopy. Science of the Total Environment. 1982. 22:193-201 | Pb | United Kingdom |
| Richmond, J; Strehlow, C D; Chalkley, S R. Dietary intake of Al, Ca, Cu, Fe, Pb, and Zn in infants. British journal of biomedical science. 1993. 50:178-86 | Pb | United Kingdom |
| Rivero Martino, F. A.; Fernandez Sanchez, M. L.; Sanz-Medel, A. The potential of double focusing-ICP-MS for studying elemental distribution patterns in whole milk, skimmed milk and milk whey of different milks. Analytica Chimica Acta. 2001. 442:191-200 | Pb, Cd, Hg | Spain |
| Rockway, S W; Weber, C W; Lei, K Y; Kemberling, S R. Lead concentrations of milk, blood, and hair in lactating women. International archives of occupational and environmental health. 1984. 53:181-7 | Pb | United States |
| Rodriguez Rodriguez, E. M.; Delgado Uretra, E.; Diaz Romero, C. Concentrations of cadmium and lead in different types of milk. Zeitschrift fur Lebensmittel-Untersuchung und -Forschung. A, Food Research and Technology. 1999. 208:162-168 | Pb, Cd | Spain |
| Roosens, Laurence; D'Hollander, Wendy; Bervoets, Lieven; Reynders, Hans; Van Campenhout, Karen; Cornelis, Christa; Van Den Heuvel, Rosette; Koppen, Gudrun; Covaci, Adrian. Brominated flame retardants and perfluorinated chemicals, two groups of persistent contaminants in Belgian human blood and milk. Environmental pollution (Barking, Essex : 1987). 2010. 158:2546-52 | PFAS | Belgium |
| Rossipal E.; Krachler M. Pattern of trace elements in human milk during the course of lactation. Nutrition Research. 1998. 18:24-Nov | Pb, Cd, Hg | Austria |
| Rossipal E.; Krachler M.; Li F.; Micetic-Turk D. Investigation of the transport of trace elements across barriers in humans: Studies of placental and mammary transfer. Acta Paediatrica, International Journal of Paediatrics. 2000. 89:1190-1195 | Pb, Cd | Slovenia |
| Rovira, Joaquim; Martinez, Maria Angeles; Mari, Montse; Cunha, Sara Cristina; Fernandes, Jose Oliveira; Marmelo, Isa; Marques, Antonio; Haug, Line Smastuen; Thomsen, Cathrine; Nadal, Marti; Domingo, Jose L; Schuhmacher, Marta. Mixture of environmental pollutants in breast milk from a Spanish cohort of nursing mothers. Environment international. 2022. 166:107375 | As, Pb, Cd, Hg, PFAS | Spain |
| Sakamoto, Mineshi; Chan, Hing Man; Domingo, Jose L; Kubota, Machi; Murata, Katsuyuki. Changes in body burden of mercury, lead, arsenic, cadmium and selenium in infants during early lactation in comparison with placental transfer. Ecotoxicology and environmental safety. 2012. 84:179-84 | As, Pb, Cd, Hg | Japan |
| Sakamoto, Mineshi; Kubota, Machi; Matsumoto, Shin-ichiro; Nakano, Atsuhiro; Akagi, Hirokatsu. Declining risk of methylmercury exposure to infants during lactation. Environmental research. 2002. 90:185-9 | Hg | Japan |
| Salmani, Mohammad Hossien; Rezaie, Zinab; Mozaffari-Khosravi, Hassan; Ehrampoush, Mohammad Hassan. Arsenic exposure to breast-fed infants: contaminated breastfeeding in the first month of birth. Environmental science and pollution research international. 2018. 25:6680-6684 | As | Iran (Islamic Republic of) |
| Samiee, F.; Leili, M.; Faradmal, J.; Torkshavand, Z.; Asadi, G. Exposure to arsenic through breast milk from mothers exposed to high levels of arsenic in drinking water: infant risk assessment. Food Control. 2019. 106:106669 | As | Iran (Islamic Republic of) |
| Samiee, Fateme; Vahidinia, Aliasghar; Taravati Javad, Masoumeh; Leili, Mostafa. Exposure to heavy metals released to the environment through breastfeeding: A probabilistic risk estimation. The Science of the total environment. 2019. 650:3075-3083 | As, Pb | Iran (Islamic Republic of) |
| Schramel, P; Hasse, S; Ovcar-Pavlu, J. Selenium, cadmium, lead, and mercury concentrations in human breast milk, in placenta, maternal blood, and the blood of the newborn. Biological trace element research. 1988. 15:111-24 | Pb, Cd, Hg | Germany |
| Schramel, P; Lill, G; Hasse, S; Klose, B J. Mineral- and trace element concentrations in human breast milk, placenta, maternal blood, and the blood of the newborn. Biological trace element research. 1988. 16:67-75 | Pb, Cd, Hg | Germany |
| Schulte-Lobbert, F J; Bohn, G. Determination of cadmium in human milk during lactation. Archives of toxicology. 1977. 37:155-7 | Cd | Germany |
| Serrano, Laura; Iribarne-Duran, Luz M; Suarez, Beatriz; Artacho-Cordon, Francisco; Vela-Soria, Fernando; Pena-Caballero, Manuela; Hurtado, Jose A; Olea, Nicolas; Fernandez, Mariana F; Freire, Carmen. Concentrations of perfluoroalkyl substances in donor breast milk in Southern Spain and their potential determinants. International journal of hygiene and environmental health. 2021. 236:113796 | PFAS | Spain |
| Sharafi, Kiomars; Nakhaee, Samaneh; Azadi, Nammam Ali; Mansouri, Borhan; Miri Kermanshahi, Saba; Paknahad, Maedeh; Habibi, Yazdan. Human health risk assessment of potentially toxic elements in the breast milk consumed by infants in Western Iran. Scientific reports. 2023. 13:6656 | As, Pb, Cd, Hg | Iran (Islamic Republic of) |
| Shawahna, Ramzi. Breast milk to blood lead ratios among women from the West Bank of Palestine: a cross-sectional study of associated factors. International breastfeeding journal. 2021. 16:61 | Pb | Palestine, State of |
| Shawahna, Ramzi; Saleh, Rana; Owiwi, Lina; Abdi, Aya; Bani-Odeh, Diana; Maqboul, Iyad; Hijaz, Hatim; Jaber, Mohammad. Breastmilk cadmium levels and estimated infant exposure: a multicenter study of associated factors in a resource-limited country. International breastfeeding journal. 2023. 18:36 | Cd | Palestine, State of |
| Sherlock, J C; Quinn, M J. Relationship between blood lead concentrations and dietary lead intake in infants: the Glasgow Duplicate Diet Study 1979-1980. Food additives and contaminants. 1986. 3:167-76 | Pb | United Kingdom |
| Shi, Li Li; Hang, Jin Guo; Lou, Jianlin; Dong, Jing Jian; Feng, Hao; Wang, Zheng; Shen, Bin; Nakayama, Shoji F; Kido, Teruhiko; Ma, Chaochen; Sun, Xian Liang; Jung, Chau-Ren. Multiple exposures to heavy metals and changes in steroid hormones production in 4-year-old children. Journal of exposure science & environmental epidemiology. 2023. 33:865-873 | Pb, Cd, Hg | China |
| Sikorski, R; Juszkiewicz, T; Paszkowski, T; Radomanski, T; Szkoda, J; Milart, P. Hair trace metal concentration of pregnant women at term in comparison with blood and milk levels. European journal of obstetrics, gynecology, and reproductive biology. 1986. 23:349-57 | Pb | Poland |
| Sikorski, R; Paszkowski, T; Radomanski, T Jr; Szkoda, J. Cadmium contamination of early human milk. Gynecologic and obstetric investigation. 1989. 27:91-3 | Cd | Poland |
| Skerfving, S. Mercury in women exposed to methylmercury through fish consumption, and in their newborn babies and breast milk. Bulletin of environmental contamination and toxicology. 1988. 41:475-82 | Hg | Sweden |
| Snoj Tratnik, Janja; Falnoga, Ingrid; Mazej, Darja; Kocman, David; Fajon, Vesna; Jagodic, Marta; Stajnko, Anja; Trdin, Ajda; Slejkovec, Zdenka; Jeran, Zvonka; Osredkar, Josko; Sesek-Briski, Alenka; Krsnik, Mladen; Kobal, Alfred B; Kononenko, Lijana; Horvat, Milena. Results of the first national human biomonitoring in Slovenia: Trace elements in men and lactating women, predictors of exposure and reference values. International journal of hygiene and environmental health. 2019. 222:563-582 | As, Pb, Cd, Hg | Slovenia |
| Sobhanikia, Mostafa; Nikniaz, Leila; Shakerkhatibi, Mohammad; Vaezi Hir, Abdorreza; Gilani, Neda; Mosaferi, Mohammad. Evaluating Heavy Metals in Human Breast Milk: a Cross-Sectional Study from Mining and Agricultural Areas in Northwestern Iran. Biological trace element research. 2024. : | As | Iran (Islamic Republic of) |
| Soboleva S.E.; Zaksas N.P.; Nevinsky G.A. Comparison of Trace Elements in High-Molecular-Mass Multiprotein Complex and in Female Milk from Which It Was Obtained. Scientific World Journal. 2019. 2019:2578975 | Pb, Cd | Russian Federation |
| Soleimani, Sedigheh; Shahverdy, Mohammad Reza; Mazhari, Najmeh; Abdi, Khosrou; Gerayesh Nejad, Siavash; Shams, Sedigheh; Alebooyeh, Elham; Khaghani, Shahnaz. Lead concentration in breast milk of lactating women who were living in Tehran, Iran. Acta medica Iranica. 2014. 52:56-9 | Pb | Iran (Islamic Republic of) |
| Song, Yoojun; Lee, Chae-Kwan; Kim, Kun-Hyung; Lee, Jong-Tae; Suh, Chunhui; Kim, Se-Yeong; Kim, Jeong-Ho; Son, Byung-Chul; Kim, Dae-Hwan; Lee, Sangyoon. Factors associated with total mercury concentrations in maternal blood, cord blood, and breast milk among pregnant women in Busan, Korea. Asia Pacific journal of clinical nutrition. 2016. 25:340-9 | Hg | Korea (Republic of) |
| Sowers, MaryFran R; Scholl, Theresa O; Hall, Gene; Jannausch, Mary L; Kemp, Francis W; Li, Xinhua; Bogden, John D. Lead in breast milk and maternal bone turnover. American journal of obstetrics and gynecology. 2002. 187:770-6 | Pb | United States |
| Spevackova, V; Rychlik, S; Cejchanova, M; Spevacek, V. Monitoring of trace elements in breast milk sampling and measurement procedures. Central European journal of public health. 2005. 13:85-8 | Pb, Cd | Czechia |
| Stawarz, Robert; Formicki, Grzegorz; Massanyi, Peter. Daily fluctuations and distribution of xenobiotics, nutritional and biogenic elements in human milk in Southern Poland. Journal of environmental science and health. Part A, Toxic/hazardous substances & environmental engineering. 2007. 42:1169-75 | Pb, Cd | Poland |
| Sternowsky, H J; Wessolowski, R. Lead and cadmium in breast milk. Higher levels in urban vs rural mothers during the first 3 months of lactation. Archives of toxicology. 1985. 57:41-5 | Pb, Cd | Germany |
| Sternowsky, Hans-J; Moser, Barbara; Szadkowsky, Dieter. Arsenic in breast milk during the first 3 months of lactation. International journal of hygiene and environmental health. 2002. 205:405-9 | As | Germany |
| Stiboller, Michael; Raber, Georg; Gjengedal, Elin Lovise Folven; Eggesbo, Merete; Francesconi, Kevin A. Quantifying Inorganic Arsenic and Other Water-Soluble Arsenic Species in Human Milk by HPLC/ICPMS. Analytical chemistry. 2017. 89:6265-6271 | As | Austria, Norway |
| Sundstrom, Maria; Ehresman, David J; Bignert, Anders; Butenhoff, John L; Olsen, Geary W; Chang, Shu-Ching; Bergman, Ake. A temporal trend study (1972-2008) of perfluorooctanesulfonate, perfluorohexanesulfonate, and perfluorooctanoate in pooled human milk samples from Stockholm, Sweden. Environment international. 2011. 37:178-83 | PFAS | Sweden |
| Surenjav, Enkhtuul; Lkhasuren, Jargalsaikhan; Fiedler, Heidelore. POPs monitoring in Mongolia - Core matrices. Chemosphere. 2022. 297:134180 | PFAS | Mongolia |
| Szukalska, Marta; Merritt, Thurman Allen; Lorenc, Wiktor; Sroczynska, Katarzyna; Miechowicz, Izabela; Komorowicz, Izabela; Mazela, Jan; Baralkiewicz, Danuta; Florek, Ewa. Toxic metals in human milk in relation to tobacco smoke exposure. Environmental research. 2021. 197:111090 | As, Pb, Cd | Poland |
| Tahboub, Yahya R; Massadeh, Adnan M; Al-Sheyab, Nihaya A; El Shrafat, Diab; Nsserat, Israa A. Levels of Trace Elements in Human Breast Milk in Jordan: a Comparison with Infant Formula Milk Powder. Biological trace element research. 2021. 199:4066-4073 | As, Pb, Cd | Jordan |
| Tao, Lin; Kannan, Kurunthachalam; Wong, Chung M; Arcaro, Kathleen F; Butenhoff, John L. Perfluorinated compounds in human milk from Massachusetts, U.S.A. Environmental science & technology. 2008. 42:3096-101 | PFAS | China, United States |
| Thomsen, Cathrine; Haug, Line S; Stigum, Hein; Froshaug, May; Broadwell, Sharon L; Becher, Georg. Changes in concentrations of perfluorinated compounds, polybrominated diphenyl ethers, and polychlorinated biphenyls in Norwegian breast-milk during twelve months of lactation. Environmental science & technology. 2010. 44:9550-6 | PFAS | Norway |
| Tiran B.; Rossipal E.; Tiran A.; Karpf E.; Lorenz O. Burden of cadmium and lead content in human milk and milk formulas in Styria, Austria. A moderate industrialized area. Trace Elements in Medicine. 1994. 11:42-45 | Pb, Cd | Austria |
| Trdin, A.; Snoj Tratnik, J.; Stajnko, A.; Marc, J.; Mazej, D.; Sesek Briski, A.; Kastelec, D.; Prpic, I.; Petrovic, O.; Spiric, Z.; Horvat, M.; Falnoga, I. Trace elements and APOE polymorphisms in pregnant women and their new-borns. Environment International. 2020. 143: | As, Pb, Cd, Hg | Croatia |
| Turconi, Giovanna; Guarcello, Marianna; Livieri, Chiara; Comizzoli, Sergio; Maccarini, Laura; Castellazzi, Anna Maria; Pietri, Amedeo; Piva, Gianfranco; Roggi, Carla. Evaluation of xenobiotics in human milk and ingestion by the newborn--an epidemiological survey in Lombardy (Northern Italy). European journal of nutrition. 2004. 43:191-7 | Pb, Cd | Italy |
| Ursinyova, M.; Hladikova, V. The intake of selected toxic elements from milk in infants. Fresenius Environmental Bulletin. 1997. 6:627-632 | Pb, Cd, Hg | Slovakia |
| Ursinyova, Monika; Masanova, Vlasta. Cadmium, lead and mercury in human milk from Slovakia. Food additives and contaminants. 2005. 22:579-89 | Pb, Cd, Hg | Slovakia |
| Ursinyova, Monika; Masanova, Vlasta; Uhnakova, Iveta; Murinova, Lubica Palkovicova; Patayova, Henrieta; Rausova, Katarina; Trnovec, Tomas; Stencl, Jan; Gajdos, Martin. Prenatal and Early Postnatal Exposure to Total Mercury and Methylmercury from Low Maternal Fish Consumption. Biological trace element research. 2019. 191:16-26 | Hg | Slovakia |
| Vacchina, Veronique; Seby, Fabienne; Chekri, Rachida; Verdeil, Justine; Dumont, Jean; Hulin, Marion; Sirot, Veronique; Volatier, Jean-Luc; Serreau, Raphael; Rousseau, Alexandra; Simon, Tabassome; Guerin, Thierry. Optimization and validation of the methods for the total mercury and methylmercury determination in breast milk. Talanta. 2017. 167:404-410 | Hg | France |
| Vahidinia, Aliasghar; Samiee, Fateme; Faradmal, Javad; Rahmani, Alireza; Taravati Javad, Masoumeh; Leili, Mostafa. Mercury, Lead, Cadmium, and Barium Levels in Human Breast Milk and Factors Affecting Their Concentrations in Hamadan, Iran. Biological trace element research. 2019. 187:32-40 | Pb, Cd, Hg | Iran (Islamic Republic of) |
| Valent, Francesca; Mariuz, Marika; Bin, Maura; Little, D'Anna; Mazej, Darja; Tognin, Veronica; Tratnik, Janja; McAfee, Alison J; Mulhern, Maria S; Parpinel, Maria; Carrozzi, Marco; Horvat, Milena; Tamburlini, Giorgio; Barbone, Fabio. Associations of prenatal mercury exposure from maternal fish consumption and polyunsaturated fatty acids with child neurodevelopment: a prospective cohort study in Italy. Journal of epidemiology. 2013. 23:360-70 | Hg | Italy |
| van Beijsterveldt, Inge A L P; van Zelst, Bertrand D; de Fluiter, Kirsten S; van den Berg, Sjoerd A A; van der Steen, Manouk; Hokken-Koelega, Anita C S. Poly- and perfluoroalkyl substances (PFAS) exposure through infant feeding in early life. Environment international. 2022. 164:107274 | PFAS | Netherlands |
| Vavilis, D; Bontis, J; Agorastos, T; Angelikakis, G; Zournatzi, V; Loufopoulos, A; Constantinou, A; Patsourou, A. Lead concentrations in early human milk of urban and rural mothers. Clinical and experimental obstetrics & gynecology. 1997. 24:198-9 | Pb | Greece |
| Vela-Soria, Fernando; Serrano-Lopez, Laura; Garcia-Villanova, Javier; de Haro, Tomas; Olea, Nicolas; Freire, Carmen. HPLC-MS/MS method for the determination of perfluoroalkyl substances in breast milk by combining salt-assisted and dispersive liquid-liquid microextraction. Analytical and bioanalytical chemistry. 2020. 412:7913-7923 | PFAS | Spain |
| Ventura, E. F.; Bernabeu, M.; Callejon-Leblic, B.; Cabrera-Rubio, R.; Yeruva, L.; Estan-Capell, J.; Martinez-Costa, C.; Garcia-Barrera, T.; Collado, M. C. Human milk metals and metalloids shape infant microbiota. Food and Function. 2024. 15:12134-12145 | As, Pb, Cd | Spain |
| Vieira, Solange M; de Almeida, Ronaldo; Holanda, Igor B B; Mussy, Marilia H; Galvao, Roberta C F; Crispim, Pedro T B; Dorea, Jose G; Bastos, Wanderley R. Total and methyl-mercury in hair and milk of mothers living in the city of Porto Velho and in villages along the Rio Madeira, Amazon, Brazil. International journal of hygiene and environmental health. 2013. 216:682-9 | Hg | Brazil |
| Vimy, M J; Hooper, D E; King, W W; Lorscheider, F L. Mercury from maternal "silver" tooth fillings in sheep and human breast milk. A source of neonatal exposure. Biological trace element research. 1997. 56:143-52 | Hg | Canada |
| Volkel, W; Genzel-Boroviczeny, O; Demmelmair, H; Gebauer, C; Koletzko, B; Twardella, D; Raab, U; Fromme, H. Perfluorooctane sulphonate (PFOS) and perfluorooctanoic acid (PFOA) in human breast milk: results of a pilot study. International journal of hygiene and environmental health. 2008. 211:440-6 | PFAS | Germany, Hungary |
| Vollset, Marie; Iszatt, Nina; Enger, Oyvind; Gjengedal, Elin Lovise Folven; Eggesbo, Merete. Concentration of mercury, cadmium, and lead in breast milk from Norwegian mothers: Association with dietary habits, amalgam and other factors. The Science of the total environment. 2019. 677:466-473 | Pb, Cd, Hg | Norway |
| von Ehrenstein, Ondine S; Fenton, Suzanne E; Kato, Kayoko; Kuklenyik, Zsuzsanna; Calafat, Antonia M; Hines, Erin P. Polyfluoroalkyl chemicals in the serum and milk of breastfeeding women. Reproductive toxicology (Elmsford, N.Y.). 2009. 27:239-245 | PFAS | United States |
| Vuori, E; Vetter, M; Kuitunen, P; Salmela, S. Cadmium in Finnish breast milk, a longitudinal study. Archives of toxicology. 1983. 53:207-11 | Cd | Finland |
| Walker Jr. B. Lead content of milk and infant formula. Journal of Food Protection. 1980. 43:178-179 | Pb | United States |
| Wang YuChen; Lin YuChen [Lin, Y. C. A. ]; Wang ShuLi; Ding WangHsien. Determination of perfluorochemicals in human milk using isotope-dilution liquid chromatography tandem mass spectrometry. Journal of the Chinese Chemical Society. 2012. 59:544-549 | PFAS | China |
| Wang, Rui; ; Yu, Jiangkun; Chen, Dawei; Li, Jingguang; Qi, Kemin; Ma, Lan; Lyu, Bing; Shang, Xiaohong; Zhao, Yunfeng. Occurrences of heavy metal in breast milk in China: Results of the third national breast milk survey. Journal of hazardous materials. 2025. 486:137104 | As, Pb, Cd, Hg | China |
| Wang, Zheng; ; Liang, Caixia; Shi, Li Li; Zhu, Cheng-Sheng; Wang, Shenghang; Nakayama, Shoji F; Kido, Teruhiko; Sun, Xian Liang; Shan, Jiancong. Associations Between Heavy Metal Exposure from Milk and Steroid Hormones in Mothers. Biological trace element research. 2024. : | As, Pb, Cd | China |
| Winfield, S A; Boyd, N D; Vimy, M J; Lorscheider, F L. Measurement of total mercury in biological specimens by cold vapor atomic fluorescence spectrometry. Clinical chemistry. 1994. 40:206-10 | Hg | Canada |
| Winiarska-Mieczan, Anna. Cadmium, lead, copper and zinc in breast milk in Poland. Biological trace element research. 2014. 157:36-44 | Pb, Cd | Poland |
| Wongsasuluk, P.; Sematon, S.; Robson, M.; Siriwong, W. Heavy metals levels in breast milk of lactating mothers working in heavy metals contaminated factories. Environment Asia. 2020. 13:99-105 | Pb, Cd | Thailand |
| Xiong, Chan; Stiboller, Michael; Glabonjat, Ronald A; Rieger, Jaqueline; Paton, Lhiam; Francesconi, Kevin A. Transport of arsenolipids to the milk of a nursing mother after consuming salmon fish. Journal of trace elements in medicine and biology : organ of the Society for Minerals and Trace Elements (GMS). 2020. 61:126502 | As | Austria, Norway |
| Xu, Chenye; Tang, Mengling; Zhu, Siyu; Naranmandura, Hua; Liu, Weiping. Assessment of arsenic in colostrum and cord serum and risk exposure to neonates from an island population in China. Environmental science and pollution research international. 2016. 23:22467-22476 | As | China |
| Xu, Peiwei; Zheng, Yibin; Wang, Xiaofeng; Shen, Haitao; Wu, Lizhi; Chen, Yuan; Xu, Dandan; Xiang, Jie; Cheng, Ping; Chen, Zhijian; Lou, Xiaoming. Breastfed infants' exposure to polychlorinated biphenyls, polychlorinated dibenzo-p-dioxins and dibenzofurans, and per- and polyfluoroalkyl substances: A cross-sectional study of a municipal waste incinerator in China. Chemosphere. 2022. 309:136639 | PFAS | China |
| Xu, Yaqi; Sui, Xinyao; Li, Jinhong; Zhang, Liyi; Wang, Pengpeng; Liu, Yang; Shi, Huijing; Zhang, Yunhui. Early-life exposure to per- and polyfluoroalkyl substances: Analysis of levels, health risk and binding abilities to transport proteins. Eco-Environment & Health. 2024. 3:308-316 | PFAS | China |
| Yalcin, S Songul; Yurdakok, Kadriye; Yalcin, Suzan; Engur-Karasimav, Defne; Coskun, Turgay. Maternal and environmental determinants of breast-milk mercury concentrations. The Turkish journal of pediatrics. 2010. 52:9-Jan | Hg | Turkiye |
| Yang, Meng-Tao; ; Yang, Fei; Lan, Qiu-Ye; Huang, Si-Yu; Chen, Xiao-Yan; Qiu, Ying-Zhen; Zhu, Hui-Lian. Concentrations and exposure risks of per- and polyfluoroalkyl substances in human milk from Southern China during the first 400 days of lactation. Ecotoxicology and environmental safety. 2025. 289:117442 | PFAS | China |
| Yao, Jingzhi; Dong, Zhaomin; Jiang, Lulin; Pan, Yitao; Zhao, Meirong; Bai, Xiaoxia; Dai, Jiayin. Emerging and Legacy Perfluoroalkyl Substances in Breastfed Chinese Infants: Renal Clearance, Body Burden, and Implications. Environmental health perspectives. 2023. 131:37003 | PFAS | China |
| Yoshinaga J.; Li J.-Z.; Suzuki T.; Karita K.; Abe M.; Fujii H.; Mishina J.; Morita M. Trace elements in human transitory milk: Variation caused by biological attributes of mother and infant. Biological Trace Element Research. 1991. 31:159-170 | Pb, Cd | Japan |
| Younes, B; Al-Meshari, A A; Al-Hakeem, A; Al-Saleh, S; Al-Zamel, F; Al-Shammari, F; Alwarthan, A. Lead concentration in breast milk of nursing mothers living in Riyadh. Annals of Saudi medicine. 1995. 15:249-51 | Pb | Saudi Arabia |
| Zaldivar R.; Ghai G.L. Clinical epidemiological studies on endemic chronic arsenic poisoning in children and adults, including observations on children with high- and low-intake of dietary arsenic, KLINISCHE, EPIDEMIOLOGISCHE UNTERSUCHUNGEN UEBER EINE ENDEMISCHE, CHRONISCHE ARS. Zentralblatt fur Bakteriologie. Hygiene. Krankenhaushygiene Betriebshygiene Praventive Medizin - Abt. 1 Orig. B. 1980. 170:409-421 | As | Chile |
| Zaldivar, R.; Ghai, G. L. Clinical epidemiological studies on endemic chronic arsenic poisoning in children and adults, including observations on children with high- and low-intake of dietary arsenic. Zentralblatt fur Bakteriologie, 1B. 1980. 170:409-421 | As | Chile |
| Zhang, Chengbin; Luo, Yun; Sun, Shuai; Tian, Tian; Zhu, Meiwen; Ahmad, Zia; Yang, Jiajia; Jin, Jing; Zhang, Haijun; Chen, Jiping; Geng, Ningbo. Accumulation characteristics of metals in human breast milk and association with dietary intake in northeastern China. The Science of the total environment. 2024. 912:168515 | As, Pb, Cd, Hg | China |
| Zhang, Xin; Zhou, Xingyan; Chen, Huijun; Gao, Xinyi; Zhou, Yan; Lee, Hian Kee; Huang, Zhenzhen. Changes in Concentrations of Polyfluoroalkyl Substances in Human Milk Over Lactation Time and Effects of Maternal Exposure via Analysis of Matched Samples. Environmental science & technology. 2024. 58:4115-4126 | PFAS | China |
| Zheng, Guomao; Schreder, Erika; Dempsey, Jennifer C; Uding, Nancy; Chu, Valerie; Andres, Gabriel; Sathyanarayana, Sheela; Salamova, Amina. Per- and Polyfluoroalkyl Substances (PFAS) in Breast Milk: Concerning Trends for Current-Use PFAS. Environmental science & technology. 2021. 55:7510-7520 | PFAS | United States |
| Zheng, Ping; Liu, Yingxue; An, Qi; Yang, Xiaomeng; Yin, Shanshan; Ma, Lena Q; Liu, Weiping. Prenatal and postnatal exposure to emerging and legacy per-/polyfluoroalkyl substances: Levels and transfer in maternal serum, cord serum, and breast milk. The Science of the total environment. 2022. 812:152446 | PFAS | China |
| Zielinska-Pukos, Monika A; Michalska-Kacymirow, Magdalena; Kurek, Eliza; Bulska, Ewa; Grabowicz-Chadrzynska, Iwona; Wesolowska, Aleksandra; Hamulka, Jadwiga. Breastmilk mineral composition among well-educated mothers from Central Poland - Associations with maternal dietary intake, dietary patterns and infant psychomotor development. Journal of trace elements in medicine and biology : organ of the Society for Minerals and Trace Elements (GMS). 2024. 83:127393 | As, Pb, Cd | Poland |

As, arsenic; Cd, cadmium; Hg, mercury; Pb, lead; PFAS, per- and polyfluoroalkyl substances.

**Supplemental Table 8.** Full text articles excluded at confirmatory review for infant formula with reason.^1^

| Article | Reason for exclusion |
| --- | --- |
| Galster, W A. Mercury in Alaskan Eskimo mothers and infants. Environmental Health Perspectives 15 (1976): 135-40 | Outcome |
| Hoffman-Pennesi, D; Winfield, S; Gavelek, A; Santillana Farakos, SM; Spungen, J. Infants' and young children's dietary exposures to lead and cadmium: FDA total diet study 2018-2020. Food Additives & Contaminants: Part A: Chemistry, Analysis, Control, Exposure & Risk Assessment 41 11 (2024):1454-1479 | Outcome |
| Sargent, JD; Dalton, MA; O’Connor, GT; Olmstead, EM; Klein, RZ. Randomized trial of calcium glycerophosphate-supplemented infant formula to prevent lead absorption. The American Journal of Clinical Nutrition 69 (1999): 1224-30 | Outcome |
| Gardener, Hannah; Bowen, Jaclyn; Callan, Sean P. Lead and cadmium contamination in a large sample of United States infant formulas and baby foods. The Science of the Total Environment 651 (2019): 822-827 | Study design |
| Capar, SG; Gajan, RJ; Madzsar, E; Albert, RH; Sanders, M; Zyren, J. Determination of lead and cadmium in foods by anodic stripping voltammetry: II. Collaborative study. Journal of Association of Official Analytical Chemists 65 (1982): 978-86 | Study design |
| Chao, HH; Guo, CH; Huang, CB; Chen, PC; Li, HC; Hsiung, DY; Chou, YK. Arsenic, cadmium, lead, and aluminium concentrations in human milk at early stages of lactation. Pediatrics and Neonatology 55 2 (2014): 127-34 | Identified and reviewed during manual search of included articles; Outcome |
| Khanjani, N.; Jafari, M.; Ahmadi Mousavi, E. Breast milk contamination with lead and cadmium and its related factors in Kerman, Iran. Journal of Environmental Health Science and Engineering 16 (2018): 323–335 | Identified and reviewed during manual search of included articles; Outcome |
| Gyllenhammar, Irina; Benskin, Jonathan P.; Sandblom, Oskar; Berger, Urs; Ahrens, Lutz; Lignell, Sanna; Wiberg, Karin; Glynn, Anders. Perfluoroalkyl Acids (PFAAs) in Serum from 2-4-Month-Old Infants: Influence of Maternal Serum Concentration, Gestational Age, Breast-Feeding, and Contaminated Drinking Water. Environmental Science and Technology 52 12 (2018): 7101-7110 | Identified and reviewed during manual search of included articles; Outcome |
| Chekri, Rachida; Le Calvez, Emilie; Zinck, Julie; Leblanc, J-C.; Sirot, Véronique; Hulin, Marion; Noël, Laurent; Guérin, Thierry. Trace element contents in foods from the first French total diet study on infants and toddlers. Journal of Food Composition and Analysis 78 (2019): 108-120 | Identified and reviewed during manual search of included articles; Population |
| Tozo, M.L.L.; Pinheiro, F.C.; Nóbrega, J.A. Determination of Macro, Micro and Toxic Elements using Argon-Based Plasma Spectroanalytical Methods in order to support Brazilian Regulations on Inorganic Constituents in Infant Foods. Brazilian Journal of Analytic Chemistry 8 31 (2021): 74–88 | Identified and reviewed during manual search of included articles; Population |
| Jallad, KN. The Hazards of a Ubiquitary Metalloid, Arsenic, Hiding in Infant Diets: Detection, Speciation, Exposure, and Risk Assessment. Biological Trace Element Research 190 1 (2019):11-23 | Identified and reviewed during manual search of included articles; Population |
| Pandelova, Marchela; Lopez, Walkiria Levy; Michalke, Bernhard; Schramm, Karl-Werner. Ca, Cd, Cu, Fe, Hg, Mn, Ni, Pb, Se, and Zn contents in baby foods from the EU market: Comparison of assessed infant intakes with the present safety limits for minerals and trace elements. Journal of Food Composition and Analysis 27 2 (2012): 120-127 | Identified and reviewed during manual search of included articles; Population |
| de Paiva, Esther Lima; Morgano, Marcelo Antônio; Arisseto-Bragotto, Adriana Pavesi. Occurrence and determination of inorganic contaminants in baby food and infant formula. Current Opinion in Food Science 30 (2019): 60-66 | Identified and reviewed during manual search of included articles; Publication type |
| Elaridi, Jomana; Dimassi, Hani; Al Yamani, Osama; Estephan, Maria; Hassan, Hussein F. Determination of lead, cadmium and arsenic in infant formula in the Lebanese market. Food Control 123 (2020): 107750 | Identified and reviewed during manual search of included articles; Database duplicate |
| Höpfner, T.; Wollenberg, M.; Jäger A.; Stadion, M.; Jung, C.; Klook, A. L.; Lindtner, O. The contribution of infant formula to the food survey-based dietary exposure of nine selected elements. Journal of Environmental Exposure Assessment 4 (2025): 9 | Identified and reviewed during manual search of Journal of Environmental Exposure Assessment; Study design |

## **Supplemental Table 9.** Study characteristics of infant formula studies published before the year 2000.

| Author, Year | Contaminant(s) | Data Collection | Sampling Methods | Analytical Methods (Critical Appraisal)^1^ | Funding Source |
| --- | --- | --- | --- | --- | --- |
| Baum (1),  1997 | Lead | Boston, Massachusetts (1994-1995) | Convenience sample from 40 infants (<9 months old) from the Boston Children’s Hospital emergency department. Caretakers brought a supply of reconstituted formula from home. | Perkin-Elmer graphite furnace (with platform) atomic absorption spectrophotometer.  (Acceptable) | William F. Milton Fund of Harvard University |
| Thompson (2), 1993 | Lead | Columbus, Ohio^2^ (1992) | Powder, undiluted liquid, and ready to drink formula were used. | Inductively Coupled Plasma/Mass Spectrometry (ICP/MS) instrument (PQ II Plus, Fisons) equipped with an ultrasonic nebulizer (Cetac Technologies, Inc., Model U-5000).  (Acceptable) | Not reported (NR); author affiliation is from Abbott^2^ |
| Munro (3),  1987 | Lead | Las Cruces, New Mexico (NR) | Steel cans of Enfamil concentrated liquid infant formula (Mead Johnson, Evansville, Indiana) randomly obtained from a local retail food store. | Allied Instrumentation Laboratories 457 atomic absorption spectrometer.  (Acceptable) | National Institutes of Health |
| Rabinowitz^3^ (4),  1985 | Lead | Boston, Massachusetts (1979-1981) | From a previous study assessing umbilical cord blood lead, participants from the highest, lowest, and centermost deciles of blood lead were selected. Formula samples with brand name were collected at home in a vial. | Anodic stripping voltammetry (ESA, Bedford. MS, Model 2014). A random portion of samples collected were measured each month due to limited laboratory resources.  (Acceptable) | National Institute of Child Health and Human Development |
| Ryu^3^ (5),  1983 | Lead | Iowa City, Iowa (1975-1976) | Non-descript ready-to-feed formula in weighed glass bottles delivered to families weekly until the infant was 111 days old. Empty, partially empty, and unused bottles were collected and weighed. | Flameless atomic absorption spectrophotometry with a graphite furnace.  (Acceptable) | Public Health Service, Food and Drug Administration |
| Walker (6),  1980 | Lead | Washington, DC^2^ (1978-1979) | Cans with different lots of national brands. | Perkin-Elmer Model 603 atomic absorption spectrophotometer.  (Some concerns) | NR; author affiliation is Environmental Health Administration, Government of the District of Columbia^2^ |
| Lamm (7),  1974 | Lead | New York (1972-1973) | Cans, cartons, and bottles purchased in local retail stores over a few months. | Flameless atomic absorption spectrophotometric method.  (Acceptable) | The John A. Hartford Foundation, Inc., The Charles S. Raizen Fund, and The Association for the Help of Retarded Children, New York City Chapter |
| Lamm (8),  1973 | Lead | Connecticut (1971-1972) | Cans from various lots of major brands purchased in original containers from local retail stores over a few months. | Perkin-Elmer 305 atomic absorption spectrophotometer with a boat attachment.  (Acceptable) | Assistance from the Connecticut State Department of Consumer Affairs and Connecticut State Department of Agriculture |
| Murthy (9),  1971 | Lead  Cadmium | Cincinnati, Ohio area (1968) | 4 different brands collected quarterly from the local market. Each sample represented 3 to 4 cans selected at random, which were mixed before sampling. | Atomic absorption spectrophotometer (Jarrel Ash 2 Model 82-360) equipped with HECTO burner.  (Acceptable) | NR; author affiliation is from Food and Drug Administration^2^ |

Studies are ordered by descending year of publication date. All articles were descriptive, cross-sectional studies except Rabinowitz (4) and Ryu (5) in which we extracted descriptive data from these prospective cohort studies.

^1^A technical expert panel with expertise in human biomonitoring and surveillance of environmental toxicants, specifically for heavy metals and PFAS, critically appraised the sample collection and analytic methods used for measuring contaminants as ‘acceptable’ or ‘some concerns’ due to insufficient reporting of methods or quality control.

ICP/MS, Inductively Coupled Plasma/Mass Spectrometry; NR, not reported; PQ II, PlasmaQuad PQ II.

## **Supplemental Table 10.** Results of infant formula studies published before the year 2000.^1,2^

| Author, Year | Sample Preparation Method Prior to Analysis  (original sample form; dilution source and volume/ratio)^3^ | Type | Cadmium (ng/g, No. detected/ total)^1,3^ | Lead (ng/g, No. detected/ total)^3^ |
| --- | --- | --- | --- | --- |
| Baum (1),  1997 | Form: powdered (n=9 samples) and concentrated (n=31 samples);  Dilution: home tap or bottled water. Dilution volume/ratio not reported (NR); water was boiled in 73% of cases | Reconstituted infant formula (n=31 (77%) were made from concentrate; n=9 (23%) from powder; n=40 different samples; number of unique products unknown) | NA | Range = 13-70,  3/40 |
| Thompson (2), 1993 | Form: powdered;  Dilution: no  Form: concentrated;  Dilution: NR  Form: ready to eat, dehydrated before analysis;  Dilution: NR | Milk-based concentrated liquid 1  Milk-based concentrated liquid 2  Milk-based concentrated liquid 3  Milk-based powder 1  Milk-based powder 2  Milk-based powder 3  Milk-based ready-to-feed 1  Milk-based ready-to-feed 2  Milk-based ready-to-feed 3  Soy-based concentrated liquid 1  Soy-based concentrated liquid 2  Soy-based concentrated liquid 3  Soy-based powder 1  Soy-based powder 2  Soy-based powder 3  Soy-based ready-to-feed 1  Soy-based ready-to-feed 2  Soy-based ready-to-feed 3  (n=18 different products) | NA | 0.48 ± 0.08  0.24 ± 0.06  0.3 ± 0.03  0.24 ± 0.010.9 ± 0.06  0.92 ± 0.01  0.61 ± 0.06  0.42 ± 0.05  1.1 ± 0.1  1.9 ± 0.07  1.06 ± 0.07  NR  1.81 ± 0.08  1.2 ± 0.02  NR  1.78 ± 0.03  1.48 ± 0.05  1.2 ± 0.2,  16/18 |
| Munro (3),  1987 | Form: concentrated liquid;  Dilution: NR  Aqueous fraction analyzed and fat fraction discarded | Enfamil concentrated liquid (Mead Johnson, Evansville, IN) from steel, soldered cans  (n=1 product) | NA | 14 ± 2,  1/1 |
| Rabinowitz (4), 1985 | Form: NR;  Dilution: home water source. Dilution volume/ratio NR; mothers prepared the formula according to their usual practice | Composite (Similac, Enfamil, and Isomil)  (n=73 samples; number of unique products unknown) | NA | 23 ± 3  (mean ± SE),  NR |
| Ryu (5),  1983 | Form: ready to eat;  Dilution: NR | Commercially available milk-based formula from quart cans  Different milk-based and soy protein isolate-based formulas  (n=NR) | NA | 57 parts per billion in formula for older infants  (mean, no variability reported)  20 (range = 19-26) for formula for younger infants, NR |
| Walker Jr. (6), 1980 | Form: NR;  Dilution: NR | Cans of national brands:  Infant formula brand #1  Infant formula brand #2  Infant formula brand #3  Infant formula brand #4  Infant formula brand #5  Infant formula brand #6  Infant formula brand #7  Infant formula brand #8  Infant formula brand #9  Infant formula brand #10  Infant formula brand #11  (n=11 different products) | NA | 50 (range = 30-70)  50 (range = 30-90)  50 (range = 30-70)  50 (range = 10-60)  500 (range = 400-570)  60 (range = 50-90)  70 (range = 50-900)  90 (range = 50-100)  80 (range = 20-90)  80 (range = 70-100)  400 (range = 0-600),  NR |
| Lamm (7),  1974 | Form: concentrate and ready to eat;  Dilution: NR | Composite of 2 brands of infant formula from concentrate  Composite of 2 brands of ready to serve infant formula  (n=4 different products). Packaged in cans or cartons. | NA | 83 ± 9,  2/2  33 ± 4,  NR  (mean ± SE) |
| Lamm (8),  1973 | Form: concentrate;  Dilution: NR | Composite of 5 brands of infant formula concentrate in stiff, seemed cans  (n=5 different products) | NA | 500 (range = 200-1100),  NR |
| Murthy (9),  1971 | Form: NR;  Dilution: source NR, diluted 1:1; timing of dilution NR | Modified milk infant formula 2  Modified milk infant formula 1  Soya infant formula  Lamb meat infant formula  (n=4 different products) | 20 ± 4  21 ± 5  39 ± 7  21 ± 15 | 340 ± 210  330 ± 160  540 ± 110  510 ± 140,  NR |

Values reported as mean ± SD unless noted otherwise.

Studies are ordered by descending year of publication date.

^1^Concentration data were converted, as needed, so that results were reported in the same unit within each contaminant for comparison.

^2^A technical expert panel with expertise in human biomonitoring and surveillance of environmental toxicants, specifically for heavy metals and PFAS, critically appraised the sample collection and analytic methods used for measuring contaminants as ‘acceptable’ except Walker Jr. (6), 1980 that lacked information on quality controls.

NA, not assessed; Ng/g, nanogram per gram; NR, not reported; SE, standard error.

^3^Like manuscript Table 8, the form with dilution and number detected/total were extracted post hoc. The form and dilution were extracted with two reviewers, and the number detected/total was extracted by one reviewer and checked by another.

## **Supplemental Table 11.** Reference list of infant formula articles included in heat map.

| Article | Contaminant(s) assessed | Country |
| --- | --- | --- |
| Abafe, Ovokeroye A; Macheka, Linda R; Olowoyo, Joshua O. Confirmatory Analysis of Per and Polyfluoroalkyl Substances in Milk and Infant Formula Using UHPLC-MS/MS. Molecules (Basel, Switzerland). 2021. 26: | Per- and polyfluoroalkyl substances (PFAS) | South Africa |
| Alharbi, Najla S; Akamsiei, Rawdah M; Almaiman, Lama A; Al-Samti, Mostafa A; Al-Mutairi, Hamad S; Al-Owais, Bandar S; Alkhalaf, Majid M; Bineid, Mohammed A. Occurrence and dietary exposure assessment of heavy metals in baby foods in the Kingdom of Saudi Arabia. Food science & nutrition. 2023. 11:5270-5282 | Arsenic (As), lead (Pb), cadmium (Cd) | Saudi Arabia |
| Alkanani, T.; Friel, J. K.; Jackson, S. E.; Longerich, H. P. Comparison between digestion procedures for the multielemental analysis of milk by inductively coupled plasma mass spectrometry. Journal of Agricultural and Food Chemistry. 1994. 42:1965-1970 | As, Pb, Cd | Canada |
| Al-Khalifa, A. S.; Ahmad, D. Determination of key elements by ICP-OES in commercially available infant formulae and baby foods in Saudi Arabia. African Journal of Food Science. 2010. 4:464-468 | As, Pb, Cd, mercury (Hg) | Saudi Arabia |
| Alvand, N.; Baghdadi, M.; Alimoradi, M.; Marjani, A.; Isfahani, T. M. Metal-phase microextraction (MPME) as a novel solvent-free and green sample preparation technique: determination of cadmium in infant formula and real water samples. Food Analytical Methods. 2022. 15:2048-2058 | Cd | Iran (Islamic Republic of) |
| Arellano, F. E.; Braeuer, S.; Cirelli, A. F.; Goessler, W.; Perez Carrera, A. L. Occurrence of major and trace elements in powdered milk from Argentina. International Journal of Dairy Technology. 2019. 72:643-650 | As, Pb | Argentina |
| Astolfi, Maria Luisa; Marotta, Daniela; Cammalleri, Vittoria; Marconi, Elisabetta; Antonucci, Arianna; Avino, Pasquale; Canepari, Silvia; Vitali, Matteo; Protano, Carmela. Determination of 40 Elements in Powdered Infant Formulas and Related Risk Assessment. International journal of environmental research and public health. 2021. 18: | As, Pb, Cd | Italy |
| Bargellini, Annalisa; Venturelli, Francesco; Casali, Elisabetta; Ferrari, Angela; Marchesi, Isabella; Borella, Paola. Trace elements in starter infant formula: dietary intake and safety assessment. Environmental science and pollution research international. 2018. 25:2035-2044 | Pb, Cd | Italy |
| Basaran, B. An assessment of heavy metal level in infant formula on the market in Turkey and the hazard index. Journal of Food Composition and Analysis. 2022. 105: | As, Pb, Cd, Hg | Turkiye |
| Baum, C R; Shannon, M W. The lead concentration of reconstituted infant formula. Journal of toxicology. Clinical toxicology. 1997. 35:371-5 | Pb | United States |
| Biego, G. H.; Joyeux, M.; Hartemann, P.; Debry, G. Determination of mineral contents in different kinds of milk and estimation of dietary intake in infants. Food Additives and Contaminants. 1998. 15:775-781 | Pb, Cd | France |
| Bloom H.; Lewis I.C.; Noller B.N. Elemental analysis of dried milks used in infant feeding. Australian Paediatric Journal. 1978. 14:259-264 | Pb, Cd | Australia |
| Bogdan, Alexander R; Klos, Kristine S; Greene, Christopher W; Huset, Carin A; Barry, Kitrina M; Goeden, Helen M. Per- and polyfluoroalkyl substances (PFAS) in powdered infant formula: potential exposures and health risks. Journal of Environmental Exposure Assessment. 2024. 3:14 | PFAS | United States |
| Casey, C E. The content of some trace elements in infant milk foods and supplements available in New Zealand. The New Zealand medical journal. 1977. 85:275-8 | Pb, Cd | New Zealand |
| Chajduk E.; Polkowska-Motrenko H. Application of ICP-MS, INAA and RNAA to the determination of some "difficult" elements in infant formulas. Journal of Radioanalytical and Nuclear Chemistry. 2017. 311:1347-1353 | As | Poland |
| Chajduk, Ewelina; Polkowska-Motrenko, Halina. The use of HPLC-NAA and HPLC-ICP-MS for the speciation of As in infant food. Food chemistry. 2019. 292:129-133 | As | Poland |
| Chajduk, Ewelina; Pyszynska, Marta; Polkowska-Motrenko, Halina. Determination of Trace Elements in Infant Formulas Available on Polish Market. Biological trace element research. 2018. 186:589-596 | As, Pb, Cd | Poland |
| Cordeiro F.; Baer I.; Robouch P.; Emteborg H.; Can S.Z.; Krata A.; Zampella M.; Quetel C.R.; Hearn R.; De la Calle B. Setting maximum limits for trace elements in baby food in European legislation: The outcome of International Measurement Evaluation Programme-33. Food Additives and Contaminants - Part A. 2013. 30:678-686 | Pb, Cd | Belgium |
| Cortes, E.; Gras, N.; Munoz, L.; Cassorla, V. A study of some trace elements in infant foods. Journal of Radioanalytical Chemistry. 1982. 69:401-415 | As | Chile |
| Dabeka, R W. Collaborative study of a graphite-furnace atomic-absorption screening method for the determination of lead in infant formulas. The Analyst. 1984. 109:1259-63 | Pb | Canada |
| Dabeka, R W. Graphite-furnace atomic absorption spectrometric determination of lead, cadmium, cobalt and nickel in infant formulas and evaporated milks after nitric-perchloric acid digestion and coprecipitation with ammonium pyrrolidine dithiocarbamate. The Science of the total environment. 1989. 89:271-7 | Pb, Cd | Canada |
| Dabeka, R W. Survey of lead, cadmium, cobalt and nickel in infant formulas and evaporated milks and estimation of dietary intakes of the elements by infants 0-12 months old. The Science of the total environment. 1989. 89:279-89 | Pb, Cd | Canada |
| Dabeka, R W; McKenzie, A D. Lead and cadmium levels in commercial infant foods and dietary intake by infants 0-1 year old. Food additives and contaminants. 1988. 5:333-42 | Pb, Cd | Canada |
| Dabeka, R. W. Workshop on the determination of lead in foods by graphite furnace atomic absorption spectrometry: preliminary report. Journal of the Association of Official Analytical Chemists. 1982. 65:1005-1009 | Pb | Canada |
| Dabeka, Robert W; McKenzie, Arthur D. Survey of total mercury in infant formulae and oral electrolytes sold in Canada. Food additives & contaminants. Part B, Surveillance. 2012. 5:65-9 | Hg | Canada |
| Dabeka, Robert; Fouquet, Andre; Belisle, Stephane; Turcotte, Stephane. Lead, cadmium and aluminum in Canadian infant formulae, oral electrolytes and glucose solutions. Food additives & contaminants. Part A, Chemistry, analysis, control, exposure & risk assessment. 2011. 28:744-53 | Pb, Cd | Canada |
| de Almeida, Cristine Couto; Baiao, Diego Dos Santos; Rodrigues, Paloma de Almeida; Saint'Pierre, Tatiana Dillenburg; Hauser-Davis, Rachel Ann; Leandro, Katia Christina; Paschoalin, Vania Margaret Flosi; da Costa, Marion Pereira; Conte-Junior, Carlos Adam. Toxic Metals and Metalloids in Infant Formulas Marketed in Brazil, and Child Health Risks According to the Target Hazard Quotients and Target Cancer Risk. International journal of environmental research and public health. 2022. 19: | As, Pb, Cd, Hg | Brazil |
| De Castro, Clarissa S P; Arruda, Andrea F; Da Cunha, Leandro R; SouzaDe, Jurandir R; Braga, Jez W B; Dorea, Jose G. Toxic metals (Pb and Cd) and their respective antagonists (Ca and Zn) in infant formulas and milk marketed in Brasilia, Brazil. International journal of environmental research and public health. 2010. 7:4062-77 | Pb, Cd | Brazil |
| Dehcheshmeh, B. K.; Shakerian, A.; Rahimi, E. Evaluation of aflatoxin M1 and heavy metal in raw materials and infant formula produced in Pegah Dairy Plants, IRAN. Journal of Chemical Health Risks. 2021. 11:55-62 | Pb, Cd | Iran (Islamic Republic of) |
| Demir, Tugba; Agaoglu, Sema. Estimated daily intake and health risk assessment of toxic elements in infant formulas. The British journal of nutrition. 2023. 130:1732-1742 | As, Pb, Cd, Hg | Turkiye |
| Drasch, G; Aigner, S; Roider, G; Staiger, F; Lipowsky, G. Mercury in human colostrum and early breast milk. Its dependence on dental amalgam and other factors. Journal of trace elements in medicine and biology : organ of the Society for Minerals and Trace Elements (GMS). 1998. 12:23-7 | Hg | Germany |
| Dubascoux, Stephane; Nicolas, Marine; Rime, Celine Fragniere; Payot, Janique Richoz; Poitevin, Eric. Simultaneous Determination of 10 Ultratrace Elements in Infant Formula, Adult Nutritionals, and Milk Products by ICP/MS After Pressure Digestion: Single-Laboratory Validation. Journal of AOAC International. 2015. 98:953-61 | As, Pb, Cd, Hg | Switzerland |
| Eklund, G; Oskarsson, A. Exposure of cadmium from infant formulas and weaning foods. Food additives and contaminants. 1999. 16:509-19 | Cd | Sweden |
| Elaridi, J.; Dimassi, H.; Al-Yamani, O.; Estephan, M.; Hassan, H. F. Determination of lead, cadmium and arsenic in infant formula in the Lebanese market. Food Control. 2021. 123: | As, Pb, Cd | Lebanon |
| Esteve, M J; Farre, R; Frigola, A. Determination of Cd, Cu, Pb and Zn content of infant formulas by differential pulse anodic stripping voltammetry (DPASV). Die Nahrung. 1994. 38:386-92 | Pb, Cd | Spain |
| Flores-Aguilar, J. F.; Islas, G.; Rodriguez, J. A.; Paez-Hernandez, M. E.; Galan-Vidal, C. A.; Ibarra, I. S. Selective Pb(II)-imprinted polymer for solid phase extraction in the trace determination of lead in infant formula by capillary electrophoresis. Journal of the Mexican Chemical Society. 2022. 66:221-236 | Pb | Mexico |
| Fujii, Yukiko; Yan, Junxia; Harada, Kouji H; Hitomi, Toshiaki; Yang, Hyeran; Wang, Peiyu; Koizumi, Akio. Levels and profiles of long-chain perfluorinated carboxylic acids in human breast milk and infant formulas in East Asia. Chemosphere. 2012. 86:315-21 | PFAS | China, Japan, Korea (Republic of) |
| Ghuniem, M. M.; Khorshed, M. A.; Khalil, M. M. H. Determination of some essential and toxic elements composition of commercial infant formula in the Egyptian market and their contribution to dietary intake of infants. International Journal of Environmental Analytical Chemistry. 2020. 100:525-548 | Pb, Cd | Egypt |
| Gomez-Nieto, B.; Motyzhov, V.; Gismera, M. J.; Procopio, J. R.; Sevilla, M. T. Fast-sequential determination of cadmium and copper in milk powder and infant formula by direct solid sampling high-resolution continuum source graphite furnace atomic absorption spectrometry. Microchemical Journal. 2020. 159: | Cd | Spain |
| Gulson, B L; Jameson, C W; Mahaffey, K R; Mizon, K J; Patison, N; Law, A J; Korsch, M J; Salter, M A. Relationships of lead in breast milk to lead in blood, urine, and diet of the infant and mother. Environmental health perspectives. 1998. 106:667-74 | Pb | Australia |
| Gulson, B L; Mizon, K J; Korsch, M J; Mahaffey, K R; Taylor, A J. Dietary intakes of selected elements from longitudinal 6-day duplicate diets for pregnant and nonpregnant subjects and elemental concentrations of breast milk and infant formula. Environmental research. 2001. 87:160-74 | Pb | Australia |
| Gulson, B L; Mizon, K J; Palmer, J M; Patison, N; Law, A J; Korsch, M J; Mahaffey, K R; Donnelly, J B. Longitudinal study of daily intake and excretion of lead in newly born infants. Environmental research. 2001. 85:232-45 | Pb | Australia |
| Gundacker, C; Pietschnig, B; Wittmann, K J; Lischka, A. Human milk mercury (Hg) and lead (Pb) levels in Vienna. Advances in experimental medicine and biology. 2000. 478:387-8 | Pb, Hg | Austria |
| Gundacker, Claudia; Pietschnig, Beate; Wittmann, Karl J; Lischka, Andreas; Salzer, Hans; Hohenauer, Leonhard; Schuster, Ernst. Lead and mercury in breast milk. Pediatrics. 2002. 110:873-8 | Pb, Hg | Austria |
| Gundacker, Claudia; Pietschnig, Beate; Wittmann, Karl J; Salzer, Hans; Stoger, Helmut; Reimann-Dorninger, Gerda; Schuster, Ernst; Lischka, Andreas. Smoking, cereal consumption, and supplementation affect cadmium content in breast milk. Journal of exposure science & environmental epidemiology. 2007. 17:39-46 | Cd | Austria |
| Hafez, Lamia M; Kishk, Amal M. Level of lead and cadmium in infant formulae. The Journal of the Egyptian Public Health Association. 2008. 83:285-93 | Pb, Cd | Egypt |
| Hanning, Rhona M; Sandhu, Ranjit; MacMillan, Angus; Moss, Lorraine; Tsuji, Leonard J S; Nieboer, Evert. Impact on blood Pb levels of maternal and early infant feeding practices of First Nation Cree in the Mushkegowuk Territory of northern Ontario, Canada. Journal of environmental monitoring : JEM. 2003. 5:241-5 | Pb | Canada |
| Haschke, F; Steffan, I; Schilling, R; Schuster, E; Salzer, H P. Lead content of water and of reconstituted infant formula in Vienna. Mineral and electrolyte metabolism. 1985. 11:45-51 | Pb | Austria |
| He Dong. Simultaneous determination of sixteen industrial pollutants in infant formula milk powder by dispersive solid phase extraction coupled with ultra-high performance liquid chromatography-tandem mass spectrometry. Analytical Methods. 2017. 9:2561-2569 | PFAS | China |
| Ibrahim, A. S.; Saad, M. F.; Hafiz, N. M. Toxic elements in dried milk and evaluation of their dietary intake in infant formula. International Journal of Veterinary Science. 2020. 9:563-567 | As, Pb, Cd, Hg | Egypt |
| Ikem A.; Nwankwoala A.; Odueyungbo S.; Nyavor K.; Egiebor N. Levels of 26 elements in infant formula from USA, UK, and Nigeria by microwave digestion and ICP-OES. Food Chemistry. 2002. 77:439-447 | As, Pb, Cd, Hg | United Kingdom, United States |
| Jackson, Brian P; Taylor, Vivien F; Punshon, Tracy; Cottingham, Kathryn L. Arsenic concentration and speciation in infant formulas and first foods. Pure and applied chemistry. Chimie pure et appliquee. 2012. 84:215-223 | As | United States |
| Jannat B.; Sadeghi N.; Oveisi M.R.; Behfar A.-A.; Komeilizadeh H.; Shafaati A. Simultaneous determination of lead, cadmium, copper and zinc in infant formula by anodic stripping voltammetry. Iranian Journal of Pharmaceutical Research. 2009. 8:159-162 | Pb, Cd | Iran (Islamic Republic of) |
| Jurowski K.; Krosniak M.; Folta M.; Cole M.; Piekoszewski W. The toxicological analysis of lead and cadmium in prescription food for special medical purposes and modified milk products for newborns and infants available in Polish pharmacies. Journal of Trace Elements in Medicine and Biology. 2019. 51:73-78 | Pb, Cd | Poland |
| Kadac-Czapska, K.; Rutkowska, M.; Knez, E.; Konieczka, P.; Grembecka, M. Non-carcinogenic human risk assessment of the consumption of infant formulas contaminated with total and organic mercury. Journal of Food Composition and Analysis. 2024. 125: | Hg | Poland |
| Kiani A.; Arabameri M.; Moazzen M.; Shariatifar N.; Aeenehvand S.; Khaniki G.J.; Abdel-Wahhab M.; Shahsavari S. Probabilistic Health Risk Assessment of Trace Elements in Baby Food and Milk Powder Using ICP-OES Method. Biological Trace Element Research. 2022. 200:2486-2497 | As, Pb, Cd, Hg | Iran (Islamic Republic of) |
| KIlIc, S.; Tongur, T.; KIlIc, M.; Erkaymaz, T. Determination of some endocrine-disrupting metals and organochlorinated pesticide residues in baby food and infant formula in Turkish markets. Food Analytical Methods. 2018. 11:3352-3361 | As, Pb, Cd | Turkiye |
| Kirkpatrick, D. C.; Conacher, H. B. S.; Meranger, J. C.; Dabeka, R.; Collins, B.; McKenzie, A. D.; Lacroix, G. M. A.; Savary, G. The trace element content of Canadian baby foods and estimation of trace element intake by infants. Canadian Institute of Food Science and Technology Journal. . 13:154-161 | Pb, Cd | Canada |
| Kovar, I Z; Strehlow, C D; Richmond, J; Thompson, M G. Perinatal lead and cadmium burden in a British urban population. Archives of disease in childhood. 1984. 59:36-9 | Pb, Cd | United Kingdom |
| Krachler, M.; Rossipal, E.; Irgolic, K. J. Trace elements in formulas based on cow and soy milk and in Austrian cow milk determined by inductively coupled plasma mass spectrometry. Biological Trace Element Research. 1998. 65:53-74 | Pb, Cd | Austria |
| Krachler, M; Prohaska, T; Koellensperger, G; Rossipal, E; Stingeder, G. Concentrations of selected trace elements in human milk and in infant formulas determined by magnetic sector field inductively coupled plasma-mass spectrometry. Biological trace element research. 2000. 76:97-112 | As | Austria |
| Krachler, M; Rossipal, E. Concentrations of trace elements in extensively hydrolysed infant formulae and their estimated daily intakes. Annals of nutrition & metabolism. 2000. 44:68-74 | Pb, Cd | Austria |
| Kuznetsova, I.; Krusir, G.; Garkovich, O.; Kovalenko, I. Heavy metals in milk infant formulas. Food Science and Technology. 2019. 13:93-99 | Pb | Belarus, Germany, Poland, Switzerland, Ukraine |
| Lamm, S H; Rosen, J F. Lead contamination in milks fed to infants: 1972-1973. Pediatrics. 1974. 53:137-41 | Pb | United States |
| Lamm, S; Cole, B; Glynn, K; Ullmann, W. Lead content of milks fed to infants--1971-1972. The New England journal of medicine. 1973. 289:574-5 | Pb | United States |
| Lankova, Darina; Lacina, Ondrej; Pulkrabova, Jana; Hajslova, Jana. The determination of perfluoroalkyl substances, brominated flame retardants and their metabolites in human breast milk and infant formula. Talanta. 2013. 117:318-25 | PFAS | Czechia |
| Liao K.-W.; ; Lee W.-J.; Chin W.-S. Probabilistic risk assessment for determining non-essential metals in commercial infant formula products in Taiwan. Environmental Epidemiology. 2024. 8:5EP - 6 | As, Pb, Cd | China |
| Liao, Kai-Wei; Lee, Wei-Ju; Lin, Shao-Chi; Tsao, Yi-Ning; Lin, Hsin-Ying; Liu, Chen; Chin, Wei-Shan. Probabilistic risk assessment for determining nonessential metals in commercial infant formula products in Taiwan. Journal of food science. 2024. 89:1804-1813 | As, Pb, Cd | China |
| Lin, Xiaoying; Wu, Xiaoling; Li, Xiangyu; Zhang, Duo; Zheng, Quanzhi; Xu, Jiayi; Lu, Shaoyou. Infant exposure to trace elements in breast milk, infant formulas and complementary foods from southern China. The Science of the total environment. 2022. 838:156597 | As, Pb, Cd | China |
| Ljung, Karin; Palm, Brita; Grander, Margaretha; Vahter, Marie. High concentrations of essential and toxic elements in infant formula and infant foods - A matter of concern. Food chemistry. 2011. 127:943-51 | As, Pb, Cd | Sweden |
| Llorca, Marta; Farre, Marinella; Pico, Yolanda; Teijon, Marisa Lopez; Alvarez, Juan G; Barcelo, Damia. Infant exposure of perfluorinated compounds: levels in breast milk and commercial baby food. Environment international. 2010. 36:584-92 | PFAS | Spain |
| Macheka, Linda R; Olowoyo, Joshua O; Mugivhisa, Liziwe L; Abafe, Ovokeroye A. Determination and assessment of human dietary intake of per and polyfluoroalkyl substances in retail dairy milk and infant formula from South Africa. The Science of the total environment. 2021. 755:142697 | PFAS | South Africa |
| Mandia, Natalia; Bermejo-Barrera, Pilar; Herbello, Paloma; Lopez-Suarez, Olalla; Fraga, Jose M; Fernandez-Perez, Cristina; Couce, Maria L. Human Milk Concentrations of Minerals, Essential and Toxic Trace Elements and Association with Selective Medical, Social, Demographic and Environmental Factors. Nutrients. 2021. 13: | As, Pb, Cd, Hg | Spain |
| Mania, M.; Wojciechowska-Mazurek, M.; Starska, K.; Rebeniak, M.; Szynal, T.; Strzelecka, A.; Postupolski, J. Toxic elements in commercial infant food, estimated dietary intake, and risk assessment in Poland. Polish Journal of Environmental Studies. 2015. 24:2525-2536 | As, Pb, Cd, Hg | Poland |
| Marques, Montse; Correig, Eudald; Capdevila, Esther; Gargallo, Eva; Gonzalez, Neus; Nadal, Marti; Domingo, Jose L. Essential and Non-essential Trace Elements in Milks and Plant-Based Drinks. Biological trace element research. 2022. 200:4524-4533 | Pb, Hg | Spain |
| Martinez, Mari Angeles; Castro, Irma; Rovira, Joaquim; Ares, Susana; Rodriguez, Juan Miguel; Cunha, Sara Cristina; Casal, Susana; Fernandes, Jose Oliveira; Schuhmacher, Marta; Nadal, Marti. Early-life intake of major trace elements, bisphenol A, tetrabromobisphenol A and fatty acids: Comparing human milk and commercial infant formulas. Environmental research. 2019. 169:246-255 | As, Pb, Cd, Hg | Spain |
| Martins, Carla; Vasco, Elsa; Paixao, Eleonora; Alvito, Paula. Total mercury in infant food, occurrence and exposure assessment in Portugal. Food additives & contaminants. Part B, Surveillance. 2013. 6:151-7 | Hg | Portugal |
| Maruszewska, Agnieszka; Zwierello, Wojciech; Skorka-Majewicz, Marta; Baranowska-Bosiacka, Irena; Wszolek, Agata; Janda, Katarzyna; Kulis, Daria; Kapczuk, Patrycja; Chlubek, Dariusz; Gutowska, Izabela. Modified Baby Milk-Bioelements Composition and Toxic Elements Contamination. Molecules (Basel, Switzerland). 2021. 26: | Pb | Poland |
| Massadeh, Adnan Mohammad; ; Alzarieni, Kawthar Zeyad; Alzaghol, Hebah Ziyad; Smadi, Mahmoud Mustafa. Occurrence and health risk assessment of selected metals in commercially available infant formulas in Jordan. Journal of trace elements in medicine and biology : organ of the Society for Minerals and Trace Elements (GMS). 2025. 87:127585 | Pb, Cd | Jordan |
| Meli M.A.; Desideri D.; Sisti D.; Fagiolino I.; Roselli C. Chemical characterization of baby food consumed in Italy. PLoS ONE. 2024. 19:e0297158 | As, Hg | Italy |
| Miclean, M.; Cadar, O.; Roman, C.; Tanaselia, C.; Stefanescu, L.; Stezar, C. I.; Groza, I. S. The influence of environmental contamination on heavy metals and organochlorine compounds levels in milk. Environmental Engineering and Management Journal. 2011. 10:37-42 | Pb, Cd | Romania |
| Mikolajczyk S.; Warenik-Bany M.; Pajurek M. Infant formula and baby food as a source of perfluoroalkyl substances for infants. Environmental Pollution. 2023. 317:120810 | PFAS | Germany, Poland |
| Moreno-Rojas, R; Sanchez-Segarra, P J; Amaro-Lopez, M A; Zurera-Cosano, G. Cadmium content in infant formulas. Toxicological evaluation. Die Nahrung. 2001. 45:357-9 | Cd | Spain |
| Moreno-Rojas, R; Sanchez-Segarra, P J; Canal-Ruiz, C; Amaro-Lopez, M A; Zurera-Cosano, G. Lead content in Spanish market infant formulas and toxicological contribution. Food additives and contaminants. 2002. 19:241-5 | Pb | Spain |
| Muller, J.; Schmidt, E. H. F. Heavy metals in the infant diet. Health evaluation of heavy metals in infant formula and junior food. 1983. :12-Jan | Pb, Cd, Hg | Germany |
| Munro, J. L. Determination of six elements in infant formula by flame and furnace atomic adsorption spectroscopy. Atomic Spectroscopy. 1987. 8:92-95 | Pb | United States |
| Murthy, G K; Rhea, U S. Cadmium, copper, iron, lead, manganese, and zinc in evaporated milk, infant products, and human milk. Journal of dairy science. 1971. 54:1001-5 | Pb, Cd | United States |
| Navarro-Blasco, I; Alvarez-Galindo, J I. Lead levels in retail samples of Spanish infant formulae and their contribution to dietary intake of infants. Food additives and contaminants. 2005. 22:726-34 | Pb | Spain |
| Nookabkaew, S; Rangkadilok, N; Akib, C A; Tuntiwigit, N; Saehun, J; Satayavivad, J. Evaluation of trace elements in selected foods and dietary intake by young children in Thailand. Food additives & contaminants. Part B, Surveillance. 2013. 6:55-67 | As, Pb, Cd | Australia, Germany, Ireland, Malaysia, Netherlands, New Zealand, Philippines, Singapore, Spain, Thailand |
| Nuti, Francesca; ; Fernandez, Feliciana Real; Severi, Mirko; Traversi, Rita; Fanos, Vassilios; Street, Maria Elisabeth; Palanza, Paola; Rovero, Paolo; Papini, Anna Maria. Study of Endocrine-Disrupting Chemicals in Infant Formulas and Baby Bottles: Data from the European LIFE-MILCH PROJECT. Molecules (Basel, Switzerland). 2024. 29: | PFAS | Italy |
| Olu-Owolabi, B. I.; Fakayode, S. O.; Adebowale, K. O.; Onianwa, P. C. Proximate and elemental composition and their estimated daily intake in infant formulae from developed and developing countries: a comparative analysis. Journal of Food, Agriculture & Environment. 2007. 5:40-44 | As, Pb, Cd | Denmark, France, Germany, Italy, South Africa, United States, Venezuela (Bolivarian Rep. of) |
| Pandelova, M.; Lopez, W. L.; Michalke, B.; Schramm, K. W. Ca, Cd, Cu, Fe, Hg, Mn, Ni, Pb, Se, and Zn contents in baby foods from the EU market: comparison of assessed infant intakes with the present safety limits for minerals and trace elements. Journal of Food Composition and Analysis. 2012. 27:120-127 | Pb, Cd, Hg | France, Germany, Italy, Portugal, Slovakia, Spain, Sweden, United Kingdom |
| Pasecnaja E.; Bartkevics V.; Zacs D. Occurrence of perfluoroalkyl compounds in commercially available baby food produced in different European countries - levels, dietary intake, and exposure assessment. Chemosphere. 2024. 364:143074 | PFAS | Multiple European countries, not described |
| Pecev-Marinkovic, Emilija; Rasic Misic, Ivana; Mrmosanin, Jelena; Petrovic, Stefan; Pavlovic, Aleksandra; Tosic, Snezana. Quantitative determination of selected elements in infant baby formulae and baby food cereals commercially available in Serbia using the ICP OES method. Journal of trace elements in medicine and biology : organ of the Society for Minerals and Trace Elements (GMS). 2024. 84:127457 | As, Pb, Cd, Hg | Serbia |
| Peirovi-Minaee, Roya; Taghavi, Mahmoud; Harimi, Marzieh; Zarei, Ahmad. Trace elements in commercially available infant formulas in Iran: Determination and estimation of health risks. Food and chemical toxicology : an international journal published for the British Industrial Biological Research Association. 2024. 186:114588 | As, Pb, Cd | Iran (Islamic Republic of) |
| Pietrzak-Fiecko, R.; Smoczynski, S. S. Cadmium and lead contents in human milk, cow's milk and infant formulas. Polish Journal of Natural Sciences. 2006. 21:1105-1112 | Pb, Cd | Poland |
| Rabinowitz, M; Leviton, A; Needleman, H. Lead in milk and infant blood: a dose-response model. Archives of environmental health. 1985. 40:283-6 | Pb | United States |
| Raie R.M.; Smith H. Trace element deficiency and cot deaths. Medicine, Science and the Law. 1981. 21:41-46 | As, Hg | United Kingdom |
| Richmond, J; Strehlow, C D; Chalkley, S R. Dietary intake of Al, Ca, Cu, Fe, Pb, and Zn in infants. British journal of biomedical science. 1993. 50:178-86 | Pb | United Kingdom |
| Rivero Martino, F. A.; Fernandez Sanchez, M. L.; Sanz-Medel, A. The potential of double focusing-ICP-MS for studying elemental distribution patterns in whole milk, skimmed milk and milk whey of different milks. Analytica Chimica Acta. 2001. 442:191-200 | Pb, Cd, Hg | Spain |
| Rodriguez Rodriguez, E. M.; Delgado Uretra, E.; Diaz Romero, C. Concentrations of cadmium and lead in different types of milk. Zeitschrift fur Lebensmittel-Untersuchung und -Forschung. A, Food Research and Technology. 1999. 208:162-168 | Pb, Cd | Spain |
| Rye, J E; Ziegler, E E; Nelson, S E; Fomon, S J. Dietary intake of lead and blood lead concentration in early infancy. American journal of diseases of children (1960). 1983. 137:886-91 | Pb | United States |
| Sadeghi, Naficeh; Oveisi, Mohammad Reza; Jannat, Behrooz; Hajimahmoodi, Mannan; Behfar, Abdolazim; Behzad, Masoomeh; Norouzi, Narges; Oveisi, Morvarid; Jannat, Behzad. Simultaneous Measurement of Zinc, Copper, Lead and Cadmium in Baby Weaning Food and Powder Milk by DPASV. Iranian journal of pharmaceutical research : IJPR. 2014. 13:345-9 | Pb, Cd | Iran (Islamic Republic of) |
| Sipahi, Hande; Eken, Ayse; Aydin, Ahmet; Sahin, Gonul; Baydar, Terken. Safety assessment of essential and toxic metals in infant formulas. The Turkish journal of pediatrics. 2014. 56:385-91 | Pb, Cd | Turkiye |
| Sola-Larranaga C.; Navarro-Blasco I. Preliminary chemometric study of minerals and trace elements in Spanish infant formulae. Analytica Chimica Acta. 2006. 555:354-363 | Pb, Cd | Spain |
| Sorbo, A.; Ciprotti, M.; Colabucci, A.; Zoani, C.; Gregorio, M. di; Turco, A. C.; Ciaralli, L. Preparation of an infant formula proficiency testing material and assessment of its homogeneity and stability. Accreditation and Quality Assurance. 2015. 20:373-380 | Pb, Cd | Italy |
| Sorbo, A.; Turco, A. C.; Gregorio, M. di; Ciaralli, L. Development and validation of an analytical method for the determination of arsenic, cadmium and lead content in powdered infant formula by means of quadrupole Inductively Coupled Plasma Mass Spectrometry. Food Control. 2014. 44:159-165 | As, Pb, Cd | Italy |
| Souza, Marilia Cristina Oliveira; Souza, Juliana Maria Oliveira; da Costa, Bruno Ruiz Brandao; Gonzalez, Neus; Rocha, Bruno Alves; Cruz, Jonas Carneiro; Guida, Yago; Souza, Vanessa Cristina Oliveira; Nadal, Marti; Domingo, Jose L; Barbosa, Fernando. Levels of organic pollutants and metals/metalloids in infant formula marketed in Brazil: Risks to early-life health. Food research international (Ottawa, Ont.). 2023. 174:113594 | As, Pb, Cd | Brazil |
| Su, Chuanyou; Zheng, Nan; Gao, Yanan; Huang, Shengnan; Yang, Xue; Wang, Ziwei; Yang, Hongjian; Wang, Jiaqi. Content and Dietary Exposure Assessment of Toxic Elements in Infant Formulas from the Chinese Market. Foods (Basel, Switzerland). 2020. 9: | As, Pb, Cd | China |
| Tahboub, Yahya R; Massadeh, Adnan M; Al-Sheyab, Nihaya A; El Shrafat, Diab; Nsserat, Israa A. Levels of Trace Elements in Human Breast Milk in Jordan: a Comparison with Infant Formula Milk Powder. Biological trace element research. 2021. 199:4066-4073 | As, Pb, Cd | Jordan |
| Tao, Lin; Ma, Jing; Kunisue, Tatsuya; Libelo, E Laurence; Tanabe, Shinsuke; Kannan, Kurunthachalam. Perfluorinated compounds in human breast milk from several Asian countries, and in infant formula and dairy milk from the United States. Environmental science & technology. 2008. 42:8597-602 | PFAS | Indonesia, Japan, Malaysia, Philippines, United States, Viet Nam |
| TatahMentan M.; Nyachoti S.; Godebo T.R. Elemental composition of toxic and essential elements in rice-based baby foods from the United States and other countries: A probabilistic risk analysis. Food and Chemical Toxicology. 2024. 188:114677 | As, Pb, Cd | United States |
| Tatsuta N.; Asato K.; Iwai-Shimada M.; Iwai K.; Nakayama S.F.; Yamazaki S.; Nakai K. Dietary intake of methylmercury by 0-5 years children using the duplicate diet method in Japan. Environmental Health and Preventive Medicine. 2024. 29:27 | Hg | Japan |
| Thompson, J J. Determination of ultratrace levels of lead in infant formula by isotope dilution inductively coupled plasma mass spectrometry. Journal of AOAC International. 1993. 76:1378-84 | Pb | United States |
| Tiran B.; Rossipal E.; Tiran A.; Karpf E.; Lorenz O. Burden of cadmium and lead content in human milk and milk formulas in Styria, Austria. A moderate industrialized area. Trace Elements in Medicine. 1994. 11:42-45 | Pb, Cd | Austria |
| Ursinyova, M.; Hladikova, V. The intake of selected toxic elements from milk in infants. Fresenius Environmental Bulletin. 1997. 6:627-632 | Pb, Cd, Hg | Slovakia |
| Ursinyova, Monika; Masanova, Vlasta. Cadmium, lead and mercury in human milk from Slovakia. Food additives and contaminants. 2005. 22:579-89 | Pb, Cd, Hg | Slovakia |
| van Beijsterveldt, Inge A L P; van Zelst, Bertrand D; de Fluiter, Kirsten S; van den Berg, Sjoerd A A; van der Steen, Manouk; Hokken-Koelega, Anita C S. Poly- and perfluoroalkyl substances (PFAS) exposure through infant feeding in early life. Environment international. 2022. 164:107274 | PFAS | Netherlands |
| Vela, Nohora P; Heitkemper, Douglas T. Total arsenic determination and speciation in infant food products by ion chromatography-inductively coupled plasma-mass spectrometry. Journal of AOAC International. 2004. 87:244-52 | As | United States |
| Walker Jr. B. Lead content of milk and infant formula. Journal of Food Protection. 1980. 43:178-179 | Pb | United States |
| Winiarska-Mieczan, A. Assessment of infant exposure to lead and cadmium content in infant formulas. Journal of Elementology. 2009. 14:573-581 | Pb, Cd | Poland |
| Wojciechowska-Mazurek, M.; Karlowski, K.; Kumpulainen, J.; Starska, K.; Brulinska-Ostrowska, E.; Zwiek-Ludwicka, K. Lead, calcium, mercury, copper and zinc content in Polish powdered milk and products for infants and children. Natural antioxidants and food quality in atherosclerosis and cancer prevention. 1996. :423-431 | Pb, Cd, Hg | Poland |
| Yin, Yaqi; Fan, Chen; Cheng, Linru; Shan, Yuwei. Deep eutectic solvent-functionalized amorphous UiO-66 for efficient extraction and ultrasensitive analysis of perfluoroalkyl substances in infant milk powder. Food chemistry: X. 2024. 23:101555 | PFAS | Not described |
| Zabaleta, I.; Blanco-Zubiaguirre, L.; Baharli, E. N.; Olivares, M.; Prieto, A.; Zuloaga, O.; Elizalde, M. P. Occurrence of per- and polyfluorinated compounds in paper and board packaging materials and migration to food simulants and foodstuffs. Food Chemistry. 2020. 321: | PFAS | Spain |

As, arsenic; Cd, cadmium; Hg, mercury; Pb, lead; PFAS, per- and polyfluoroalkyl substances.

## **Supplemental Table 12.** Details of infant formula products as described by authors of the United States studies.

| Author, year | Contaminant assessed | Infant formula description |
| --- | --- | --- |
| Baum (1), 1997 | Lead (Pb) | Reconstituted infant formula (77% were made from concentrate; 23% from powder) |
| Bogdan (10), 2024 | Perfluorooctane sulfonate (PFOS) | Bobbie Organic Infant Formula Dairy Standard, powdered |
| Bogdan (10), 2024 | PFOS | Earth’s Best Organic Dairy Infant Formula Dairy Standard, powdered |
| Bogdan (10), 2024 | PFOS | Earth’s Best Non-GMO Soy Infant Formula Soy Dairy/Lactose intolerance and Galactosemia, powdered |
| Bogdan (10), 2024 | PFOS | Enfamil Gentlease Dairy Lactose sensitivity, powdered |
| Bogdan (10), 2024 | PFOS | Enfamil A.R. Dairy Added rice to prevent spit-up, powdered |
| Bogdan (10), 2024 | PFOS | Enfamil EnfaCare NeuroPro Dairy Premature infants, powdered |
| Bogdan (10), 2024 | PFOS | Enfamil Nutramigen Dairy Hypoallergenic; amino acid (hydrolyzed milk protein), powdered |
| Bogdan (10), 2024 | PFOS | Gerber Good Start GentlePro Non-GMO Dairy Protein sensitivity, powdered |
| Bogdan (10), 2024 | PFOS | Gerber Good Start State 1 Soy Non-GMO Soy Dairy/Lactose intolerance, powdered |
| Bogdan (10), 2024 | PFOS | HappyBaby Organic Infant Formula Dairy Standard, powdered |
| Bogdan (10), 2024 | PFOS | Nutricia Neocate Amino Acid Hypoallergenic; amino acid (made from individual amino acids rather than hydrolyzed casein from milk), powdered |
| Bogdan (10), 2024 | PFOS | Similac Advance Dairy Standard, powdered |
| Bogdan (10), 2024 | PFOS | Similac Sensitive Baby Dairy Lactose sensitivity, powdered |
| Bogdan (10), 2024 | PFOS | Similac Soy Isomil Soy Lactose intolerance/galacosemia, powdered |
| Bogdan (10), 2024 | PFOS | Similac Neosure Premature Post-Discharge Dairy Premature infants, powdered |
| Bogdan (10), 2024 | PFOS | Up and Up Soy Powder Infant Formula Soy Dairy/Lactose intolerance and Galactosemia, powdered |
| Bogdan (10), 2024 | PFOS | Up and Up Gentle Infant Formula Dairy Lactose sensitivity, powdered |
| Bogdan (10), 2024 | PFOS | Bobbie Organic Infant Formula Dairy Standard, powdered |
| Bogdan (10), 2024 | PFOS | Earth’s Best Organic Dairy Infant Formula Dairy Standard, powdered |
| Bogdan (10), 2024 | PFOS | Earth’s Best Non-GMO Soy Infant Formula Soy Dairy/Lactose intolerance and Galactosemia, powdered |
| Bogdan (10), 2024 | PFOS | Enfamil Gentlease Dairy Lactose sensitivity, powdered |
| Bogdan (10), 2024 | PFOS | Enfamil A.R. Dairy Added rice to prevent spit-up, powdered |
| Bogdan (10), 2024 | PFOS | Enfamil EnfaCare NeuroPro Dairy Premature infants, powdered |
| Bogdan (10), 2024 | PFOS | Enfamil Nutramigen Dairy Hypoallergenic; amino acid(hydrolyzed milk protein), powdered |
| Bogdan (10), 2024 | PFOS | Gerber Good Start GentlePro Non-GMO Dairy Protein sensitivity, powdered |
| Bogdan (10), 2024 | PFOS | Gerber Good Start State 1 Soy Non-GMO Soy Dairy/Lactose intolerance, powdered |
| Bogdan (10), 2024 | PFOS | HappyBaby Organic Infant Formula Dairy Standard, powdered |
| Bogdan (10), 2024 | PFOS | Nutricia Neocate Amino Acid Hypoallergernic; amino acid (made from individual amino acids rather than hydrolyzed casein from milk), powdered |
| Bogdan (10), 2024 | PFOS | Similac Advance Dairy Standard, powdered |
| Bogdan (10), 2024 | PFOS | Similac Sensitive Baby Dairy Lactose sensitivity, powdered |
| Bogdan (10), 2024 | PFOS | Similac Soy Isomil Soy Lactose intolerance/galacosemia, powdered |
| Bogdan (10), 2024 | PFOS | Similac Neosure Premature Post-Discharge Dairy Premature infants, powdered |
| Bogdan (10), 2024 | PFOS | Up and Up Soy Powder Infant Formula Soy Dairy/Lactose intolerance and Galactosemia, powdered |
| Bogdan (10), 2024 | PFOS | Up and Up Gentle Infant Formula Dairy Lactose sensitivity, powdered |
| Bogdan (10), 2024 | Perfluorooctanoic acid (PFOA) | Bobbie Organic Infant Formula Dairy Standard, powdered |
| Bogdan (10), 2024 | PFOA | Earth’s Best Organic Dairy Infant Formula Dairy Standard, powdered |
| Bogdan (10), 2024 | PFOA | Earth’s Best Non-GMO Soy Infant Formula Soy Dairy/Lactose intolerance and Galactosemia, powdered |
| Bogdan (10), 2024 | PFOA | Enfamil Gentlease Dairy Lactose sensitivity, powdered |
| Bogdan (10), 2024 | PFOA | Enfamil A.R. Dairy Added rice to prevent spit-up, powdered |
| Bogdan (10), 2024 | PFOA | Enfamil EnfaCare NeuroPro Dairy Premature infants, powdered |
| Bogdan (10), 2024 | PFOA | Enfamil Nutramigen Dairy Hypoallergenic; amino acid(hydrolyzed milk protein), powdered |
| Bogdan (10), 2024 | PFOA | Gerber Good Start GentlePro Non-GMO Dairy Protein sensitivity, powdered |
| Bogdan (10), 2024 | PFOA | Gerber Good Start State 1 Soy Non-GMO Soy Dairy/Lactose intolerance, powdered |
| Bogdan (10), 2024 | PFOA | HappyBaby Organic Infant Formula Dairy Standard, powdered |
| Bogdan (10), 2024 | PFOA | Nutricia Neocate Amino Acid Hypoallergenic; amino acid (made from individual amino acids rather than hydrolyzed casein from milk), powdered |
| Bogdan (10), 2024 | PFOA | Similac Advance Dairy Standard, powdered |
| Bogdan (10), 2024 | PFOA | Similac Sensitive Baby Dairy Lactose sensitivity, powdered |
| Bogdan (10), 2024 | PFOA | Similac Soy Isomil Soy Lactose intolerance/galacosemia, powdered |
| Bogdan (10), 2024 | PFOA | Similac Neosure Premature Post-Discharge Dairy Premature infants, powdered |
| Bogdan (10), 2024 | PFOA | Up and Up Soy Powder Infant Formula Soy Dairy/Lactose intolerance and Galactosemia, powdered |
| Bogdan (10), 2024 | PFOA | Up and Up Gentle Infant Formula Dairy Lactose sensitivity, powdered |
| Bogdan (10), 2024 | PFOA | Bobbie Organic Infant Formula Dairy Standard, powdered |
| Bogdan (10), 2024 | PFOA | Earth’s Best Organic Dairy Infant Formula Dairy Standard, powdered |
| Bogdan (10), 2024 | PFOA | Earth’s Best Non-GMO Soy Infant Formula Soy Dairy/Lactose intolerance and Galactosemia, powdered |
| Bogdan (10), 2024 | PFOA | Enfamil Gentlease Dairy Lactose sensitivity, powdered |
| Bogdan (10), 2024 | PFOA | Enfamil A.R. Dairy Added rice to prevent spit-up, powdered |
| Bogdan (10), 2024 | PFOA | Enfamil EnfaCare NeuroPro Dairy Premature infants, powdered |
| Bogdan (10), 2024 | PFOA | Enfamil Nutramigen Dairy Hypoallergenic; amino acid(hydrolyzed milk protein), powdered |
| Bogdan (10), 2024 | PFOA | Gerber Good Start GentlePro Non-GMO Dairy Protein sensitivity, powdered |
| Bogdan (10), 2024 | PFOA | Gerber Good Start State 1 Soy Non-GMO Soy Dairy/Lactose intolerance, powdered |
| Bogdan (10), 2024 | PFOA | HappyBaby Organic Infant Formula Dairy Standard, powdered |
| Bogdan (10), 2024 | PFOA | Nutricia Neocate Amino Acid Hypoallergenic; amino acid (made from individual amino acids rather than hydrolyzed casein from milk), powdered |
| Bogdan (10), 2024 | PFOA | Similac Advance Dairy Standard, powdered |
| Bogdan (10), 2024 | PFOA | Similac Sensitive Baby Dairy Lactose sensitivity, powdered |
| Bogdan (10), 2024 | PFOA | Similac Soy Isomil Soy Lactose intolerance/galacosemia, powdered |
| Bogdan (10), 2024 | PFOA | Similac Neosure Premature Post-Discharge Dairy Premature infants, powdered |
| Bogdan (10), 2024 | PFOA | Up and Up Soy Powder Infant Formula Soy Dairy/Lactose intolerance and Galactosemia, powdered |
| Bogdan (10), 2024 | PFOA | Up and Up Gentle Infant Formula Dairy Lactose sensitivity, powdered |
| Bogdan (10), 2024 | Perfluorononanoic acid (PFNA) | Bobbie Organic Infant Formula Dairy Standard, powdered |
| Bogdan (10), 2024 | PFNA | Earth’s Best Organic Dairy Infant Formula Dairy Standard, powdered |
| Bogdan (10), 2024 | PFNA | Earth’s Best Non-GMO Soy Infant Formula Soy Dairy/Lactose intolerance and Galactosemia, powdered |
| Bogdan (10), 2024 | PFNA | Enfamil Gentlease Dairy Lactose sensitivity, powdered |
| Bogdan (10), 2024 | PFNA | Enfamil A.R. Dairy Added rice to prevent spit-up, powdered |
| Bogdan (10), 2024 | PFNA | Enfamil EnfaCare NeuroPro Dairy Premature infants, powdered |
| Bogdan (10), 2024 | PFNA | Enfamil Nutramigen Dairy Hypoallergenic; amino acid(hydrolyzed milk protein), powdered |
| Bogdan (10), 2024 | PFNA | Gerber Good Start GentlePro Non-GMO Dairy Protein sensitivity, powdered |
| Bogdan (10), 2024 | PFNA | Gerber Good Start State 1 Soy Non-GMO Soy Dairy/Lactose intolerance, powdered |
| Bogdan (10), 2024 | PFNA | HappyBaby Organic Infant Formula Dairy Standard, powdered |
| Bogdan (10), 2024 | PFNA | Nutricia Neocate Amino Acid Hypoallergenic; amino acid (made from individual amino acids rather than hydrolyzed casein from milk), powdered |
| Bogdan (10), 2024 | PFNA | Similac Advance Dairy Standard, powdered |
| Bogdan (10), 2024 | PFNA | Similac Sensitive Baby Dairy Lactose sensitivity, powdered |
| Bogdan (10), 2024 | PFNA | Similac Soy Isomil Soy Lactose intolerance/galacosemia, powdered |
| Bogdan (10), 2024 | PFNA | Similac Neosure Premature Post-Discharge Dairy Premature infants, powdered |
| Bogdan (10), 2024 | PFNA | Up and Up Soy Powder Infant Formula Soy Dairy/Lactose intolerance and Galactosemia, powdered |
| Bogdan (10), 2024 | PFNA | Up and Up Gentle Infant Formula Dairy Lactose sensitivity, powdered |
| Bogdan (10), 2024 | PFNA | Bobbie Organic Infant Formula Dairy Standard, powdered |
| Bogdan (10), 2024 | PFNA | Earth’s Best Organic Dairy Infant Formula Dairy Standard, powdered |
| Bogdan (10), 2024 | PFNA | Earth’s Best Non-GMO Soy Infant Formula Soy Dairy/Lactose intolerance and Galactosemia, powdered |
| Bogdan (10), 2024 | PFNA | Enfamil Gentlease Dairy Lactose sensitivity, powdered |
| Bogdan (10), 2024 | PFNA | Enfamil A.R. Dairy Added rice to prevent spit-up, powdered |
| Bogdan (10), 2024 | PFNA | Enfamil EnfaCare NeuroPro Dairy Premature infants, powdered |
| Bogdan (10), 2024 | PFNA | Enfamil Nutramigen Dairy Hypoallergenic; amino acid(hydrolyzed milk protein), powdered |
| Bogdan (10), 2024 | PFNA | Gerber Good Start GentlePro Non-GMO Dairy Protein sensitivity, powdered |
| Bogdan (10), 2024 | PFNA | Gerber Good Start State 1 Soy Non-GMO Soy Dairy/Lactose intolerance, powdered |
| Bogdan (10), 2024 | PFNA | HappyBaby Organic Infant Formula Dairy Standard, powdered |
| Bogdan (10), 2024 | PFNA | Nutricia Neocate Amino Acid Hypoallergenic; amino acid (made from individual amino acids rather than hydrolyzed casein from milk), powdered |
| Bogdan (10), 2024 | PFNA | Similac Advance Dairy Standard, powdered |
| Bogdan (10), 2024 | PFNA | Similac Sensitive Baby Dairy Lactose sensitivity, powdered |
| Bogdan (10), 2024 | PFNA | Similac Soy Isomil Soy Lactose intolerance/galacosemia, powdered |
| Bogdan (10), 2024 | PFNA | Similac Neosure Premature Post-Discharge Dairy Premature infants, powdered |
| Bogdan (10), 2024 | PFNA | Up and Up Soy Powder Infant Formula Soy Dairy/Lactose intolerance and Galactosemia, powdered |
| Bogdan (10), 2024 | PFNA | Up and Up Gentle Infant Formula Dairy Lactose sensitivity, powdered |
| Bogdan (10), 2024 | Perfluorohexane sulfonate (PFHxS) | Bobbie Organic Infant Formula Dairy Standard, powdered |
| Bogdan (10), 2024 | PFHxS | Earth’s Best Organic Dairy Infant Formula Dairy Standard, powdered |
| Bogdan (10), 2024 | PFHxS | Earth’s Best Non-GMO Soy Infant Formula Soy Dairy/Lactose intolerance and Galactosemia, powdered |
| Bogdan (10), 2024 | PFHxS | Enfamil Gentlease Dairy Lactose sensitivity, powdered |
| Bogdan (10), 2024 | PFHxS | Enfamil A.R. Dairy Added rice to prevent spit-up, powdered |
| Bogdan (10), 2024 | PFHxS | Enfamil EnfaCare NeuroPro Dairy Premature infants, powdered |
| Bogdan (10), 2024 | PFHxS | Enfamil Nutramigen Dairy Hypoallergenic; amino acid (hydrolyzed milk protein), powdered |
| Bogdan (10), 2024 | PFHxS | Gerber Good Start GentlePro Non-GMO Dairy Protein sensitivity, powdered |
| Bogdan (10), 2024 | PFHxS | Gerber Good Start State 1 Soy Non-GMO Soy Dairy/Lactose intolerance, powdered |
| Bogdan (10), 2024 | PFHxS | HappyBaby Organic Infant Formula Dairy Standard, powdered |
| Bogdan (10), 2024 | PFHxS | Nutricia Neocate Amino Acid Hypoallergenic; amino acid (made from individual amino acids rather than hydrolyzed casein from milk), powdered |
| Bogdan (10), 2024 | PFHxS | Similac Advance Dairy Standard, powdered |
| Bogdan (10), 2024 | PFHxS | Similac Sensitive Baby Dairy Lactose sensitivity, powdered |
| Bogdan (10), 2024 | PFHxS | Similac Soy Isomil Soy Lactose intolerance/galacosemia, powdered |
| Bogdan (10), 2024 | PFHxS | Similac Neosure Premature Post-Discharge Dairy Premature infants, powdered |
| Bogdan (10), 2024 | PFHxS | Up and Up Soy Powder Infant Formula Soy Dairy/Lactose intolerance and Galactosemia, powdered |
| Bogdan (10), 2024 | PFHxS | Up and Up Gentle Infant Formula Dairy Lactose sensitivity, powdered |
| Bogdan (10), 2024 | PFHxS | Bobbie Organic Infant Formula Dairy Standard, powdered |
| Bogdan (10), 2024 | PFHxS | Earth’s Best Organic Dairy Infant Formula Dairy Standard, powdered |
| Bogdan (10), 2024 | PFHxS | Earth’s Best Non-GMO Soy Infant Formula Soy Dairy/Lactose intolerance and Galactosemia, powdered |
| Bogdan (10), 2024 | PFHxS | Enfamil Gentlease Dairy Lactose sensitivity, powdered |
| Bogdan (10), 2024 | PFHxS | Enfamil A.R. Dairy Added rice to prevent spit-up, powdered |
| Bogdan (10), 2024 | PFHxS | Enfamil EnfaCare NeuroPro Dairy Premature infants, powdered |
| Bogdan (10), 2024 | PFHxS | Enfamil Nutramigen Dairy Hypoallergenic; amino acid(hydrolyzed milk protein), powdered |
| Bogdan (10), 2024 | PFHxS | Gerber Good Start GentlePro Non-GMO Dairy Protein sensitivity, powdered |
| Bogdan (10), 2024 | PFHxS | Gerber Good Start State 1 Soy Non-GMO Soy Dairy/Lactose intolerance, powdered |
| Bogdan (10), 2024 | PFHxS | HappyBaby Organic Infant Formula Dairy Standard, powdered |
| Bogdan (10), 2024 | PFHxS | Nutricia Neocate Amino Acid Hypoallergenic; amino acid (made from individual amino acids rather than hydrolyzed casein from milk), powdered |
| Bogdan (10), 2024 | PFHxS | Similac Advance Dairy Standard, powdered |
| Bogdan (10), 2024 | PFHxS | Similac Sensitive Baby Dairy Lactose sensitivity, powdered |
| Bogdan (10), 2024 | PFHxS | Similac Soy Isomil Soy Lactose intolerance/galacosemia, powdered |
| Bogdan (10), 2024 | PFHxS | Similac Neosure Premature Post-Discharge Dairy Premature infants, powdered |
| Bogdan (10), 2024 | PFHxS | Up and Up Soy Powder Infant Formula Soy Dairy/Lactose intolerance and Galactosemia, powdered |
| Bogdan (10), 2024 | PFHxS | Up and Up Gentle Infant Formula Dairy Lactose sensitivity, powdered |
| Ikem (11), 2002 | Arsenic (As) (total) | Milk based powdered formula |
| Ikem (11), 2002 | Pb | Milk based powdered formula |
| Ikem (11), 2002 | Cadmium (Cd) | Milk based powdered formula |
| Ikem (11), 2002 | Mercury (Hg) (total) | Milk based powdered formula |
| Ikem (11), 2002 | As (total) | Soy based powdered formula |
| Ikem (11), 2002 | Pb | Soy based powdered formula |
| Ikem (11), 2002 | Cd | Soy based powdered formula |
| Ikem (11), 2002 | Hg (total) | Soy based powdered formula |
| Jackson (12), 2012 | As (total) | Brand F: dairy, no rice 2 |
| Jackson (12), 2012 | As (total) | Brand D: dairy, no rice 2 |
| Jackson (12), 2012 | As (total) | Brand F: dairy, no rice 1 |
| Jackson (12), 2012 | As (total) | Brand A: dairy, no rice |
| Jackson (12), 2012 | As (total) | Brand B: dairy, no rice |
| Jackson (12), 2012 | As (total) | Brand B: dairy, rice 1 |
| Jackson (12), 2012 | As (total) | Brand B: no dairy, no rice 1 |
| Jackson (12), 2012 | As (total) | Brand C: dairy, no rice |
| Jackson (12), 2012 | As (total) | Brand B: dairy, rice 2 |
| Jackson (12), 2012 | As (total) | Brand A: no dairy, no rice 2 |
| Jackson (12), 2012 | As (total) | Brand D: dairy, no rice 1 |
| Jackson (12), 2012 | As (total) | Brand E: no dairy, no rice |
| Jackson (12), 2012 | As (total) | Brand A: no dairy, no rice 1 |
| Jackson (12), 2012 | As (total) | Brand B: no dairy, no rice 2 |
| Jackson (12), 2012 | As (total) | Brand A: no dairy, rice |
| Lamm (8), 1973 | Pb | Composite of 5 brands of infant formula concentrate in standard stiff, seemed cans |
| Lamm (7), 1974 | Pb | Composite of 2 brands of ready to serve infant formula |
| Lamm (7), 1974 | Pb | Composite of 2 brands of infant formula from concentrate |
| Munro (3), 1987 | Pb | Enfamil concentrated liquid infant formula (Mead Johnson, Evansville, IN) purchased in Las Cruces, NM; cans were made of steel and had soldered |
| Murthy (9), 1971 | Cd | Modified milk infant formula 2 |
| Murthy (9), 1971 | Cd | Lamb meat infant formula |
| Murthy (9), 1971 | Cd | Modified milk infant formula 1 |
| Murthy (9), 1971 | Cd | Soya infant formula |
| Murthy (9), 1971 | Pb | Modified milk infant formula 1 |
| Murthy (9), 1971 | Pb | Modified milk infant formula 2 |
| Murthy (9), 1971 | Pb | Lamb meat infant formula |
| Murthy (9), 1971 | Pb | Soya infant formula |
| Olu-Owolabi (13), 2007 | As (total) | Enfamil ProSobee Lipil, soy- based powdered milk fortified with Fe in glass bottle |
| Olu-Owolabi (13), 2007 | Pb | Enfamil ProSobee Lipil, soy- based powdered milk fortified with Fe in glass bottle |
| Olu-Owolabi (13), 2007 | Cd | Enfamil ProSobee Lipil, soy- based powdered milk fortified with Fe in glass bottle |
| Rabinowitz (4), 1985 | Pb | Composite (i.e., Similac, Enfamil, and Isomil) |
| Ryu (5), 1983 | Pb | Different milk-based and soy protein isolate-based formulas |
| Ryu (5), 1983 | Pb | commercially available milk-based formula supplied in quart cans |
| Tao (14), 2008 | PFOA | Infant formulas Washington, DC |
| Tao (14), 2008 | PFOS | Infant formulas Washington, DC |
| Tao (14), 2008 | PFNA | Infant formulas Washington, DC |
| Tao (14), 2008 | PFHxS | Infant formulas Washington, DC |
| TatahMentan,(15), 2024 | As (total) | Similac infant formula with iron milk base powder with rice starch |
| TatahMentan,(15), 2024 | Cd | Similac infant formula with iron milk base powder with rice starch |
| TatahMentan,(15), 2024 | Pb | Similac infant formula with iron milk base powder with rice starch |
| TatahMentan,(15), 2024 | As (total) | Enfamil-infant formula with iron milk-based powder with rice starch |
| TatahMentan,(15), 2024 | Cd | Enfamil-infant formula with iron milk-based powder with rice starch |
| TatahMentan,(15), 2024 | Pb | Enfamil-infant formula with iron milk-based powder with rice starch |
| Thompson (2), 1993 | Pb | Milk-based concentrated liquid formula 2 |
| Thompson (2), 1993 | Pb | Milk-based powder formula 1 |
| Thompson (2), 1993 | Pb | Milk-based concentrated liquid formula 3 |
| Thompson (2), 1993 | Pb | Milk-based ready-to-feed formula 2 |
| Thompson (2), 1993 | Pb | Milk-based concentrated liquid formula 1 |
| Thompson (2), 1993 | Pb | Milk-based ready-to-feed formula 1 |
| Thompson (2), 1993 | Pb | Milk-based powder formula 2 |
| Thompson (2), 1993 | Pb | Milk-based powder formula 3 |
| Thompson (2), 1993 | Pb | Soy-based concentrated liquid formula 2 |
| Thompson (2), 1993 | Pb | Milk-based ready-to-feed formula 3 |
| Thompson (2), 1993 | Pb | Soy-based powder formula 2 |
| Thompson (2), 1993 | Pb | Soy-based ready-to-feed formula 3 |
| Thompson (2), 1993 | Pb | Soy-based ready-to-feed formula 2 |
| Thompson (2), 1993 | Pb | Soy-based ready-to-feed formula 1 |
| Thompson (2), 1993 | Pb | Soy-based powder formula 1 |
| Thompson (2), 1993 | Pb | Soy-based concentrated liquid formula 1 |
| Thompson (2), 1993 | Pb | Soy-based concentrated liquid formula 3 |
| Thompson (2), 1993 | Pb | Soy-based powder formula 3 |
| Vela (16), 2004 | As (total) | Powdered formula 1 with iron |
| Vela (16), 2004 | As (total) | Powdered formula 3 with iron |
| Vela (16), 2004 | As (total) | Powdered formula 2 low iron |
| Vela (16), 2004 | As (total) | Powdered formula 4 with iron |
| Walker Jr. (6), 1980 | Pb | Infant formula brand #1 |
| Walker Jr. (6), 1980 | Pb | Infant formula brand #2 |
| Walker Jr. (6), 1980 | Pb | Infant formula brand #3 |
| Walker Jr. (6), 1980 | Pb | Infant formula brand #4 |
| Walker Jr. (6), 1980 | Pb | Infant formula brand #6 |
| Walker Jr. (6), 1980 | Pb | Infant formula brand #7 |
| Walker Jr. (6), 1980 | Pb | Infant formula brand #10 |
| Walker Jr. (6), 1980 | Pb | Infant formula brand #9 |
| Walker Jr,(6) 1980 | Pb | Infant formula brand #8 |
| Walker Jr. (6), 1980 | Pb | Infant formula brand #11 |
| Walker Jr. (6), 1980 | Pb | Infant formula brand #5 |

As, arsenic; Cd, cadmium; Hg, mercury; Pb, lead; PFAS, per- and polyfluoroalkyl substances; PFHxS, Perfluorohexane sulfonate; PFNA, Perfluorononanoic acid; PFOA, Perfluorooctanoic acid; PFOS, Perfluorooctane sulfonate.

## **Supplemental Figure 1.** Analytic framework.

N/A, not applicable.

## **Supplemental Figure 2.** Heat map of published literature^1^ on assessed contaminants in human milk from countries that are ‘high’ or ‘very high’ on the Human Development Index.^2^

| Country | Arsenic | Cadmium | Lead | Mercury | PFAS^3^ | Total for country |
| --- | --- | --- | --- | --- | --- | --- |
| Algeria | 0 | 0 | 0 | 0 | 1 | 1 |
| Argentina | 3 | 2 | 1 | 0 | 0 | 6 |
| Australia | 1 | 2 | 6 | 0 | 0 | 9 |
| Austria | 2 | 5 | 6 | 4 | 2 | 19 |
| Belgium | 0 | 0 | 0 | 0 | 1 | 1 |
| Brazil | 2 | 5 | 8 | 13 | 1 | 29 |
| Canada | 1 | 4 | 5 | 2 | 4 | 16 |
| Chile | 3 | 1 | 1 | 0 | 0 | 5 |
| China | 7 | 7 | 9 | 4 | 14 | 41 |
| Croatia | 2 | 3 | 3 | 3 | 0 | 11 |
| Cyprus | 1 | 1 | 1 | 1 | 0 | 4 |
| Czechia | 0 | 1 | 1 | 0 | 3 | 5 |
| Denmark | 1 | 1 | 1 | 2 | 1 | 6 |
| Ecuador | 1 | 1 | 1 | 1 | 0 | 4 |
| Egypt | 0 | 0 | 1 | 1 | 0 | 2 |
| Finland | 0 | 2 | 0 | 0 | 0 | 2 |
| France | 0 | 1 | 2 | 1 | 3 | 7 |
| Germany | 1 | 9 | 4 | 4 | 5 | 23 |
| Greece | 1 | 1 | 3 | 1 | 0 | 6 |
| Hungary | 2 | 2 | 2 | 3 | 1 | 10 |
| Indonesia | 0 | 0 | 1 | 1 | 0 | 2 |
| Iran (Islamic Republic of) | 10 | 12 | 15 | 14 | 0 | 51 |
| Ireland | 0 | 0 | 0 | 0 | 2 | 2 |
| Italy | 4 | 7 | 9 | 7 | 2 | 29 |
| Japan | 2 | 5 | 4 | 5 | 2 | 18 |
| Jordan | 1 | 1 | 1 | 0 | 1 | 4 |
| Kuwait | 0 | 0 | 1 | 0 | 0 | 1 |
| Latvia | 1 | 1 | 1 | 0 | 0 | 3 |
| Lebanon | 1 | 1 | 1 | 0 | 2 | 5 |
| Mexico | 4 | 1 | 4 | 2 | 0 | 11 |
| Mongolia | 0 | 0 | 0 | 0 | 1 | 1 |
| Multiple, not reported | 0 | 0 | 0 | 1 | 0 | 1 |
| Netherlands | 0 | 0 | 0 | 1 | 1 | 2 |
| New Zealand | 0 | 1 | 1 | 0 | 0 | 2 |
| Norway | 2 | 1 | 1 | 2 | 6 | 12 |
| Palestine | 0 | 1 | 1 | 0 | 0 | 2 |
| Peru | 1 | 0 | 1 | 0 | 0 | 2 |
| Philippines | 1 | 1 | 1 | 2 | 0 | 5 |
| Poland | 5 | 17 | 13 | 3 | 0 | 38 |
| Portugal | 1 | 0 | 1 | 0 | 0 | 2 |
| Romania | 2 | 2 | 3 | 0 | 0 | 7 |
| Russia | 0 | 1 | 2 | 1 | 0 | 4 |
| Saudi Arabia | 0 | 2 | 5 | 8 | 0 | 15 |
| Serbia | 0 | 1 | 1 | 0 | 0 | 2 |
| Slovakia | 0 | 2 | 2 | 4 | 1 | 9 |
| Slovenia | 4 | 5 | 4 | 5 | 0 | 18 |
| South Africa | 1 | 1 | 1 | 0 | 3 | 6 |
| Korea (Republic of) | 0 | 0 | 2 | 3 | 7 | 12 |
| Spain | 7 | 10 | 10 | 8 | 9 | 44 |
| Sweden | 2 | 4 | 5 | 6 | 7 | 24 |
| Thailand | 0 | 1 | 1 | 0 | 1 | 3 |
| Turkiye | 5 | 8 | 9 | 6 | 0 | 28 |
| United Arab Emirates | 1 | 1 | 1 | 1 | 0 | 4 |
| United Kingdom | 2 | 2 | 6 | 2 | 0 | 12 |
| United States | 2 | 2 | 8 | 2 | 4 | 18 |
| Total for contaminant | 87 | 139 | 171 | 124 | 85 | 606 |

^1^There were 332 unique articles. Some articles measured more than 1 contaminant or used data from more than 1 country, and thus each article could contribute more than 1 datapoint to this table.

^2^‘High’ and ‘very high’ developed countries were identified in the Human Development Index which classifies according to: health, education, and quality of life. Available at <https://hdr.undp.org/data-center/human-development-index#/indicies/HDI>.

^3^PFAS, per- and polyfluoroalkyl substances.

## **Supplemental Figure 3.** Heat map of published literature^1^ on assessed contaminants in infant formula from countries that were ‘high’ or ‘very high’ on the Human Development Index^2^.

| Country | Arsenic | Cadmium | Lead | Mercury | PFAS^3^ | Total for country |
| --- | --- | --- | --- | --- | --- | --- |
| Argentina | 1 | 0 | 1 | 0 | 0 | 2 |
| Australia | 1 | 2 | 5 | 0 | 0 | 8 |
| Austria | 1 | 4 | 6 | 2 | 0 | 13 |
| Belarus | 0 | 0 | 1 | 0 | 0 | 1 |
| Belgium | 0 | 1 | 1 | 0 | 0 | 2 |
| Brazil | 2 | 3 | 3 | 1 | 0 | 9 |
| Canada | 1 | 6 | 9 | 1 | 0 | 17 |
| Chile | 1 | 0 | 0 | 0 | 0 | 1 |
| China | 4 | 4 | 4 | 0 | 3 | 15 |
| Czechia | 0 | 0 | 0 | 0 | 1 | 1 |
| Denmark | 1 | 1 | 1 | 0 | 0 | 3 |
| Egypt | 1 | 3 | 3 | 1 | 0 | 8 |
| France | 1 | 3 | 3 | 1 | 0 | 8 |
| Germany | 2 | 4 | 5 | 3 | 1 | 15 |
| Indonesia | 0 | 0 | 0 | 0 | 1 | 1 |
| Iran (Islamic Republic of) | 2 | 6 | 5 | 1 | 0 | 14 |
| Ireland | 1 | 1 | 1 | 0 | 0 | 3 |
| Italy | 4 | 6 | 6 | 2 | 1 | 19 |
| Japan | 0 | 0 | 0 | 1 | 2 | 3 |
| Jordan | 1 | 2 | 2 | 0 | 0 | 5 |
| Latvia | 0 | 0 | 0 | 0 | 1 | 1 |
| Lebanon | 1 | 1 | 1 | 0 | 0 | 3 |
| Malaysia | 1 | 1 | 1 | 0 | 1 | 4 |
| Mexico | 0 | 0 | 1 | 0 | 0 | 1 |
| Netherlands | 1 | 1 | 1 | 0 | 1 | 4 |
| New Zealand | 1 | 2 | 2 | 0 | 0 | 5 |
| Philippines | 1 | 1 | 1 | 0 | 1 | 4 |
| Poland | 4 | 6 | 8 | 3 | 1 | 22 |
| Portugal | 0 | 1 | 1 | 2 | 0 | 4 |
| Romania | 0 | 1 | 1 | 0 | 0 | 2 |
| Saudi Arabia | 2 | 2 | 2 | 1 | 0 | 7 |
| Serbia | 1 | 1 | 1 | 1 | 0 | 4 |
| Singapore | 1 | 1 | 1 | 0 | 0 | 3 |
| Slovakia | 0 | 3 | 3 | 3 | 0 | 9 |
| South Africa | 1 | 1 | 1 | 0 | 2 | 5 |
| Korea (Republic of) | 0 | 0 | 0 | 0 | 1 | 1 |
| Spain | 3 | 10 | 11 | 5 | 2 | 31 |
| Sweden | 1 | 3 | 2 | 1 | 0 | 7 |
| Switzerland | 1 | 1 | 2 | 1 | 0 | 5 |
| Thailand | 1 | 1 | 1 | 0 | 0 | 3 |
| Turkiye | 3 | 4 | 4 | 2 | 0 | 13 |
| Ukraine | 0 | 0 | 1 | 0 | 0 | 1 |
| United Kingdom | 2 | 3 | 4 | 3 | 0 | 12 |
| United States | 5 | 4 | 12 | 1 | 2 | 24 |
| Venezuela | 1 | 1 | 1 | 0 | 0 | 3 |
| Viet Nam | 0 | 0 | 0 | 0 | 1 | 1 |
| Total for contaminant | 55 | 95 | 119 | 36 | 22 | 327 |

^1^There were 124 unique articles. Some articles measured more than 1 contaminant or used data from more than 1 country, and thus each article could contribute more than 1 datapoint to this table.

^2^ ‘High’ and ‘very high’ developed countries were identified in the Human Development Index which classifies according to: health, education, and quality of life. Available at <https://hdr.undp.org/data-center/human-development-index#/indicies/HDI>.

^3^PFAS, per- and polyfluoroalkyl substances.

## **Appendix A.** PRISMA 2020 checklist.

| Section and Topic | Item # | Checklist item | Location where item is reported |
| --- | --- | --- | --- |
| TITLE | | | |
| Title | 1 | Identify the report as a systematic review. | Title Page |
| ABSTRACT | | | |
| Abstract | 2 | See the PRISMA 2020 for Abstracts checklist. | Abstract Section  Complete inclusion/ exclusion criteria and funding were omitted due to space limitations and journal formatting requirements |
| INTRODUCTION | | | |
| Rationale | 3 | Describe the rationale for the review in the context of existing knowledge. | Introduction |
| Objectives | 4 | Provide an explicit statement of the objective(s) or question(s) the review addresses. | Introduction |
| METHODS | | | |
| Eligibility criteria | 5 | Specify the inclusion and exclusion criteria for the review and how studies were grouped for the syntheses. | Supplemental Table 3; Data Synthesis |
| Information sources | 6 | Specify all databases, registers, websites, organisations, reference lists and other sources searched or consulted to identify studies. Specify the date when each source was last searched or consulted. | Supplemental Table 2; Search Strategy |
| Search strategy | 7 | Present the full search strategies for all databases, registers and websites, including any filters and limits used. | Supplemental Table 2 |
| Selection process | 8 | Specify the methods used to decide whether a study met the inclusion criteria of the review, including how many reviewers screened each record and each report retrieved, whether they worked independently, and if applicable, details of automation tools used in the process. | Screening and Data Extraction |
| Data collection process | 9 | Specify the methods used to collect data from reports, including how many reviewers collected data from each report, whether they worked independently, any processes for obtaining or confirming data from study investigators, and if applicable, details of automation tools used in the process. | Screening and Data Extraction |
| Data items | 10a | List and define all outcomes for which data were sought. Specify whether all results that were compatible with each outcome domain in each study were sought (e.g. for all measures, time points, analyses), and if not, the methods used to decide which results to collect. | Screening and Data Extraction |
|  | 10b | List and define all other variables for which data were sought (e.g. participant and intervention characteristics, funding sources). Describe any assumptions made about any missing or unclear information. | Screening and Data Extraction |
| Study risk of bias assessment | 11 | Specify the methods used to assess risk of bias in the included studies, including details of the tool(s) used, how many reviewers assessed each study and whether they worked independently, and if applicable, details of automation tools used in the process. | Risk of Bias |
| Effect measures | 12 | Specify for each outcome the effect measure(s) (e.g. risk ratio, mean difference) used in the synthesis or presentation of results. | Data Synthesis |
| Synthesis methods | 13a | Describe the processes used to decide which studies were eligible for each synthesis (e.g. tabulating the study intervention characteristics and comparing against the planned groups for each synthesis (item #5)). | Critical Appraisal and Data Synthesis |
|  | 13b | Describe any methods required to prepare the data for presentation or synthesis, such as handling of missing summary statistics, or data conversions. | Screening and Data Extraction; Footnotes for tables 2, 4, and 8 |
|  | 13c | Describe any methods used to tabulate or visually display results of individual studies and syntheses. | Data Synthesis |
|  | 13d | Describe any methods used to synthesize results and provide a rationale for the choice(s). If meta-analysis was performed, describe the model(s), method(s) to identify the presence and extent of statistical heterogeneity, and software package(s) used. | Data Synthesis |
|  | 13e | Describe any methods used to explore possible causes of heterogeneity among study results (e.g. subgroup analysis, meta-regression). | Data Synthesis |
|  | 13f | Describe any sensitivity analyses conducted to assess robustness of the synthesized results. | Data Synthesis |
| Reporting bias assessment | 14 | Describe any methods used to assess risk of bias due to missing results in a synthesis (arising from reporting biases). | Risk of Bias |
| Certainty assessment | 15 | Describe any methods used to assess certainty (or confidence) in the body of evidence for an outcome. | Certainty of Evidence |
| RESULTS | | | |
| Study selection | 16a | Describe the results of the search and selection process, from the number of records identified in the search to the number of studies included in the review, ideally using a flow diagram. | Search Results; Figure 1 and 3 |
|  | 16b | Cite studies that might appear to meet the inclusion criteria, but which were excluded, and explain why they were excluded. | Supplemental Table 6 and 9 |
| Study characteristics | 17 | Cite each included study and present its characteristics. | Table 1 and 6; Supplemental Table 10 |
| Risk of bias in studies | 18 | Present assessments of risk of bias for each included study. | Table 3 and 7 |
| Results of individual studies | 19 | For all outcomes, present, for each study: (a) summary statistics for each group (where appropriate) and (b) an effect estimate and its precision (e.g. confidence/credible interval), ideally using structured tables or plots. | Tables 2, 4, and 8;  Results |
| Results of syntheses | 20a | For each synthesis, briefly summarise the characteristics and risk of bias among contributing studies. | Table 3 and 7;  Throughout results |
|  | 20b | Present results of all statistical syntheses conducted. If meta-analysis was done, present for each the summary estimate and its precision (e.g. confidence/credible interval) and measures of statistical heterogeneity. If comparing groups, describe the direction of the effect. | N/A; narrative synthesis |
|  | 20c | Present results of all investigations of possible causes of heterogeneity among study results. | Throughout the results and discussion |
|  | 20d | Present results of all sensitivity analyses conducted to assess the robustness of the synthesized results. | Results |
| Reporting biases | 21 | Present assessments of risk of bias due to missing results (arising from reporting biases) for each synthesis assessed. | Results; Table 3 and 7 |
| Certainty of evidence | 22 | Present assessments of certainty (or confidence) in the body of evidence for each outcome assessed. | Table 5 and 9 |
| DISCUSSION | | | |
| Discussion | 23a | Provide a general interpretation of the results in the context of other evidence. | Throughout the Discussion |
|  | 23b | Discuss any limitations of the evidence included in the review. | Throughout the Discussion |
|  | 23c | Discuss any limitations of the review processes used. | Second to last paragraph of Discussion |
|  | 23d | Discuss implications of the results for practice, policy, and future research. | Throughout the Discussion |
| OTHER INFORMATION | | | |
| Registration and protocol | 24a | Provide registration information for the review, including register name and registration number, or state that the review was not registered. | Methods |
|  | 24b | Indicate where the review protocol can be accessed, or state that a protocol was not prepared. | Methods |
|  | 24c | Describe and explain any amendments to information provided at registration or in the protocol. | Deviations from Protocol |
| Support | 25 | Describe sources of financial or non-financial support for the review, and the role of the funders or sponsors in the review. | Acknowledgements |
| Competing interests | 26 | Declare any competing interests of review authors. | Title page |
| Availability of data, code and other materials | 27 | Report which of the following are publicly available and where they can be found: template data collection forms; data extracted from included studies; data used for all analyses; analytic code; any other materials used in the review. | Data Synthesis |

*From:* Page MJ, McKenzie JE, Bossuyt PM, Boutron I, Hoffmann TC, Mulrow CD, et al. The PRISMA 2020 statement: an updated guideline for reporting systematic reviews. BMJ 2021;372:n71. doi: 10.1136/bmj.n71. This work is licensed under CC BY 4.0. To view a copy of this license, visit <https://creativecommons.org/licenses/by/4.0/>

PRISMA, Preferred Reporting Items for Systematic Reviews and Meta-Analyses.

## **Appendix B.** AMSTAR 2 checklist.

| 1. Did the research questions and inclusion criteria for the review include the components of PICO? | | | |
| --- | --- | --- | --- |
| For yes:   - Population - Intervention - Comparator group - Outcome | Optional (recommended):   - Timeframe for follow-up | Final rating:   - Yes | Location:   - Search strategy - Supplemental table 3 |
| 2. Did the report of the review contain an explicit statement that the review methods were established prior to the conduct of the review and did the report justify any significant deviations from the protocol? | | | |
| For partial yes:  The authors state that they had a written protocol or guide that included ALL the following   - Review question(s) - A search strategy - Inclusion/exclusion criteria - A risk of bias assessment | For yes:  As for partial yes, plus the protocol should be registered and should also have specified:   - A meta-analysis/synthesis plan, if appropriate, *and* - A plan for investigating causes of heterogeneity - Justification for any deviations from the protocol | Final rating:   - Yes | Location:   - Methods |
| 3. Did the review authors explain their selection of the study designs for inclusion in the review? | | | |
| For yes, the review should satisfy ONE of the following:   - *Explanation for* including only RCTs - OR *Explanation for* including only NRSI - OR *Explanation for* including both RCTs and NRSI | | Final rating:   - Yes | Location:   - Search strategy |
| 4. Did the review authors use a comprehensive literature strategy? | | | |
| For partial yes (all the following):   - Searched at least 2 databases (relevant to research question) - Provided key word and/or search strategy - Justified publication restrictions (e.g., language) | For yes, should also have (all the following):   - Searched the reference lists/bibliographies of included studies - Searched trial/study registries - Included/consulted content experts in the field - Where relevant, searched for grey literature - Conducted search within 24 months of completion of the review | Final rating:   - Yes | Location:   - Search strategy - Supplemental table 2 |
| 5. Did the review authors perform study selection in duplicate? | | | |
| For yes, either ONE of the following:   - at least two reviewers independently agreed on selection of eligible studies and archived consensus on which studies to include - OR two reviewers selected a sample of eligible studies and achieved good agreement (at least 80%), with the remainder selected by one reviewer | | Final rating:   - Yes | Location:   - Screening and data extraction |
| 6. Did the review authors perform data extraction in duplicate? | | | |
| For yes, either ONE of the following:   - At least two reviewers achieved consensus on which data to extract from included studies - OR two reviewers extracted data from a sample of eligible studies and achieved good agreement (at least 80%), with the remainder extracted by one reviewer | | Final rating:   - Yes | Location:   - Screening and data extraction |
| 7. Did the review authors provide a list of excluded studies and justify the exclusions? | | | |
| For partial yes:   - Provided a list of all potentially relevant studies that were read in full text form but excluded from review | For yes, must also have:   - Justified the exclusion from the review of each potentially relevant study | Final rating:   - Yes | Location:   - Supplemental table 6 and 9 |
| 8. Did the review authors describe the included studies in adequate detail? | | | |
| For partial yes (all the following):   - Described populations - Described interventions - Described comparators - Described outcomes - Described research designs | For yes, should also have ALL the following:   - Described population in detail - Described intervention and comparator in details (including doses where relevant) - Described study’s setting - Timeframe for follow-up | Final rating:   - Yes | Location:   - Search results - Table 1 and 6 |
| 9. Did the review authors use a satisfactory technique for assessing the risk of bias (RoB) in individual studies that were included in the review? | | | |
| RCTs | | | |
| For partial yes, must have assessed RoB from:   - Unconcealed allocation, *and* - Lack of blinding of patients and assessors when assessing outcomes (unnecessary for objective outcomes such as all-cause mortality) | For yes, must also have assessed RoB from:   - Allocation sequence that was not truly random, *and* - Selection of the reported result from among multiple measurements or analyses of a specified outcome | Final rating:   - Includes only NRSI | Location:   - N/A |
| NRSI | | | |
| For partial yes, must have assessed RoB from:   - Confounding, *and* - Selection bias | For yes, must also have assessed RoB:   - Methods used to ascertain exposures and outcomes, *and* - Selection of the reported result from among multiple measurements or analyses of a specified outcome | Final rating:   - Yes | Location:   - Risk of bias - Throughout results - Table 3 and 7 |
| 10. Did the review authors report on the sources of funding for the studies included in the review? | | | |
| For yes:   - Must have reported on the sources of funding for individual studies included in the review. Note: Reporting that the reviewers look for this information, but it was not reported by study authors also qualifies | | Final rating:   - Yes | Location:   - Table 1 and 6 |
| 11. If meta-analysis was performed did the review authors use appropriate methods for statistical combination of results? | | | |
| RCTs | | | |
| For yes:   - The authors justified combining the data in a meta-analysis - AND they used an appropriate weighted technique to combine study results and adjusted for heterogeneity if present - AND investigated the causes of any heterogeneity | | Final rating:   - No meta-analysis conducted | Location:   - N/A |
| NRSI | | | |
| For yes:   - The authors justified combining the data in a meta-analysis - AND they used an appropriate weighted technique to combine study results, adjusting for heterogeneity if present - AND they statistically combined effect estimates from NRSI that were adjusted for confounding, rather than combining raw rata, or justified combining raw data when adjusted effect estimates were not available - AND they reported separate summary estimates for RCTs and NRSI separately when both were included in the review | | Final rating:   - No meta-analysis conducted | Location:   - N/A |
| 12. If meta-analysis was performed, did the review authors assess the potential impact of RoB in individual studies on the results of the meta-analysis or other evidence synthesis? | | | |
| For yes:   - Included only low risk of bias RCTs - OR, if the pooled estimate was based on RCTs and/or NRSI at variable RoB, the authors performed analyses to investigate possible impact of RoB on summary estimates of effect | | Final rating:   - No meta-analysis conducted | Location:   - N/A |
| 13. Did the review authors account for RoB in individual studies when interpreting/discussing the results of the review? | | | |
| For yes:   - Included only low risk of bias RCTs - OR, if RCTs with moderate or high RoB, or NRSI were included the review provided a discussion with the likely impact of RoB on the results | | Final rating:   - Yes | Location:   - Throughout results and discussion |
| 14. Did the review authors provide a satisfactory explanation for, and discussion of, any heterogeneity observed in the results of the review? | | | |
| For yes:   - There was no significant heterogeneity in the results - OR if heterogeneity was present the authors performed an investigation of sources of any heterogeneity in the results and discussed the impact of this on the results of the review | | Final rating:   - Yes | Location:   - Throughout results and discussion |
| 15. If they performed quantitative synthesis did the review authors carry out an adequate investigation of publication bias (small study bias) and discuss its likely impact on the results of the review? | | | |
| For yes:   - Performed graphical or statistical tests for publication bias and discussed the likelihood and magnitude of impact of publication bias | | Final rating:   - No meta-analysis conducted | Location:   - N/A |
| 16. Did the review authors report any potential sources of conflict of interest, including any funding they received for conducting the review? | | | |
| For yes:   - The authors reported no competing interests OR - The authors described their funding sources and how they managed potential conflicts of interest | | Final rating:   - Yes | Location:   - Title page |

From Shea BJ, Reeves BC, Wells G, et al. Amstar 2: A critical appraisal tool for systematic reviews that include randomised or non-randomised studies of healthcare interventions, or both. *BMJ*. Sep 21 2017;358:j4008. doi:10.1136/bmj.j4008

AMSTAR 2, A MeaSurement Tool to Assess systematic Reviews 2; N/A, not applicable; NRSI, non-randomized studies of interventions; PICO; population, intervention, comparator group, outcome; RCTs, Randomized controlled trials; RoB, risk of bias.

## **References**

1.	Baum CR, Shannon MW. The lead concentration of reconstituted infant formula. *J Toxicol Clin Toxicol*. 1997;35(4):371-5. doi:10.3109/15563659709043369

2.	Thompson JJ. Determination of ultratrace levels of lead in infant formula by isotope dilution inductively coupled plasma mass spectrometry. *J AOAC Int*. Nov-Dec 1993;76(6):1378-84.

3.	Munro J. Determination of six elements in infant formula by flame and furnace atomic adsorption spectroscopy. *Atomic Spectroscopy*. 1987;8:92-95.

4.	Rabinowitz M, Leviton A, Needleman H. Lead in milk and infant blood: A dose-response model. *Arch Environ Health*. Sep-Oct 1985;40(5):283-6. doi:10.1080/00039896.1985.10545933

5.	Ryu JE, Ziegler EE, Nelson SE, Fomon SJ. Dietary intake of lead and blood lead concentration in early infancy. *Am J Dis Child*. Sep 1983;137(9):886-91.

6.	Walker Jr B. Lead content of milk and infant formula. *Journal of Food Protection*. 1980;43(3):178-179.

7.	Lamm SH, Rosen JF. Lead contamination in milks fed to infants: 1972-1973. *Pediatrics*. Feb 1974;53(2):137-41.

8.	Lamm S, Cole B, Glynn K, Ullmann W. Lead content of milks fed to infants--1971-1972. *N Engl J Med*. Sep 13 1973;289(11):574-5. doi:10.1056/NEJM197309132891109

9.	Murthy GK, Rhea US. Cadmium, copper, iron, lead, manganese, and zinc in evaporated milk, infant products, and human milk. *J Dairy Sci*. Jul 1971;54(7):1001-5. doi:10.3168/jds.S0022-0302(71)85962-3

10.	Bogdan AR, Klos KS, Greene CW, Huset CA, Barry KM, Goeden HM. Per- and polyfluoroalkyl substances (PFAS) in powdered infant formula: Potential exposures and health risks. *Journal of Environmental Exposure Assessment*. 2024;3(2):14.

11.	Ikem A, Nwankwoala A, Odueyungbo S, Nyavor K, Egiebor N. Levels of 26 elements in infant formula from USA, UK, and Nigeria by microwave digestion and ICP-OES. *Food Chemistry*. 2002;77:439-447.

12.	Jackson BP, Taylor VF, Punshon T, Cottingham KL. Arsenic concentration and speciation in infant formulas and first foods. *Pure Appl Chem*. 2012;84(2):215-223. doi:10.1351/PAC-CON-11-09-17

13.	Olu-Owolabi BIF, S. O.; Adebowale, K. O.; Onianwa, P. C.. Proximate and elemental composition and their estimated daily intake in infant formulae from developed and developing countries: A comparative analysis. *Journal of Food, Agriculture & Environment*. 2007;5:40-44.

14.	Tao L, Ma J, Kunisue T, Libelo EL, Tanabe S, Kannan K. Perfluorinated compounds in human breast milk from several asian countries, and in infant formula and dairy milk from the United States. *Environ Sci Technol*. Nov 15 2008;42(22):8597-602. doi:10.1021/es801875v

15.	TatahMentan M, Nyachoti S, Godebo TR. Elemental composition of toxic and essential elements in rice-based baby foods from the United States and other countries: A probabilistic risk analysis. *Food Chem Toxicol*. Jun 2024;188:114677. doi:10.1016/j.fct.2024.114677

16.	Vela NP, Heitkemper DT. Total arsenic determination and speciation in infant food products by ion chromatography-inductively coupled plasma-mass spectrometry. *J AOAC Int*. Jan-Feb 2004;87(1):244-52.
